# The Application of Traditional Chinese Medicine Injection on Patients with Acute Coronary Syndrome during the Perioperative Period of Percutaneous Coronary Intervention: A Systematic Review and Meta-Analysis of Randomized Controlled Trials

**DOI:** 10.1155/2020/3834128

**Published:** 2020-05-18

**Authors:** Zhaofeng Shi, Chen Zhao, Jiayuan Hu, Qianqian Dai, Manke Guan, Changming Zhong, Guihua Tian, Hongcai Shang

**Affiliations:** ^1^Key Laboratory of Chinese Internal Medicine of Ministry of Education and Beijing, Beijing 100700, China; ^2^Dongzhimen Hospital, Beijing University of Chinese Medicine, Beijing 100700, China; ^3^Institute of Basic Research in Clinical Medicine, China Academy of Chinese Medical Sciences, Beijing 100700, China; ^4^International Evidence-Based Research Institute of Chinese Medicine, Beijing University of Chinese Medicine, Beijing 100029, China

## Abstract

**Introduction:**

TCMI with the effect of *Liqihuoxue* and *Yiqihuoxue* has been applied as complementary therapies during the perioperative period of PCI for patients with ACS, while the recommended time points and plans of TCMI are still short of the support of evidence-based medicine.

**Methods:**

A systematic review and meta-analysis was conducted to evaluate the clinical efficacy and safety of TCMI on patients with ACS during the perioperative period of PCI. RCTs were searched based on standardized searching rules in seven medical databases from the inception up to August 2019. Two reviewers conducted the study selection, data extraction, and quality analysis independently. Data were analysed with the support of software *RevMan* and *Stata*.

**Results:**

A total of 68 articles with 6,043 patients were enrolled. The result of meta-analysis showed that the TCMI combined with western medicine was superior to the western medicine alone on clinical efficiency (before the PCI, before and after the PCI, or overall, *P* < 0.05), the occurrence of MACE (myocardial infarction and stenocardia: before the PCI, before and after the PCI, or overall, *P* < 0.05; arrhythmia: before and after the PCI, *P* < 0.05), and the level of inflammatory factors (hs-CRP: before the PCI, before and after the PCI, or overall, *P* < 0.05; IL-6: after the PCI, *P* < 0.05). The TCMI with the effect of *Liqihuoxue* obtained more support compared with *Yiqihuoxue* based on the result of meta-analysis.

**Conclusions:**

TCMI with the effect of *Liqihuoxue* or *Yiqihuoxue* combined with western medicine generally showed the potential advantage on the treatment of ACS during the perioperative period of PCI. However, the optimal time point of intervention and recommended plan based on the effect still needs more clinical evidence. We consider that the research of precise and standardized application of TCMI will be a promising direction for TCM in the future.

## 1. Introduction

Acute coronary syndrome (ACS), which is caused by rupture or erosion of atherosclerotic plaque in the coronary artery or fresh thrombosis, can be classified as unstable angina (UA), non-ST-elevation myocardial infarction (NSTEMI), and ST-elevation myocardial infarction (STEMI) based on the electrocardiographic changes and cardiac biomarker [1]. In most developed countries, the incidence of ACS is declining in the past 30 years [2, 3]; however, it is still increasing in China with each passing year and the vast majority of patients with ACS was first diagnosed and received treatment in the emergency department [4]. There are currently 290 million cardiovascular patients in China, and the number of patients with ACS is expected to reach 22.6 million by 2030 [5].

The clinical manifestation of ACS patients is variable, with the most common symptom such as chest pain or chest tightness [6]. However, some patients such as elderly women and diabetes may not have typical symptoms. The diagnosis of ACS can be defined as the increase in troponin levels with at least one value > 99th percentile of upper reference limit and plus the at least one part of diagnostic evidence from the symptom of myocardial ischemia, electrocardiograph (ECG), and image finding [7]. The risk stratification for ACS is a prerequisite on the establishment of clinical strategy, which means only by applying an appropriate risk stratification, a preferable therapeutic efficiency can be achieved. Some publications have identified new biomarkers for risk stratification of patients with ACS, including gut-microbiota-dependent trimethylamine N-oxide [8], microRNAs (26b-5, 660-5, and 320a) [9], and acute myocardial infarction (AMI) telomere length in peripheral blood cells [10]. As for the clinical score for risk stratification, the PRECISE-DAPT (dual antiplatelet therapy) [11] and the CRUSADE bleeding score [12] has proved its value on the prediction of the risk of bleeding events; meanwhile, the Global Registry of Acute Coronary Events (GRACE) score and the thrombolysis in myocardial infarction (TIMI) score have identified the effect on the evaluation of ischemia risk [13]. Basic treatments for ACS include dual antiplatelet (such as aspirin and P2Y12 inhibitors) [14], anticoagulant (such as fondaparinux and low-molecular-weight heparin) [15], and anti-ischemic (such as beta-blockers) [16] therapies. The treatment of revascularization includes the percutaneous coronary intervention (PCI), thrombolytic therapy (tissue plasminogen activator), and coronary artery bypass grafting (CABG) [17].

PCI, which owns the immediate effect on revascularizing the infarct-related arteries (IRA), is being widely applied and dramatically improved the prognosis of ACS [18]. In 2015, more than 567,000 patients registered and finished the PCI in China, ranking the second in the world [19]. It should be noticed that this figure reached 753,142 in 2017 based on the report of China Cardiovascular Intervention Forum (CCIF). However, despite the improvement in antithrombotic technology and innovation of revascularizing strategy, the prognosis of PCI for patients with ACS is still unsatisfactory [20], and the incidence of major adverse cardiac events (MACE) is still at a high level [21]. Some PCI-related problems, such as no-reflow, ischemia-reperfusion injury, perioperative myocardial injury (PMI), in-stent restenosis, and stent thrombosis, are difficult to avoid. In the past 30 years, with the development in clinical trials of TCM in China, it has been found that the traditional Chinese medicine injection (TCMI) has a good effect on treating and preventing arrhythmia and reperfusion injury, improving heart function and protecting myocardium [22]. The *Liqihuoxue* and *Yiqihuoxue* are two essential effects of TCMI. According to the theory of TCM, *Qi* is the most basic substance to constitute and maintain human life activities. The stagnation or deficiency of *Qi* will induce the blood stasis, which is basically equivalent to endothelial dysfunction (ETDF), forming an essential pathological basis of cardiovascular disease. *Liqihuoxue* is used in the ACS patients with asthenia syndrome through the function of regulating *Qi* and removing blood stasis, while *Yiqihuoxue* is used for the deficiency syndrome through the function of nourishing *Qi* and removing blood stasis.

The application of TCMI combined with western medicine during the perioperative period of PCI has become a hotspot on the treatment of ACS in China, but the optimal time point of intervention is still a matter of debate and the recommended plan from TCMI with the effect of *Liqihuoxue* or *Yiqihuoxue* is still unknown. Moreover, some clinical centers randomly use the TCMI with the effect of *Liqihuoxue* and *Yiqihuoxue* before or even after PCI. Finding the optimal time point of intervention and providing the therapeutic plan based on the effect of Chinese medicine are necessary for the development of TCM. Given the great variation in previous results, we performed a systematic review and meta-analysis to evaluate the efficacy and safety of TCMI in the treatment of ACS based on the different time points and the effect of *Liqihuoxue* or *Yiqihuoxue*.

## 2. Methods

This research is based on the guideline of PRISMA [23] and followed the instruction from the Cochrane Reviewer Handbook (version 5.1) [24].

### 2.1. Data Sources and Search Methods

Seven electronic medical databases named PubMed, Cochrane Library, Web of Science, EMBASE, the CNKI (Chinese), Wanfang Data (Chinese), and Vip Data (Chinese) were searched from the inception up to August 2019. Articles were included with the language of Chinese or English. The relevant systematic reviews were also temporarily included and analysed for the supplementation of the potentially qualified articles. Emails were sent to authors for the acquirement of the non-full-text articles. The supplemental search was performed in the library of Beijing University of Chinese Medicine and the China Academy of Traditional Chinese Medicine for the acquisition of grey studies. The searching terms, which were conducted and adjusted for the variation in language, contained as follows: acute coronary syndrome, myocardial infarction, acute myocardial infarction, ST-segment elevation myocardial infarction, non-ST-segment elevation myocardial infarction, STEMI, NSTEMI, unstable angina, UA, injection, Chinese patent medicine, traditional Chinese medicine, percutaneous coronary intervention, PCI, and randomized clinical trials.

### 2.2. Eligibility Criteria

The eligibility criteria of inclusion and exclusion were performed by two researchers (MD. Zhaofeng Shi and MM. Qianqian Dai) independently, and the disagreement was resolved by the common discussion or the guidance of the third researcher (Pro. Hongcai Shang).

The eligibility criteria of included studies were suited for the following criteria: (1) RCTs; (2) patients who complied with the diagnostic criteria of ACS based on the guideline of ESC for STEMI [25] or UA/NSTEMI [26]; (3) patients of either gender and of any age who received the PCI, including the PTCA and coronary artery stent implantation (such as bare metal stent and drug eluting stent), within 12 hours from the occurrence of symptoms of myocardial ischemia; (4) patients who received the TCMI with the effect of regulating *Qi* and removing stasis (*Liqihuoxue*) or nourishing *Qi* and removing stasis (*Yiqihuoxue*) based on the guidelines of drug description. TCMI combined with western medicine (dual antiplatelet, anticoagulant, and anti-ischemic) was defined as the experimental group; meanwhile, western medicine alone was as the controlled group; (5) the time point of intervention for TCMI was settled before the PCI (less than 3 hours), after the PCI (more than 3 hours), or before and after the PCI together; (6) the outcome indicators should include at least one of following items: (a) clinical efficiency (including the criteria of complete response, partial response, and invalid response; complete response plus partial response was defined as the total effective response) [27]; (b) MACE (including death, myocardial infarction, hospitalization for unstable angina, transient ischemic attack and stroke, heart failure event, percutaneous coronary intervention, peripheral vascular intervention, and stent thrombosis) [28]; (c) inflammatory factors (CRP, hs-CRP, IL-6, IL-10, IL-18, or TNF-*α*); (d) adverse events resulting from TCMI or western medicine.

Studies were excluded if they met one of the following criteria: (1) non-RCTs (including quasi-RCTs, CCTs, cohort study, case series, and case reports); (2) received the traditional Chinese herbal medicine or TCMI in the controlled group; (3) received the unrelated TCMI, which was not focused on the treatment of ACS, or Chinese herbal medicine in the experimental group; (4) the types of diseases were not compatible with the criteria of ACS (STEMI, USTEMI, and UA); and (5) severe clinical illness, including (a) had active bleeding or the tendency of bleeding; (b) cardiogenic shock, cardiac rupture, or ventricular septal perforation; (c) acute pericarditis, subacute infective endocarditis, or aortic dissection; (d) severe arrhythmia (left bundle branch block, ventricular tachycardia, ventricular flutter, and ventricular fibrillation); and (e) serious disease in the liver, kidney, hematopoietic system, or malignant tumours.

Particularly, it should be highlighted that STEMI, NSTEMI, and UA had many commonalities in the pathogenesis and pathophysiology, which were related to the formation of atherosclerotic plaque. Although the difference among them was the degree of occlusion of coronary artery (STEMI is more seriously than NSTEMI), the long-term prognosis and the severity were similar and the treatment of PCI was of great significance. As for the classifications of stents in the insertion of vessel stents, even though the BVS (bioresorbable vessel scaffold) was no worse than EES (everolimus-eluting stent) in 1-year TLF (target lesion failure) rate, cardiogenic death, and TLR (target lesion revascularization) induced by target vessel MI and ischemia [29], we did not limit the type of stent in the inclusion criteria of this research in view of the current status of PCI in China. Chinese herbal medicine should not be combined with TCMI, even though they had the synergistic effects without interfering with the major function of TCMI. The dosage of the TCMI and western medicine was discrepant in experimental groups or controlled groups, and there was no limitation for the dosage in the selection of research.

### 2.3. Study Selection

The software named *EndNote X8* was used to establish a preliminary literature database which met the requirements of removing duplicates and screening the procedure of selection. Two researchers (MD. Zhaofeng Shi and Prof. Chen Zhao) did the procedure by reading title and abstract based on the previously defined inclusion and exclusion criteria. After obtaining the full-text papers, the researchers read the inclusion and exclusion criteria once again for further screening. If the information of the included papers was incomplete or difficult to be judged during the process of screening, the original author would be contacted by email. If it was difficult to receive a response from the original author, the missing information would be excluded. The third researcher (Prof. Hongcai Shang) did the judgment after the discussion if there was disagreement during the cross-correction.

### 2.4. Data Extraction and Quality Analysis

Two researchers (MM. Changming Zhong and MD. Zhaofeng Shi) extracted data and established a summary table independently, which contained the following items: (1) the name of author and the year of publication, (2) the researching area, (3) sample size, (4) age of patients, (5) other information (such as the past medical history, personal history, and classification of heart function), (6) treatments of experimental and controlled groups, (7) duration of treatments and follow-up, (8) evaluation of outcome indicators and quality assessment, and (9) adverse events of the TCMI. The results were cross-checked in this process, and any disagreement between the results will be resolved after a discussion and judged by the arbiter (Prof. Hongcai Shang).

The quality analysis was performed by two investigators independently (MD. Zhaofeng Shi and MD. Jiayuan Hu), using the tool of the Cochrane Reviewer Handbook 5.1 [24]. This tool was conducted to evaluate the risk bias of included studies across seven domains: (1) random sequence generation (selection bias), (2) allocation concealment (selection bias), (3) blinding of participants and personnel (performance bias), (4) blinding of outcome assessment (detection bias), (5) incomplete outcome data (attrition bias), (6) selective reporting (reporting bias), and (7) other sources of bias (other bias). Researchers would answer these questions with “yes (Y),” “unclear (U),” or “no (N)” to evaluate the degree of risk of bias. If an included research is satisfied with more than four domains, it should be grouped as the low risk of bias; one to four domains should be grouped as the moderate risk of bias; and one or no domain should be grouped as the high risk of bias. The disagreement during this procedure would be resolved after a discussion and judged by the arbiter (Prof. Hongcai Shang). The outcomes above were established as tables and images with the support of software Review Manager (*RevMan, version* 5.3, *the Nordic Cochrane Centre, the Cochrane Collaboration, 2012 Copenhagen, Denmark*).

### 2.5. Statistical Analysis

The data were analysed by the software *RevMan* and *Stata* (*version* 14.0, *StataCorp LP*, *College Station*, *US*). The analysis was conducted after the comparison of outcomes between the experimental and the controlled groups. The *risk ratio* (RR) with 95% *confidence interval* (CI) was calculated for the dichotomous data and the *standard mean difference* (*Std*. MD) or the *mean difference* (MD) with 95% CI was calculated for the continuous data, respectively.

The *χ*^2^ test and the *I*^2^ statistic were conducted to identify and measure the statistical heterogeneity. These methods could provide an estimate of variation which resulted from heterogeneity. The heterogeneity was divided into three levels based on the *I*^2^ statistic outcomes: (1) between 25 and 50% was low, (2) between 50 and 75% was moderate, and (3) above 75% was high. The *P* value lower than 0.05 and *I*^2^ statistic outcome higher than 50% were considered to obtain significant heterogeneity. The heterogeneity source needed to be further explored with the method of subgroup analysis or metaregression analysis. The sample size, research areas, and levels of hospitals were used as the classification for subgroup analysis.

A random-effects model which used the method of *DerSimonian–Laird* (*DS-L*) [30] or *Inverse Variance* (*IV*) was conducted to pool data based on the moderate or high heterogeneity and a fixed-effects model which used the method of *Mantel–Haenszel* (*M-H*) was established to pool data based on the low heterogeneity [31]. The sensitivity analysis was conducted to evaluate the stability of analysis by using different effects model and examining the effects of individual factors on the overall combined effect size. The method of funnel plot and *Egger's* test/*Begg's* test was used to assess the publication bias by the software *RevMan* and *Stata* if an outcome included more than 10 studies [32, 33].

## 3. Results

### 3.1. Study Selection

**Figure 1:**
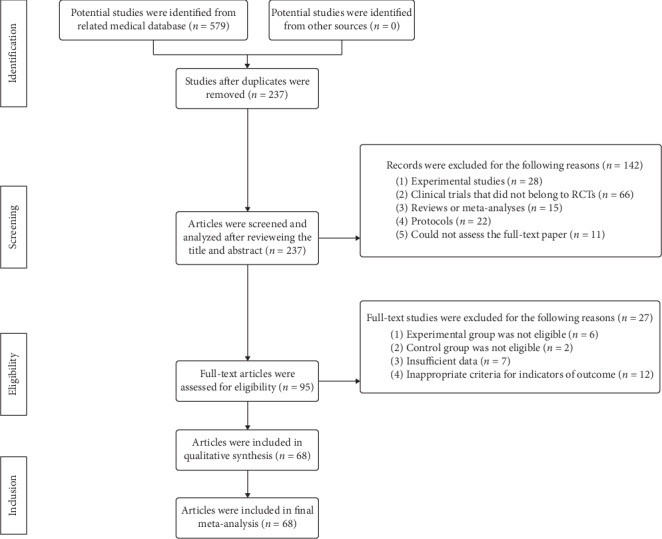
The preferred reporting items for systematic reviews and meta-analyses (PRISMA) flow diagram.

The flow diagram of the screening and selection of potential articles was illustrated in Figure 1. A total of 579 related studies were identified from the medical databases, and 342 studies were ruled out due to the duplication. After the screening of the title and abstract, one hundred and forty-two studies were further excluded for the following reasons: (1) twenty-eight were experimental studies, (2) sixty-six clinical studies did not belong to RCTs, (3) fifteen studies belonged to reviews or meta-analyses, (4) twenty-two studies were protocols, and (5) eleven studies could not obtain the full-text paper. There were 27 studies excluded after the full-text paper reading for the following reasons: (1) the experimental group was not eligible for 6 studies, (2) the controlled group was not eligible for 2 studies, (3) insufficient data were found in 7 studies, and (4) twelve studies had inappropriate criteria for the indicators of outcome. Overall, a total of 68 articles with 6,043 patients were enrolled in this research.

### 3.2. Study Characteristics

A total of 68 studies conformed to the final eligibility criteria and were included in the meta-analysis (Table 1). All studies were randomized clinical trials (RCTs) and fifteen trials among them were multicentred studies, which performed in different hospitals of China [34, 48, 49, 51, 55, 58, 59, 61, 66, 82, 88, 94, 95, 100, 101]. The publishing year of studies was found between 2004 and 2018. The sample size of studies ranged from 38 [46] to 203 [65], and the age range of male and female was between 31 [37] and 84 [41] years old. As for the classification of ACS, only twenty-one studies clearly defined including seven studies for UA [36, 73, 74, 78, 80, 86], eleven studies for STEMI [42, 45, 46, 49, 51, 66, 70, 71, 91, 92, 95], and three for NSTEMI [52, 72, 75]. However, the rest of forty-eight studies did not introduce the classification. The types of TCMI in the experimental group were diversified and listed as follows: injection of *Dazhuhongjingtian* [34–38], *Shuxuetong* [39, 42, 83–89], *Shenmai* [40–44, 46–48], *Danshen* [45, 49], *Danhong* [50–67, 73, 74], *Dengzhanhuasu* [68], *Gualoupi* [69], *Guanxinning* [70, 71], *Safflower yellow* [72, 75], *Safflower* [76–78], *Kudiezi* [79], *Shengmai* [80–82], *Xiangdan* [90], *Xuesaitong* [91–95], *Xueshuantong* [96–100], and *Yiqifumai* [101]. The detailed information of TCMI, which included constituents of TCMI, Latin names of constituents for Chinese medicine, ratios of constituents, specifications clinical use of the TCMI, and Chinese national medicine permission numbers, was well illustrated (see Table S2 and Figures S13–S28 in the Supplementary Materials). The western medicine contained the anticoagulant, antimyocardial ischemia, antiplatelet, lipid-lowering, and antihypertensive treatment. As for the duration of therapy, all included studies except seven [56, 71, 72, 78, 80, 81, 100] clearly reported. The time of follow-up was mentioned in fifteen included studies [43, 44, 46, 48, 51, 53, 58, 71, 75, 76, 79, 95, 97, 99, 100]. It needs to highlight that only fourteen included studies [37, 41–43, 46, 50, 51, 57, 59, 60, 62, 91, 92, 95] reported the adverse events, which focused on the bleeding event, gastrointestinal reaction, and arrhythmia.

### 3.3. Quality Analysis

For the included studies, twenty-two [42, 47, 50, 51, 54, 55, 57, 60, 62, 63, 72, 73, 76, 81, 83–85, 91, 92, 96, 97, 100] mentioned the random sequence generation. No study clearly illustrated or contained the allocation concealment. Only 2 studies [74, 76] introduced the blinding method, which was the sealed envelope method. As for the aspect of incomplete outcome data, no included studies had the attrition bias basically. Only 6 studies [48, 78, 87–90] had the question of existing of other biases (see Figures S1 and S2 and Table S1 in the Supplementary Materials).

### 3.4. Meta-Analysis

#### 3.4.1. Clinical Efficiency

**Figure 2:**
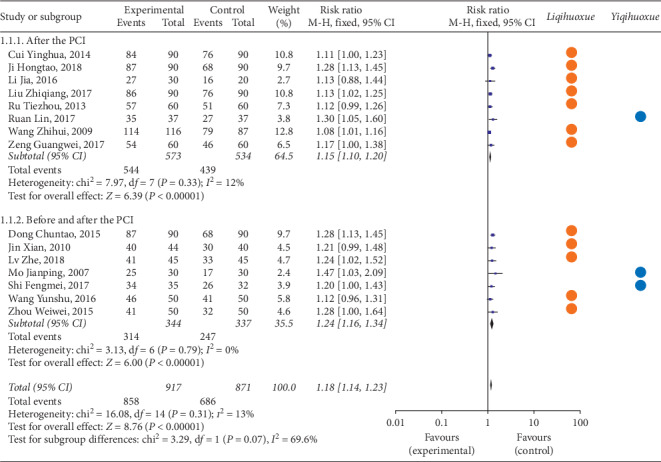
Forest plot of clinical efficiency of TCMI based on the time point of intervention and the effect of *Liqihuoxue* or *Yiqihuoxue*. *Note.*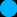 represents the TCMI with the effect of *Liqihuoxue*; 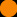 represents the TCMI with the effect of *Yiqihuoxue*.

Figure 2 illustrates the clinical efficiency of TCMI based on the effect of *Yiqihuoxue* or *Liqihuoxue* and the time points of intervention. There were 15 articles including 3,332 participants analysed in the forest plot [34, 35, 40, 41, 51–53, 59, 65, 74, 75, 77, 83, 87, 90]. We extracted 8 articles [34, 35, 41, 51, 52, 59, 65, 87] (2,090 participants) from the 15 studies to compare with the rest of 7 articles [40, 53, 74, 75, 77, 83, 90] (1,242 patients) based on the different time points of intervention during the perioperative period of PCI. The result showed that the clinical efficiency of TCMI combined with the western medicine (experimental group) was superior to the western medicine alone (controlled group) on patients with ACS (before the PCI: RR = 1.15, 95% CI = 1.10 to 1.20, *P* < 0.01; before and after PCI: RR = 1.24, 95% CI = 1.16 to 1.34, *P* < 0.01; overall: RR = 1.18, 95% CI = 1.14 to 1.23, *P* < 0.01). The TCMI with the effect of *Liqihuoxue* [34, 35, 51–53, 59, 65, 74, 75, 77, 83, 87] combined with western medicine was superior to the western medicine in the time points of before and after the PCI and after the PCI. The results of the clinical efficiency between the experimental group and the controlled group had statistical difference. The heterogeneity was small (before the PCI: *P* = 0.33, *I*^2^ = 12%; before and after the PCI: *P* = 0.79, *I*^2^ = 0%; overall: *P* = 0.13, *I*^2^ = 13%), and the fixed-effects model was performed to calculate combined data by the *M-H* test. However, the results could not recommend the best time point of intervention for TCMI on ACS.

#### 3.4.2. MACE

**Figure 3:**
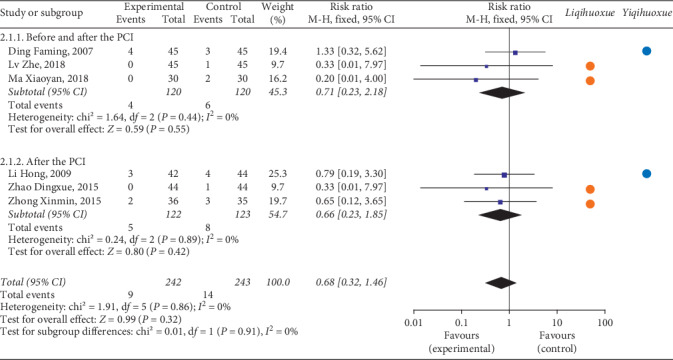
Forest plot of all-cause mortality based on the time point of intervention and the effect of *Liqihuoxue* or *Yiqihuoxue*.

**Figure 4:**
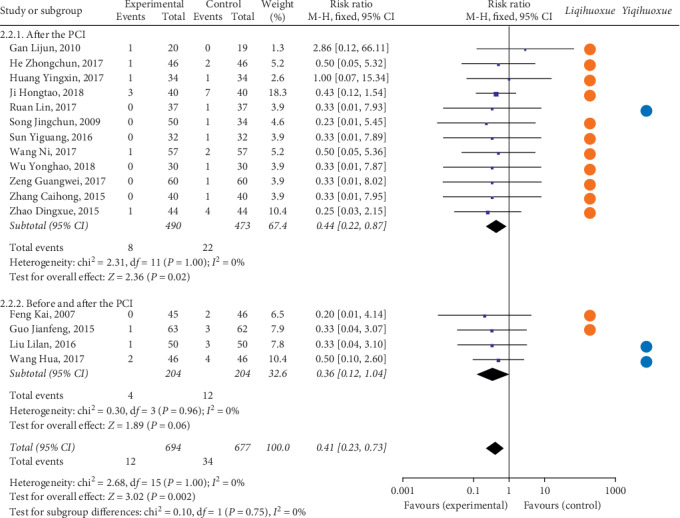
Forest plot of myocardial infarction based on the time point of intervention and the effect of *Liqihuoxue* or *Yiqihuoxue*.

**Figure 5:**
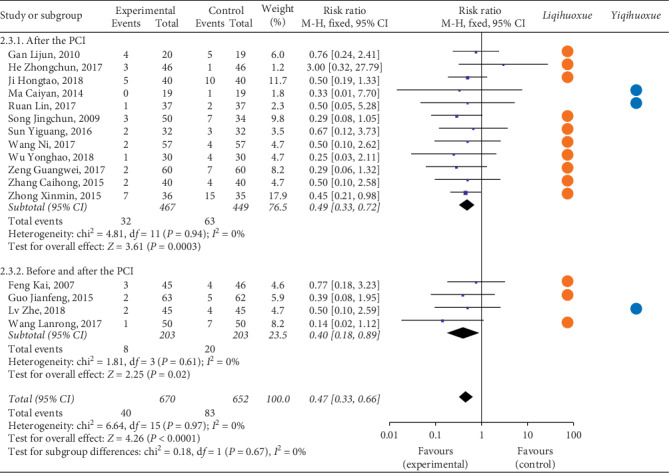
Forest plot of stenocardia based on the time point of intervention and the effect of *Liqihuoxue* or *Yiqihuoxue*.

**Figure 6:**
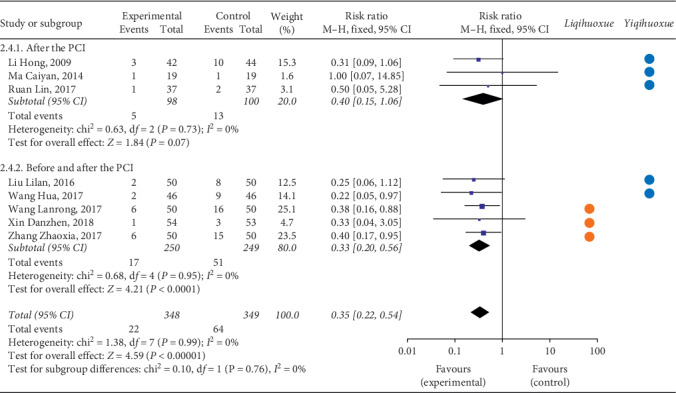
Forest plot of arrhythmia based on the time point of intervention and the effect of *Liqihuoxue* or *Yiqihuoxue*.

Figures 3–6 illustrate the MACE of patients with ACS after the treatment of experimental group and controlled group based on the effect of *Liqihuoxue* or *Yiqihuoxue* and the time point of intervention.

*(1) All-Cause Mortality*. There were 6 articles including 508 participants analysed the all-cause mortality in the forest plot [49, 57, 71, 76, 83, 84] (Figure 3). Three articles [49, 83, 84] with 250 participants received the treatment before and after the PCI compared with the rest of 3 articles [57, 71, 76] with 258 patients received the treatment after the PCI. The meta-analysis showed that the occurrence of all-cause mortality of the experimental group after the PCI, before and after the PCI, and overall was not lower than the controlled group on patients with ACS (before and after the PCI: RR = 0.71, 95% CI = 0.23 to 2.18, *P* = 0.55; after the PCI: RR = 0.66, 95% CI = 0.23 to 1.85, *P* = 0.42; overall: RR = 0.68, 95% CI = 0.32 to 1.46, *P* = 0.32). TCMI with the effect of *Liqihuoxue* or *Yiqihuoxue* [57, 76, 83, 84] did not show the superiority. The heterogeneity was not found (before and after the PCI: *P* = 0.44, *I*^2^ = 0%; after the PCI: *P* = 0.89, *I*^2^ = 0%; overall: *P* = 0.86, *I*^2^ = 0%), and the fixed-effects model was performed by the *M-H* test.

*(2) Myocardial Infraction*. As for the myocardial infraction, twelve articles [34, 41, 50, 51, 76, 89, 95–100] with 993 participants received the treatment after the PCI compared with the 4 articles [43, 44, 58, 67] with 424 patients before and after the PCI (Figure 4). The result illustrated that the occurrence of myocardial infraction of the experimental group was lower than the controlled group based on the intervention of time point after the PCI (RR = 0.44, 95% CI = 0.22 to 0.87, *P* = 0.02). The TCMI with the effect of *Liqihuoxue* [34, 50, 51, 58, 67, 76, 89, 95–100] showed the superiority on the time point after the PCI. The heterogeneity was also not found (after the PCI: *P* = 1.00, *I*^2^ = 0%; before and after the PCI: *P* = 0.96, *I*^2^ = 0%; overall: *P* = 1.00, *I*^2^ = 0%), and the fixed-effects model was performed by the *M-H* test.

*(3) Stenocardia*. Twelve studies [34, 41, 46, 50, 51, 57, 89, 95, 96, 98–100] with 1,011 patients were treated after the PCI compared with the rest of four studies [39, 58, 67, 83] with 434 patients being treated before and after the PCI (Figure 5). The result showed that the occurrence of stenocardia for the experimental group was lower than the controlled group both on the two time points of intervention (after the PCI: RR = 0.49, 95% CI = 0.33 to 0.72, *P* = 0.0003; before and after the PCI: RR = 0.40, 95% CI = 0.18 to 0.89, *P* = 0.02; overall: RR = 0.47, 95% CI = 0.33 to 0.66, *P* < 0.0001). The TCMI with the effect of *Liqihuoxue* [34, 39, 50, 51, 57, 58, 67, 89, 95, 96, 98–100] showed the superiority on the time points before and after the PCI and after the PCI. No heterogeneity was found (after the PCI: *P* = 0.94, *I*^2^ = 0%; before and after the PCI: *P* = 0.61, *I*^2^ = 0%; overall: *P* = 0.97, *I*^2^ = 0%), and the fixed-effects model was performed by the *M-H* test.

*(4) Arrhythmia*.  Figure 6 illustrated the outcome of arrhythmia. Three studies [41, 46, 71] with 216 patients received the treatment after the PCI compared with the five studies [39, 42–44, 93] with 567 patients received the treatment before and after the PCI. The result showed that the occurrence of arrhythmia for the experimental group was lower than the controlled group on the time points before and after the PCI (RR = 0.33, 95% CI = 0.2 to 0.56, *P* < 0.001). Both TCMI with the effect of *Liqihuoxue* [39, 42, 93] and *Yiqihuoxue* [41, 43, 44, 46, 71] showed the superiority on the intervention of time points before and after the PCI. No heterogeneity was found (after the PCI: *P* = 0.73, *I*^2^ = 0%; before and after the PCI: *P* = 0.95, *I*^2^ = 0%; overall: *P* = 0.99, *I*^2^ = 0%), and the fixed-effects model was performed by the *M-H* test.

In a word, even though the TCMI combined with western medicine showed the advantage on some indicators of the MACE compared with western medicine alone , the result still could not recommend the best applying point of TCMI during the perioperative period of PCI for patients with ACS.

#### 3.4.3. Inflammatory Factors

**Figure 7:**
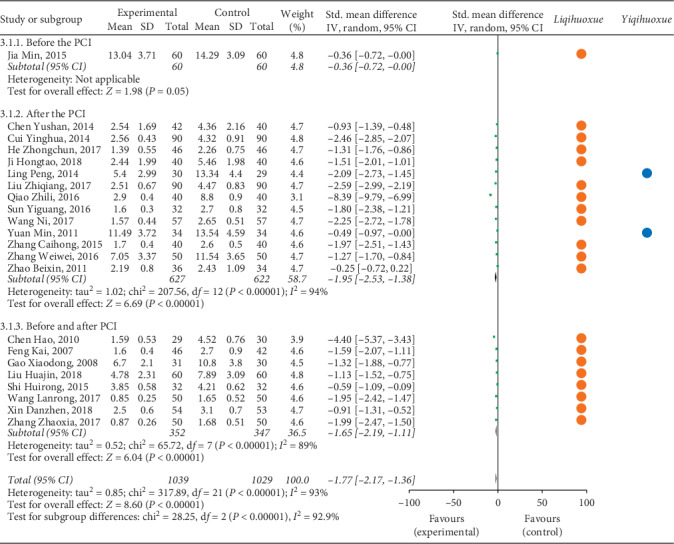
Forest plot of hs-CRP based on the time point of intervention and the effect of *Liqihuoxue* or *Yiqihuoxue*.

**Figure 8:**
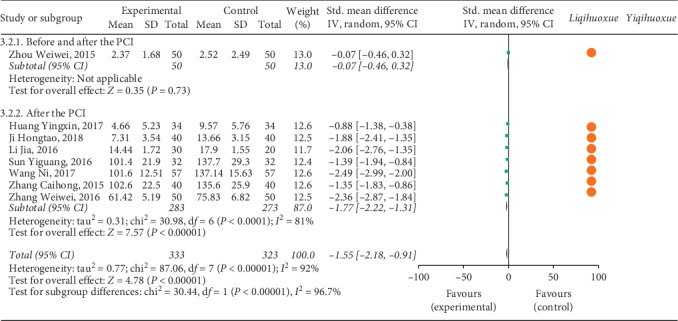
Forest plot of IL-6 based on the time point of intervention and the effect of *Liqihuoxue* or *Yiqihuoxue*.

Figures 7 and 8 illustrate the inflammatory factors (hs-CRP and IL-6) of patients with ACS after the treatment of experimental group and controlled group based on the effect of *Yiqihuoxue* or *Liqihuoxue* and the time points of intervention.

*(1) hs-CRP*. A total of 13 studies [34, 37, 45, 47, 52, 53, 59, 62, 94, 96, 98–100] with 1,249 patients were treated after the PCI compared with 8 studies [36, 39, 42, 63, 66, 67, 91, 93] with 699 patients being treated before and after the PCI (Figure 7). The result of meta-analysis indicated that the level of hs-CRP for the experimental group was lower than the controlled group (after the PCI: *Std*. MD = −1.95, 95% CI = −2.53 to −1.38, *P* < 0.001; before and after the PCI: *Std*. MD = −1.65, 95% CI = −2.19 to −1.11, *P* < 0.001; overall: *Std*. MD = −1.77, 95% CI = −2.17 to −1.36, *P* < 0.001). The TCMI with the effect of *Liqihuoxue* [34, 36, 37, 39, 42, 52, 53, 59, 62, 63, 66, 67, 91, 93, 94, 96, 98–100] was superior to the *Yiqihuoxue* [45, 47] during the perioperative period of PCI. But it still could not recommend the best time point of intervention during the perioperative period of PCI. Significant statistical heterogeneity was found (after the PCI: *P* < 0.01, *I*^2^ = 99%; before and after the PCI: *P* < 0.01, *I*^2^ = 97%; overall: *P* < 0.01, *I*^2^ = 98%), and the random-effects model was performed by the *IV* test. The subgroup analysis was applied to explore the source of heterogeneity based on the classification of area (north or south of China), level of hospitals (three A hospital or not), and sample size of studies (more than 100 or less than 100). The result indicated that the level of hospitals might was the source of heterogeneity (see Figures S3–S5 in the Supplementary Materials).

*(2) IL-6*. Seven articles [34, 35, 53, 97–100] with 556 patients received the treatment after the PCI compared with only 1 article [73] with 100 patients received the treatment before and after the PCI (Figure 8). The result showed that the IL-6 for the experimental group was lower than the controlled group on the time point after the PCI (*Std*. MD = −1.77, 95% CI = −2.22 to −1.31, *P* < 0.001), and the *Liqihuoxue* [34, 35, 53, 73, 97–100] was the most frequent effect of TCMI in this part. Obvious heterogeneity was found (after the PCI: *P* < 0.01, *I*^2^ = 81%; overall: *P* < 0.01, *I*^2^ = 92%), and the random-effects model was performed by the *IV* test. The subgroup analysis was also conducted to explore the source of heterogeneity based on the classification of area (north or south of China), level of hospitals (three A hospital or not), and sample size of studies (more than 100 or less than 100). But the result could not reveal the source of heterogeneity (see Figures S6–S8 in the Supplementary Materials).

### 3.5. Adverse Events

From the included researches, the report of potential adverse events mainly concentrated on bleeding events [37, 46, 58, 60, 62, 95], kidney disfunction [41, 51], angina pectoris or myocardial infarction [41–43, 91, 92], arrhythmia [41–43, 46], respiratory system disfunction [41, 92], heart failure [46, 91], allergy [51, 57, 62], headache [57], digestive system disfunction [92], and dizziness [91, 92]. Although there was no evidence that adverse events were directly caused by the application of TCMI, the bleeding events including gastrointestinal and gingival bleeding, haemoptysis, puncture point hematoma, and subcutaneous congestion were the most relevant events.

### 3.6. Publication Bias

**Figure 9:**
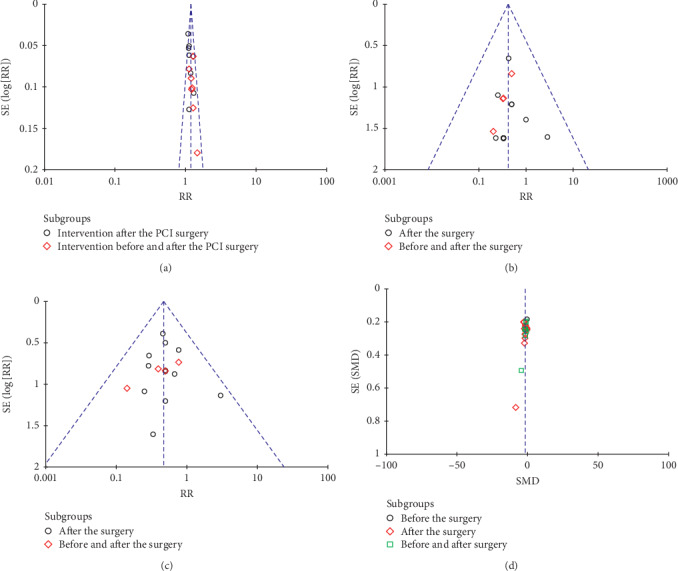
The funnel plot of (a) clinical efficiency, (b) MI, (c) stenocardia, and (d) hs-CRP.

We applied the RR or MD as the midpoint to draw the funnel plot (Figure 9). The publication bias was evaluated in the funnel plot by comparing the symmetry of included studies on clinical efficiency, MI, stenocardia, and hs-CRP. Each outcome indicator should include more than 10 studies. The funnel plot was symmetrical in visual for clinical efficiency, MI, and stenocardia, while not for hs-CRP. The statistical method of *Egger's* and *Begg's* test was conducted and further verified the publication bias by the software *Stata*. The results of *Egger's* and *Begg's* test indicated that the publication bias did not exist in clinical efficiency (*Egger's* test (*t* = 0.05, *P*=0.962 > 0.05); *Begg's* test (*z* = 0.25, *P*=0.805 > 0.05)) and hs-CRP (*Egger's* test (*t* = −0.89, *P*=0.389 > 0.05); *Begg's* test (*z* = 1.86, *P*=0.063 > 0.05)). However, the MI (*Egger's* test (*t* = −5.73, *P*=0.001); *Begg's* test (*z* = 2.60, *P*=0.009)) and stenocardia (*Egger's* test (*t* = −4.08, *P*=0.001); *Begg's* test (*z* = 2.28, *P*=0.023)) obtained the publication bias (see Figures S9–S12 in the Supplementary Materials).

## 4. Discussion

As one of the diseases that endanger human health and life seriously, ACS has aroused extensive attention all over the world [5]. The PCI has been widely applied in the treatment of ACS, and the prognosis has dramatically improved [18]. However, some PCI-related problems, such as no-reflow, ischemia-reperfusion injury, PMI, in-stent restenosis, and stent thrombosis, are difficult to avoid. Previous research studies illustrated that TCMI had a good effect on preventing arrhythmia and reperfusion injury, improving heart function, and protecting myocardium [22]. However, there was insufficient medical evidence for the TCMI in patients with ACS based on the effective classification of *Liqihuoxue* and *Yiqihuoxue*. This study was based on the PRISMA statement, focusing on the efficacy and safety of TCMI for ACS with the effect of *Yiqihuoxue* or *Liqihuoxue* and the time points of intervention during the perioperative period of PCI. The characteristics of TCMI and the precision of intervention are well illustrated.

A total of 68 articles with 6,043 patients were enrolled in this meta-analysis. The result of meta-analysis showed that the clinical efficiency of TCMI combined with western medicine (experimental group) was superior to the western medicine alone (controlled group) on patients with ACS during the perioperative period of PCI (before the PCI, before and after the PCI, or both), and the TCMI with the effect of *Liqihuoxue* was the relatively better choice. The result of MACE illustrated that the occurrence of MI, stenocardia, and arrhythmia for the experimental group was lower than the controlled group (MI and stenocardia: time points before the PCI, before and after the PCI, or both; arrhythmia: time points before and after PCI). However, the occurrence of all-cause mortality did not prove the advantage of TCMI. The TCMI with the effect of *Liqihuoxue* was the relatively better choice for the prevention of MACE based on the evaluation of classification. The result of meta-analysis for inflammatory factors showed that the level of hs-CRP and IL-6 for the experimental group was lower than the controlled group (hs-CRP: in the period of before the PCI, before and after the PCI, or both; IL-6: after the PCI) and both TCMI with the effect of *Liqihuoxue* and *Yiqihuoxue* has shown the superiority. The heterogeneity of some indicators (hs-CRP and IL-6) was extremely obvious, and the result of subgroup analysis indicated the level of hospitals might be the source of heterogeneity for hs-CRP. After each included study was excluded individually based on the procedure of sensitivity analysis, the majority of the combined effects were relatively close and stable.

The publication bias existed in this research after *Egger's* and *Begg's* tests. It might come from the following reasons: (a) some authors tended to deliver positive results to editors while prejudiced negative results [102]; (b) some editors or reviewers had a preference to positive results while cavilled to negative results to some extent [103]; (c) government funding researches had more possibilities to be published in some magazines than receiving private or company funding [104]. The meta-analysis would overstate the degree of association between treating effects and risk factors because of the publication bias, bringing mistakes for clinical therapy or health decision-making.

Numerous previous systematic reviews and meta-analyses have been published to confirm the clinical efficacy and safety of TCM for the treatment of CHD. However, there still remained some problems. Firstly, some of them only focused on the broad category of CHD without evaluating the specific type of disease, leading to the restriction of clinical application [105, 106]. Secondly, some of them did not classify the category and dosage of TCM, leading to more confounding factors and high risk of bias [107]. Thirdly, some studies did not highlight the precise time point of intervention for TCMI during the perioperative period of PCI [108, 109]. Compared with previous research studies, the characteristics of our research were clearly classification of TCMI (the effect of *Yiqihuoxue* and *Liqihuoxue*), accurate selection of disease types from the CHD, and precise time point of intervention during the perioperative period of PCI (before the PCI, before and after the PCI, after the PCI, and overall).

It should be noted that some limitations did exist as follows. Firstly, all included studies were conducted in different hospitals in China, which might bring the regional and cultural bias based on the different clinical abilities of ACS diagnosis and PCI treatment. Secondly, the included RCTs had flaws caused by human baseline risk factors (all patients were Chinese), incomplete methodological design of trials (lack of blinding method), and small sample size (less than 30 patients per group). Thirdly, some results showed significant heterogeneity, which might be due to the sample size, the different experimental regions in China, medicine application and dose, publication years, and the duration of treatment. The lower quality of included RCTs restricted the promotion of evidence. Fourthly, the random-effects model was established to pool data, which might not provide the exact and stable conclusion based on this situation.

The report of adverse events of TCM, including the TCMI, has always been a hotspot issue in clinical practice. Recently published retrospective research, which reviewed the data from 10,000 heart failure patients, found that Salvia miltiorrhiza/*Danshen* might increase the risk of bleeding and death [110]. Some articles emphasized that the occurrence of adverse events was actually related to the nonstandardized use of Chinese medicine in western medical hospitals so that the clinical value of TCM should not be negated completely. The precise treatment and safety evaluation of TCM are essential for the development of TCM, and this meta-analysis could provide evidence-based support and guidance.

## 5. Conclusions

Our research provides a beneficial and promising result for the application of TCMI (*Liqihuoxue* or *Yiqihuoxue*) combined with western medicine on patients with ACS during the perioperative period of PCI. This combined therapy can provide assistance for improving clinical efficiency, reducing the incidence rate of MACE, and lowering the level of inflammatory factors. We did not find the optimal time point of intervention during the perioperative period of PCI. Although the application of TCMI with the effect of *Liqihuoxue* obtained support from this research, the effect of *Liqihuoxue* or *Yiqihuoxue* for TCMI still needs more evidence from the standard, multicentre, double-blind RCTs in the future. The precise application of TCMI during the perioperative period of PCI will be one of the new directions for TCM in the future.

## Figures and Tables

**Table 1:** The characteristics of included studies.

Article	Area	Classification of disease	Sample size (male/female)	Age (years, average age: mean ± SD or mean)	Other information of baseline characteristics	Experimental group (E)	Controlled group (C)	Duration of treatment and follow-up	Outcome evaluation and quality assessment	Adverse event
(1) Hongtao and Yuan [34]	Henan Province; China; *multicenters*	AMI	80 (48/32)	E: 43 to 61; 51.4 ± 5.1 C: 42 to 59; 49. 3 ± 4.6	NYHA: E/C: I: 13/14, II: 12/13, III: 8/8, IV: 7/5	Injection of *Dazhuhongjingtian* combined with ①, ②, and ③ treatment (*n* = 40, after the PCI)	①, ②, and ③ treatment (*n* = 40, after the PCI)	Four weeks; Six months	(1) Clinical efficiency	NR
									(2) Indexes of inflammatory cytokines (MPO, hs-CRP, IL-6, and TNF-*α*)	
									(3) Color Doppler ultrasound (LVEDD and LVESD)	
									(4) Indexes of markers of myocardial injury (BNP, cTnT, and CK-MB)	
									(5) MACE	

(2) Jia and Jun [35]	Jiangsu Province; China; single center	ACS	80 (48/32)	40 to 83; 63.11	NR	Injection of *Dazhuhongjingtian* combined with ①, ②, and ③ treatment (*n* = 30, after the PCI)	①, ②, and ③ treatment (*n* = 30, after the PCI)	Three to seven days; NR	(1) Clinical efficiency	NR
									(2) Laboratory indexes (CK-MB, LDH, and AST)	
									(3) Indexes of inflammatory cytokines (IL-6, TNF-*α*, SOD, NO, and CRP)	

(3) Huirong et al. [36]	Hebei Province; China; single center	UA	64 (31/33)	E: 50 to 72; 60.39 ± 7.79 C: 51 to 70; 58.9 ± 7.45	BMI: E/C: (25.87 ± 3.29)/(26.62 ± 3.16)	Injection of *Dazhuhongjingtian* combined with ①, ②, ③, and ④ treatment (*n* = 32, after the PCI)	①, ②, ③, and ④ treatment (*n* = 32, after the PCI)	Fourteen days; NR	Laboratory indexes (MCP-1 and hs-CRP)	NR

(4) Yushan et al. [37]	Henan Province; China; single center	AMI	82 (52/30)	31 to 72; 51.3 ± 27.3	NR	Injection of *Dazhuhongjingtian* combined with ①, ②, ③, and ④ treatment (*n* = 42, after the PCI)	①, ②, ③, and ④ treatment (*n* = 40, after the PCI)	Fourteen days; NR	Laboratory indexes (ET, hs-CRP, Fb, and blood lipid)	I

(5) Xin [38]	Jiangsu Province; China; single center	ACS	40 (30/10)	E: 61.05 ± 9.62 C: 63.35 ± 10.67	NR	Injection of *Dazhuhongjingtian* combined with ①, ②, ③, and ④ treatment (*n* = 20, after the PCI)	①, ②, ③, and ④ treatment (*n* = 20, after the PCI)	Five to seven days; NR	(1) Indexes of markers of myocardial injury (CK-MB, LDH, and cTnT)	NR
									(2) Blood biochemical examination	
									(3) Indexes of inflammatory cytokines (IL-6, SOD, and CRP)	

(6) Lanrong [39]	Hebei Province; China; single center	AMI	100 (58/42)	E: 50 to 72 C: 50 to 75	NR	Injection of *Shuxuetong* and *Shenmai* combined with ①, ②, and ③ treatment (*n* = 50, before and after the PCI)	①, ②, and ③ treatment (*n* = 50, before and after the PCI)	One week; NR	(1) hs-CRP	NR
									(2) Color Doppler ultrasound (LA, LVEDD, LVESD, and VEF%)	
									(3) MACE	

(7) Fengmei et al. [40]	Zhejiang Province; China; single center	AMI	67 (52/15)	E: 65.9 ± 10.4 C: 66.2 ± 11.1	Combined diseases: E/C: hypertension: 22/23; diabetes: 17/13; hyperlipidemia: 5/7	Injection of *Shenmai* combined with ①, ②, ③, and ④ treatment (*n* = 35, before and after the PCI)	①, ②, ③, and ④ treatment (*n* = 32, before and after the PCI)	Seven days; NR	(1) Clinical efficiency	NR
									(2) Laboratory indexes (apelin-13 and NO)	

(8) Lin et al. [41]	Liaoning Province; China; single center	ACS	74 (35/39)	35 to 84; 59.22 ± 7.03	NR	Injection of *Shenmai* combined with ② and ③ treatment (*n* = 37, after the PCI)	② and ③ treatment (*n* = 37, after the PCI)	Eight weeks; NR	(1) Clinical efficiency	I; II; III; IV; IX
									(2) Laboratory index	
									(3) ECG	
									(4) Adverse events	

(9) Zhaoxia [42]	Hebei Province; China; single center	STEMI	100 (55/45)	E: 69. 0 ± 7.6 C: 68.2 ± 7.1	HYHA: E/C: I: 22/24; II: 28/26 Site of MI: E/C: anterior wall and extensive anterior wall: 28/26; inferior wall: 14/15; high lateral: 8/9.	Injection of *Shuxuetong* and *Shenmai* combined with ②, ③, and ⑤ treatment (*n* = 50, before and after the PCI)	②, ③, and ⑤ treatment (*n* = 50, before and after the PCI)	One week; NR	(1) Laboratory indexes (hs-CRP, SOD, and MDA)	III; IV
									(2) Color Doppler ultrasound (LVEF and size of MI)	
									(3) MACE	

(10) Lilan and Xiaoxiao [43]	Zhejiang Province; China; single center	AMI	100 (61/39)	E: 45 to 78, 58.41 ± 12.39 C: 43 to 78, 57.68 ± 12.03	NR	Injection of *Shenmai* combined with ②, ③, ④, and ⑤ treatment (*n* = 50, before and after the PCI)	②, ③, ④, and ⑤ treatment (*n* = 50, before and after the PCI)	Seven days; one to six months	(1) Color Doppler ultrasound	III; IV
									(2) Indexes of markers of myocardial injury (CK-MB, BNP, and cTnT)	
									(3) Adverse events	

(11) Hua et al. [44]	Anhui Province; China; single center	AMI	92 (58/34)	E: 62.72 ± 12.12 C: 61.27 ± 10.84	Combined diseases: E/C: hypertension: 19/21; diabetes: 12/13; smoke: 17/21; alcohol consumption: 14/12.	Injection of *Shenmai* combined with ②, ③, and ④ treatment (*n* = 46, before and after the PCI)	②, ③, and ④ treatment (*n* = 46, before and after the PCI)	Seven days; three months	(1) Blood biochemical examination	NR
									(2) Color Doppler ultrasound	
									(3) MACE	

(12) Peng et al. [45]	Jiangsu Province; China; single center	STEMI	120 (104/16)	E1: 47 to 75, 61.2 ± 9.8 E2: 45 to 75, 61.9 ± 10.1 E3: 48 to 75, 59.7 ± 8.5 C: 47 to 75, 59.7 ± 8.1	NR	E1: *Salvianolate* injection combined with ①, ②, ③, ④, and ⑤ treatment (*n* = 30, after the PCI) E2: *Shenmai* injection combined with ①, ②, ③, ④, and ⑤ treatment (*n* = 30, after the PCI) E3: *Salvianolate* injection and *Shenmai* injection combined with ①, ②, ③, ④, and ⑤ treatment (*n* = 30, after the PCI)	①, ②, ③, ④, and ⑤ treatment (*n* = 30, after the PCI)	Seven days; NR	(1) LVEF	NR
									(2) Nt-proBNP	
									(3) hs-CRP	
									(4) Adverse events	

(13) Caiyan et al. [46]	Zhejiang Province; China; single center	STEMI	38 (23/15)	43 to 77, 63.83 ± 8.3	NR	*Shenmai* injection combined with ①, ②, ③, ④, and ⑤ treatment (*n* = 19, after the PCI)	①, ②, ③, ④, and ⑤ treatment (*n* = 19, after the PCI)	Two weeks; twenty-two weeks	(1) Plasma aldosterone	III; IV; V
									(2) Color Doppler ultrasound	
									(3) Adverse events	
(14) Min et al. [47]	Zhejiang Province; China; single center	AMI	68 (NR)	NR	NR	*Shenmai* injection combined with conventional western medicine (NR) (*n* = 34, after the PCI)	Conventional western medicine (NR) (*n* = 34, after the PCI)	One week; NR	Indexes of inflammatory cytokines (NO, ET, SOD, hs-CRP, CD62P, and CD63)	NR

(15) Rong et al. [48]	Liaoning Province; China; multicenters	AMI	56 (35/21)	E: 47 to 68, 56.7 ± 10.2 C: 46 to 67, 55.9 ± 11	NR	*Shenmai* injection combined with ①, ②, ③, ④, and ⑤ treatment (*n* = 30, before and after the PCI)	①, ②, ③, ④, and ⑤ treatment (*n* = 26, before and after the PCI)	Two weeks; four weeks	(1) Color Doppler ultrasound	NR
									(2) Clinical events	

(16) Faming et al. [49]	Shandong Province; China; multicenters	STEMI	98 (65/33)	E: 64.28 ± 12.28 C: 63.96 ± 12.25	Killip classification: E/C: I: 38/39, II: 5/4, III: 1/2, and IV: 1.	*Compound Salvia miltiorrhiza* injection combined with ①, ②, ③, and ⑤ treatment (*n* = 49, before and after PCI)	①, ②, ③, and ⑤ treatment (*n* = 49, before and after PCI)	17 days; NR	MACE	NR

(17) Yonghao et al. [50]	Guangdong Province; China; single center	ACS	60 (34/26)	E: 30 to 78, 49.45 ± 11.03 C: 30 to 76, 48.63 ± 10.49	Combined diseases and personal history: E/C: diabetes: 8/7; hypertension: 6/7; smoke: 13/11; hyperlipidemia: 3/5	*Danhong* injection combined with ①, ②, ③, and ⑤ treatment (*n* = 30, after the PCI)	①, ②, ③, and ⑤ treatment (*n* = 30, after the PCI)	Two weeks; NR	(1) Indexes of markers of myocardial injury	NR
									(2) Color Doppler ultrasound (LVEF and LVED)	
									(3) MACE	

(18) Guangwei et al. [51]	Shaanxi Province; China; multicenters	STEMI	120 (74/46)	E: 58 to 80, 65.13 ± 2.38 C: 56 to 78, 64.38 ± 2.12	NR	*Danhong* injection combined with ①, ②, ③, and ⑤ treatment (*n* = 60, after the PCI)	①, ②, ③, and ⑤ treatment (*n* = 60, after the PCI)	Fourteen days; six months	(1) Clinical efficiency	II; VI
									(2) Indexes of IL-6 and IL-17	
									(3) LVEF	
									(4) MACE	
									(5) Adverse events	

(19) Zhiqiang et al. [52]	Henan Province; China; single center	NSTEMI	180 (NR)	NR	NR	*Danhong* injection combined with ② treatment (*n* = 90, after the PCI)	② treatment (*n* = 90, after the PCI)	14 days; NR	(1) Indexes of hs-CRP and ET	NR
									(2) Color Doppler ultrasound (cardiac function)	
									(3) Clinical efficiency	

(20) Weiwei et al. [53]	Shandong Province; China; single center	ACS	100 (67/33)	E: 61 to 80, 71.26 ± 4.82 C: 61 to 79, 68.28 ± 4.88	NR	*Danhong* injection combined with ②, ③, and ⑤ treatment (*n* = 50, after the PCI)	②, ③, and ⑤ treatment (*n* = 50, after the PCI)	Two weeks; Two months	(1) Vascular endothelial function	NR
									(2) Indexes of inflammatory cytokines (IL-6, MMP-9, and hs-CRP)	

(21) Mengzhao [54]	Guangxi Province; China; single center	AMI	100 (63/37)	35 to 70, 52.87 ± 9.03	NR	*Danhong* injection combined with ⑥ (*n* = 52, after PCI)	⑥ (*n* = 52, after PCI)	Three days after the PCI; NR	(1) Indexes of inflammatory cytokines (hs-CRP, and IL-10)	NR
									(2) Laboratory indexes (MMP-9 and BNP)	
									(3) Color Doppler ultrasound	

(22) Yang [55]	Hebei Province; China; multicenters	ACS	104 (55/49)	E: 47 to 74, 58.73 ± 8.45 C: 48 to 72, 59.21 ± 8.57	NR	*Danhong* injection combined with ② and ③ treatment (*n* = 52, after the PCI)	② and ③ treatment (*n* = 52, after the PCI)	Two weeks; NR	(1) Vascular endothelial function	NR
									(2) Indexes of inflammatory cytokines (pentraxin-3, IL-18, IL-10, and LpPLA2)	
									(3) Color Doppler ultrasound	

(23) Min et al. [56]	Hebei Province; China; single center	AMI	120 (75/45)	E: 51 to 74, 62.23 ± 11.26 C: 51 to 77, 64.56 ± 12.85	NR	*Danhong* injection combined with ② treatment (*n* = 60, after the PCI)	② treatment (*n* = 60, after the PCI)	NR	(1) CRP	NR
									(2) Rate of no-reflow	

(24) Xinmin et al. [57]	Shanghai city; China; single center	AMI	71 (49/22)	48 to 81, 64 ± 12	NR	*Danhong* injection combined with conventional western medical treatment (*n* = 36, after the PCI)	Conventional western medical treatment (*n* = 35, after the PCI)	Fourteen days; NR	(1) Clinical efficiency	VII; VI
									(2) MACE	
									(3) LVEF	
									(4) Adverse events	

(25) Jianfeng et al. [58]	Zhejiang Province; China; multicenters	ACS	125 (69/56)	E: 55 to 79, 62.1 ± 10.6 C: 53 to 76, 61.5 ± 10.3	Classification of ACSE/C: AMI: 36/35; UA: 27/27.	*Danhong* injection combined with ②, ③, and ⑤ treatment (*n* = 63, before and after the PCI surgery)	②, ③, and ⑤ treatment (*n* = 62, before and after the PCI surgery)	Two weeks; Two months	(1) Vascular endothelial function	NR
									(2) Indexes of inflammatory cytokines (TNF-*α*, IL-1, and CRP)	
									(3) MACE	

(26) Yinghua and Lin [59]	Tianjing city; China; multicenters	AMI	180 (106/47)	E: 57 to 79, 72.1 ± 6.5 C: 55 to 80, 72.3 ± 5.8	NR	*Danhong* injection combined with ②, ③, and ⑤ treatment (*n* = 90, after the PCI)	②, ③, and ⑤ treatment (*n* = 90, after the PCI)	Ten days; NR	(1) Clinical efficiency	I
									(2) Level of SOD and hs-CRP	
									(3) Adverse events	

(27) Yongxiang and Qiang [60]	Henan Province; China; single center	ACS	68 (41/27)	E: 55.7 ± 7.4 C: 54.5 ± 8.2	BMI: E/C: (20.6 ± 2.1)/(21.5 ± 1.6)	*Danhong* injection combined with ②, ③, and ⑤ treatment (*n* = 34, after the PCI)	②, ③, and ⑤ treatment (*n* = 34, after the PCI)	10 days; NR	(1) Falling rate of ST-segment	I
									(2) Adverse events	

(28) Xiaonan et al. [61]	Tianjing city; China; multicenters	AMI	60 (39/21)	NR	NR	*Danhong* injection combined with ② and ③ treatment (*n* = 30, before and after the PCI)	② and ③ treatment (*n* = 30, before and after the PCI)	Two weeks; NR	(1) Cardiac arrhythmia before and after the PCI	NR
									(2) CK-MB	
									(3) Scattering parameters	

(29) Beixin and Shan [62]	Liaoning Province; China; single center	ACS	70 (37/33)	33 to 75, 54.5 ± 10.9	NR	*Danhong* injection combined with ② and ③ treatment (*n* = 36, after the PCI)	② and ③ treatment (*n* = 36, after the PCI)	Two weeks; NR	(1) hs-CRP and ET-1	I; VI
									(2) Adverse events	

(30) Hong et al. [63]	Hebei Province; China; single center	AMI	59 (43/16)	E: 55 to 71, 61.9 ± 5.2 C: 54 to 71, 65.2 ± 4.5	Combined diseases: E/C: hypertension: 28/29; diabetes: 16/23; hyperlipidemia: 26/24.	*Danhong* injection combined with ② and ③ treatment (*n* = 29, before and after the PCI)	② and ③ treatment (*n* = 30, before and after the PCI)	Fourteen days; NR	(1) hs-CRP	NR
									(2) Falling rate of ST-segment	

(31) Yong et al. [64]	Hunan Province; China; single center	ACS	42 (27/15)	E: 70.6 ± 5.4 C: 69.1 ± 6.0	Classification of ACS: E/C: AMI: 6/5; UA: 15/16	*Danhong* injection combined with ② and ③ treatment (*n* = 21, after the PCI)	② and ③ treatment (*n* = 21, after the PCI)	Fourteen days; NR	Indexes of platelet activation (CD62P and CD63)	NR

(32) Zhihui et al. [65]	Jilin Province; China; single center	AMI	203 (111/92)	E: 39 to 79, 71.6 ± 8.6 C: 49 to 75, 70.1 ± 8.1	Combined diseases: E/C: AMI: 31/26; diabetes: 30/29; hypertension: 35/27	*Danhong* injection combined with ①, ②, and ③ treatment (*n* = 116, after the PCI)	①, ②, and ③ treatment (*n* = 87, after the PCI)	Fourteen days; NR	(1) Clinical efficiency	NR
									(2) Indexes of coagulation function	
									(3) Color Doppler ultrasound	
									(4) TIMI	

(33) Xiaodong et al. [66]	Beijing city; China; multicenters	STEMI	61 (38/23)	E: 60.1 ± 10.6 C: 59.8 ± 7.6	NR	*Danhong* injection combined with ①, ②, and ③ treatment (*n* = 31, before and after the PCI)	①, ②, and ③ treatment (*n* = 30, before and after the PCI)	Fourteen days; NR	(1) ECG	NR
									(2) Symptom of MI	
									(3) CRP	

(34) Kai et al. [67]	Shanghai city; China; single center	ACS	91 (66/25)	E: 65.6 ± 17.3 C: 67.2 ± 16.2	Classification of ACS: E/C: UA: 23/23; STEMI: 14/13; NSTEMI: 8/10.	*Danhong* injection combined with ①, ②, and ③ treatment (*n* = 46, before and after the PCI)	①, ②, and ③ treatment (*n* = 45, before and after the PCI)	Four weeks; NR	(1) Lipid levels	NR
									(2) hs-CRP	
									(3) MACE	

(35) Fan and Shayi [68]	Guangxi Province; China; single center	ACS	67 (NR)	NR	Combined diseases: E/C: hypertension: 25/21; hyperlipidemia: 19/16; diabetes: 10/8	*Dengzhanhuasu* injection combined with ①, ④, and ⑤ treatment (*n* = 37, before and after the PCI)	①, ④, and ⑤ treatment (*n* = 30, before and after the PCI)	One week; NR	(1) Hemorrheology	NR
									(2) Braunwald classification of angina pectoris	
									(3) MACE	

(36) Yuting and Zheng [69]	Neimenggu Province; China; single center	ACS	56 (NR)	E: 67.8 ± 9.3 C: 65.6 ± 0.1	Combined diseases and personal history: E/C: smoke: 64.2%/21.4%; diabetes: 21.4%/25%	*Gualoupi* injection combined with ②, ③, and ④ treatment (*n* = 28 after the PCI)	②, ③, and ④ treatment (*n* = 28 after the PCI)	Fourteen days; NR	(1) Vascular endothelial function	NR
									(2) Platelet function	

(37) Hong et al. [70]	Hebei Province; China; single center	STEMI	98 (52/46)	E: 35 to 71, 55 ± 4 C: 34 to 71, 56 ± 5	Killip classification: E/C: I: 44/45; II: 4/5	*Guanxinning* injection combined with ②, ③, and ④ treatment (*n* = 48 after the PCI surgery)	②, ③, and ④ treatment (*n* = 50 after the PCI surgery)	Ten days; NR	(1) Color Doppler ultrasound	NR

(38) Hong et al. [71]	Hebei Province; China; single center	STEMI	86 (56/30)	34 to 72	NR	*Guanxinning* injection combined with ②, ④, and ⑤ treatment (*n* = 42 after the PCI)	②, ④, and ⑤ treatment (*n* = 44 after the PCI)	NR; Three months	(1) LVEF	NR
									(2) MACE	
(39) Rui et al. [72]	Shaanxi Province; China; single center	UA	60 (41/19)	E: 63.5 ± 11.2 C: 61.3 ± 13.7	Combined diseases and personal history: E/C: hypertension: 11/9; diabetes: 9/12; smoke: 17/13	*Safflower yellow* injection combined with ②, ④, and ⑤ treatment (*n* = 30 before the PCI)	②, ④, and ⑤ treatment (*n* = 30 before the PCI)	NR	(1) Myocardial injury markers	NR

(40) Weiwei et al. [73]	Beijing city; China; single center	UA	100 (70/30)	42 to 77, 58 ± 9.2	NR	*Danhong* injection combined with ①, ②, and ③ treatment (*n* = 50, before and after the PCI)	①, ②, and ③ treatment (*n* = 50, before and after the PCI)	Seven days; NR	(1) Clinical efficiency	NR
									(2) Laboratory indexes (IL-6, cTNT, and hs-CRP)	

(41) Chuntao and Lihua [74]	Shaanxi Province; China; single center	UA	180 (102/78)	E: 45 to 76, 62.38 ± 7.14 C: 46 to 78, 62.53 ± 7.48	Combined diseases: E/C: hypertension: 40/47; hyperlipidemia: 28/28; diabetes: 22/13	*Danhong* injection combined with ①, ②, and ③ treatment (*n* = 90, before and after the PCI)	①, ②, and ③ treatment (*n* = 90, before and after the PCI)	Two weeks; NR	(1) Clinical efficiency	NR
									(2) Vascular endothelial function (NO, ET-1, vWF, and FMD)	

(42) Yunshu et al. [75]	Jilin Province; China; single center	NSTEMI	100 (61/39)	More than 65 years old	NR	*Safflower yellow* injection combined with ①, ②, ③, and ④ treatment (*n* = 50, before and after the PCI)	①, ②, ③, and ④ treatment (*n* = 50, before and after the PCI)	Ten to fourteen days; thirty days	(1) Clinical efficiency	NR
									(2) Laboratory indexes	
									(3) Adverse events	
									(4) Bleeding events	

(43) Dingxue and Wenbao [76]	Shaanxi province; China; single center	ACS	88 (33/53)	44 to 85, 68.1 ± 8.5	The area of infraction: anterior wall: infarction: 6, extensive anterior wall infarction: 24; lateral wall infarction: 28; inferior and posterior wall infarction: 20	*Safflower* injection combined with ② and ③ treatment (*n* = 44, after the PCI)	② and ③ treatment (*n* = 44, after the PCI)	Fourteen days; Four weeks	(1) Color Doppler ultrasound	NR
									(2) MACE	

(44) Xian et al. [77]	Shanghai city; China; single center	ACS	88 (51/37)	E: 45 to 83, 63.5; C: 51 to 82, 64.5	Classification: E/C: UA: 30/28; NSTEMI: 14/12	*Safflower* injection combined with ② and ③ treatment (*n* = 44, before and after the PCI)	② and ③ treatment (*n* = 44, before and after the PCI)	Seven days; NR	(1) Clinical efficiency	NR
									(2) Laboratory indexes (CRP and TnI)	

(45) Suyun et al. [78]	Hebei Province; China; single center	UA	102 (62/40)	E: 54.4 ± 8.6 C: 56.6 ± 7.4	NR	*Safflower* injection combined with ②, ③, and ⑤ treatment (*n* = 51, before the PCI)	②, ③, and ⑤ treatment (*n* = 51, before the PCI)	NR	(1) ECG (ST-segment)	NR
									(2) Vascular endothelial function (NO and ET-1)	
									(3) Indexes of inflammatory cytokines (IL-1*β*, IL-6, and TNF-*α*)	

(46) Yujuan and Maiti [79]	Xinjiang Province; China; single center	AMI	124 (73/51)	E: 58.4 ± 9.6 C:57.6 ± 10.1	Infarction relate artery: E/C: center anterior descending branch: 32/30; center circumflex branch: 10/11; right coronary artery: 20/21.	*Kudiezi* injection combined with ②, ③, and ⑤ treatment (*n* = 62, before and after the PCI)	②, ③, and ⑤ treatment (*n* = 62, before and after the PCI)	Two weeks Six months	(1) ECG	NR
									(2) MACE	
									(3) Laboratory indexes (CK-MB, cTnI, and ET-1)	

(47) Yuefan et al. [80]	Shandong Province; China; single center	UA	81 (NR)	E: 68.7 ± 10 C: 68.1 ± 9.1	Personal history and combined diseases: E/C: smoke: 24/23; hypertension: 29/30; diabetes: 8/7	*Shengmai* injection combined with ②, ③, and ⑤ treatment (*n* = 41, after the PCI)	②, ③, and ⑤ treatment (*n* = 41, after the PCI)	NR	(1) Indexes of inflammatory cytokines (hs-CRP and TNF-*α*)	NR

(48) Yinghui [81]	Sichuan Province; China; single center	ACS	120 (67/53)	E: 34 to 65, 41 ± 1.2 C: 35 to 63, 42 ± 1.4	Combined diseases: E/C: hypertension: 58.33%/61.67%, diabetes: 33.3%/31.67%; family history of coronary heart disease: 6.67%/8.33%	*Shengmai* injection combined with ②, ③, and ④ treatment (*n* = 60, after the PCI)	②, ③, and ④ treatment (*n* = 60, after the PCI)	NR	(1) Blood lipid level	NR
									(2) The score of PL, AS, and AF	
									(3) The score SL and LP	
									(4) Color Doppler ultrasound	
									(5) Blood platelets	

(49) Xuan et al. [82]	Beijing city; China; multicenters	AMI	62 (35/27)	E: 36 to 89, 58 ± 14.9 C: 43 to 85, 54.9 ± 15.2	Combined diseases: E/C: hypertension: 24/22; diabetes: 10/7; dyslipidemia: 9/6; stroke: 3/3	*Shengmai* injection combined with ②, ③, ④, and ⑤ treatment (*n* = 32, before and after the PCI)	②, ③, ④, and ⑤ treatment (*n* = 30, before and after the PCI)	Seven days; NR	(1) TIMI	Nr
									(2) Color Doppler ultrasound	
									(3) Laboratory indexes	
									(4) MACE	

(50) Zhe et al. [83]	Shandong Province; China; single center	AMI	90 (49/41)	E: 61.1 ± 5.3; C: 61.0 ± 5.3	Combined diseases: E/C: hyperlipidemia: 17/15; hypertension: 22/23; diabetes: 6/7 NYHA: E/C: II: 30/32; III: 15/13.	*Shuxuetong* injection combined with ②, ③, and ⑤ treatment (*n* = 45, before and after the PCI)	②, ③, and ⑤ treatment (*n* = 45, before and after the PCI)	Ten days; NR	(1) Clinical efficiency	NR
									(2) Color Doppler ultrasound (LVMI, LVPWT, LVEDD, and LVEF)	
									(3) Laboratory indexes (CK-MB and cTnI)	
									(4) MACE	

(51) Xiaoyan [84]	Shaanxi Province; China; single center	AMI	60 (35/25)	E: 64 to 89, 73.5 ± 6.6) C: 63 to 88, 73.1 ± 6.5	Course of diseases: E/C: (4.3 ± 1.2)/(4.2 ± 1.1) years	*Shuxuetong* injection combined with ② and ③ treatment (*n* = 30, before and after the PCI)	② and ③ treatment (*n* = 30, before and after the PCI)	One week; NR	(1) Hemorheology	NR
									(2) Color Doppler ultrasound	
									(3). MACE	

(52) Zhenda et al. [85]	Guangdong Province; China; single center	AMI	40 (NR)	E: 64.2 ± 8.0 D: 63.5 ± 11.0	Combined diseases: E/C: diabetes: 24.2%/23.6%; hypertension: 72.7%/71.5%	*Shuxuetong* injection combined with ②, ③, ④, and ⑤ treatment (*n* = 20, after the PCI surgery)	②, ③, ④, and ⑤ treatment (*n* = 20, after the PCI surgery)	Two weeks; NR	(1) Color Doppler ultrasound	NR
									(2) Laboratory indexes	
									(3) Adverse events	
									(4) MACE	

(53) Xuguang and Rong [86]	Neimenggu Province; China; single center	UA	96 (52/44)	E: 42 to 72, 62.5 ± 10.1 C: 45 to 72, 61.6 ± 11.3	Course of disease: E/C: (7.2 ± 3.6)/(7.7 ± 3.8) years	*Shuxuetong* injection combined with ②, ③, ④, and ⑤ treatment (*n* = 60, before the PCI)	②, ③, ④, and ⑤ treatment (*n* = 60, before the PCI)	Fourteen days; NR	(1) Blood lipid level	NR
									(2) Coagulation function	
									(3) MACE	

(54) Tiezhou and Jie [87]	Jiangsu Province; China; single center	AMI	120 (82/38)	E: 40 to 84, 68.5 ± 8.5 C: 38 to 88, 67.5 ± 7.5	NR	*Shuxuetong* injection combined with ②, ③, ④, and ⑤ treatment (*n* = 60, after the PCI)	②, ③, ④, and ⑤ treatment (*n* = 60, after the PCI)	Two weeks; NR	(1) Clinical efficiency	NR
									(2) ECG	

(55) Yushuang et al. [88]	Jilin Province; China; multicenters	AMI	60 (31/29)	43 to 71, 57.8 ± 13.1	NR	*Shuxuetong* injection combined with ②, ③, ④, and ⑤ treatment (*n* = 30, before and after the PCI)	②, ③, ④, and ⑤ treatment (*n* = 30, before and after the PCI)	Three days; NR	(1) SICAM-1	NR

(56) Jingchun et al. [89]	Jiangxi Province; China; single center	ACS	84 (54/30)	E: 54 to 82, 58 ± 4 C: 52 to 78, 56 ± 4	NR	*Shuxuetong* injection combined with ②, ③, ④, and ⑤ treatment (*n* = 50, after the PCI)	②, ③, ④, and ⑤ treatment (*n* = 34, after the PCI)	One week; NR	(1) Vascular endothelial function	NR
									(2) MACE	
(57) Jianping et al. [90]	Guangdong Province; China; single center	AMI	60 (38/22)	E: 48 to 68, 53 C: 51 to 65, 59.3	Combined diseases: E/C: arrhythmia: 4/5; cardiogenic shock: 5/4; heart failure: 3/2.	*Xiangdan* injection combined with ②, ③, ④, and ⑤ treatment (*n* = 30, before and after the PCI)	②, ③, ④, and ⑤ treatment (*n* = 30, before and after the PCI)	Seven days; NR	Clinical efficiency	NR

(58) Huajin et al. [91]	Shanghai city; China; single center	STEMI	120 (73/47)	E: 40 to 72; C: 39 to 73	Combined diseases: E/C: diabetes: 14/15; hypertension: 25/26; hyperlipidemia: 21/19	*Xuesaitong* injection combined with ②, ③, ④, and ⑤ treatment (*n* = 60, before and after the PCI)	②, ③, ④, and ⑤ treatment (*n* = 60, before and after the PCI)	Two weeks; NR	(1) TIMI	IV; V; VIII
									(2) Indexes of inflammatory cytokines (hs-CRP and PTX-3)	
									(3) Color Doppler ultrasound	
									(4) Adverse events	

(59) Lianren [92]	Shandong Province; China; single center	STEMI	104 (59/45)	E: 23 to 78, 56.71 ± 6.25 C: 21 to 80, 57.29 ± 6.61	Combined diseases: E/C: diabetes: 13/11; hypertension: 29/30; hyperlipidemia: 22/23.	*Xuesaitong* injection combined with ②, ③, and ⑤ treatment (*n* = 52, before and after the PCI)	②, ③, and ⑤ treatment (*n* = 52, before and after the PCI)	Fourteen days; NR	(1) TIMI	I; IV; VIII; IX; IX
									(2) Color Doppler ultrasound	
									(3) Adverse events	

(60) Danzhen and Lingfei [93]	Zhejiang Province; China; single center	AMI	107 (64/43)	E: 51.9 ± 8.4 C: 52.3 ± 8.2	NR	*Xuesaitong* injection combined with ①, ②, ③, and ⑤ treatment (*n* = 52, before and after the PCI)	①, ②, ③, and ⑤ treatment (*n* = 52, before and after the PCI)	Fourteen days; NR	(1) ECG	NR
									(2) Color Doppler ultrasound	
									(3) Indexes of inflammatory cytokines (sLoX-1, hs-CRP, and TNF-*α*)	
									(4) Blood stasis syndrome score	
									(5) MACE	

(61) Zhili et al. [94]	Heilongjiang Province; China; multicenters	AMI	80 (46/34)	E: 62.1 ± 7.9 C: 63.5 ± 7.8	NR	*Xuesaitong* injection combined with ② and ③ treatment (*n* = 40, before and after the PCI)	② and ③ treatment (*n* = 40, before and after the PCI)	Two weeks; NR	(1) Laboratory indexes (BNP and MMP-2)	NR
									(2) Indexes of inflammatory cytokines (hs-CRP and IL-6)	

(62) Lijun et al. [95]	Shandong Province; China; multicenters	STEMI	39 (23/16)	E: 57.6 ± 10.2 C: 55.4 ± 9.8	Combined diseases and personal history: E/C: diabetes: 8/6; hypertension: 8/6; smoke: 9/8	*Xuesaitong* injection combined with conventional western medical treatment (NR) (*n* = 20, after the PCI)	Conventional western medical treatment (NR) (*n* = 19, after the PCI)	Two days; Six months	(1) TIMI	I
									(2) ECG (ST-segment)	
									(3) Adverse events	
									(4) MACE	

(63) Zhongchun et al. [96]	Hunan Province; China; single center	ACS	92 (56/36)	E: 52.97 ± 10.42 C: 53.38 ± 9.46	Combined diseases and personal history: E/C: hypertension: 16/18; smoke: 21/20	*Xueshuantong* injection combined with ①, ②, ③, and ⑤ treatment (*n* = 46, after the PCI)	①, ②, ③, and ⑤ treatment (*n* = 46, after the PCI)	Two weeks; NR	(1) Blood lipid level	NR
									(2) Indexes of inflammatory cytokines (hs-CRP and TNF-*α*)	
									(3) ET-1	
									(4) MACE	

(64) Yingxin et al. [97]	Guangdong Province; China; single center	AMI	68 (37/31)	E: 60.23 ± 7.98 C: 59.84 ± 8.27	NR	*Xueshuantong* injection combined with ①, ②, ③, and ⑤ treatment (*n* = 34, after the PCI)	①, ②, ③, and ⑤ treatment (*n* = 34, after the PCI)	Three weeks; Twelve months	(1) Blood lipid level	NR
									(2) Indexes of inflammatory cytokines (hs-CRP, TNF-*α*, and NT-proBNP)	
									(3) Color Doppler ultrasound	
									(4) Rehabilitation results (QoF and Barthel score)	
									(5) MACE	

(65) Ni [98]	Shaanxi Province; China; single center	ACS	114 (71/43)	E: 47 to 78, 55.8 ± 4.4 C: 49 to 76, 55.4 ± 4.2	Classification of disease: E/C: AMI: 27/27; UA: 30/30	*Xueshuantong* injection combined with ①, ②, ③, and ⑤ treatment (*n* = 57, after the PCI)	①, ②, ③, and ⑤ treatment (*n* = 57, after the PCI)	One month; NR	(1) Blood lipid level	NE
									(2) Indexes of inflammatory cytokines (hs-CRP and IL-6)	
									(3) MACE	

(66) Yiguang et al. [99]	Beijing city; China; single center	ACS	64 (37/27)	E: 28 to 69, 55.68 ± 5.9 C: 26 to 68, 55.41 ± 5.63	Classification of disease: E/C: AMI: 14/13; UA: 19/17	*Xueshuantong* injection combined with ①, ②, ③, and ⑤ treatment (*n* = 32, after the PCI)	①, ②, ③, and ⑤ treatment (*n* = 32, after the PCI)	Fourteen to twenty-one days; One month	(1) Myocardial microcirculation perfusion	NR
									(2) Blood lipid level	
									(3) Indexes of inflammatory cytokines (hs-CRP and IL-6)	
									(4) Vascular endothelial functions (ET, Fg, and vWF)	
									(5) MACE	

(67) Caihong and Jiuxi [100]	Henan Province; China; multicenters	ACS	80 (47/33)	E: 55.7 ± 5.7 C: 55.4 ± 4.4	Classification of disease: E/C: AMI: 17/16; UA: 23/26	*Xueshuantong* injection combined with ①, ②, ③, and ⑤ treatment (*n* = 40, after the PCI)	①, ②, ③, and ⑤ treatment (*n* = 40, after the PCI)	NR; One month	(1) Myocardial microcirculation perfusion	NR
									(2) Blood lipid level	
									(3) Indexes of inflammatory cytokines (hs-CRP and IL-6)	
									(4) Vascular endothelial functions (ET, Fg, and vWF)	
									(5) MACE	

(68) Hongyu and Lan [101]	Hebei Province; China; multicenters	AMI	80 (47/33)	E: 34 to 72, 52.6 ± 10.3 C: 38 to 74, 53.4 ± 11.2	NR	*Yiqifumai* injection combined with ② and ③ treatment (*n* = 40, after the PCI)	② and ③ treatment (*n* = 40, after the PCI)	Seven days; NR	(1) Scores of TCM symptoms	NR
									(2) Color Doppler ultrasound	

*Notes.* AMI: acute myocardial infarction; E: experimental group; C: control group; NYHA: New York Heart Association; NR: not report; BMI: body mass index; MI: myocardial infarction; CRP: C-reactive protein; LVEF: center ventricular ejection fraction; TIMI: thrombolysis in myocardial infarction; ①: lipid lowering; ②: anticoagulant; ③: antiplatelet; ④: antihypertensive; ⑤: antimyocardial ischemia; ⑥: nitroglycerin injection; I: bleeding events; II: abnormal renal function; IV: angina pectoris or myocardial infarction; III: arrhythmia; V: heart failure; VI: allergy; VII: headache; IX: abnormal digestive system; VIII: dizziness; IX: respiratory system disfunction.

## Supplementary Materials

Supplementary Materials Figure S1: risk of bias graph. Figure S2: risk of bias summary. Figure S3: subgroup analysis of hs-CRP based on the classification of area. Figure S4: subgroup analysis of hs-CRP based on the classification of levels of hospital. Figure S5: subgroup analysis of hs-CRP based on the classification of sample size. Figure S6: subgroup analysis of IL-6 based on the classification of area. Figure S7: subgroup analysis of IL-6 based on the classification of levels of hospital. Figure S8: subgroup analysis of IL-6 based on the classification of sample size. Figure S9: Egger's and Begg's test for clinical efficiency. Figure S10: Egger's and Begg's test for hs-CRP. Figure S11: Egger's and Begg's test for MI. Figure S12: Egger's and Begg's test for stenocardia. Figure S13: specification of *Danhong* injection. Figure S14: specification of *Safflower yellow* injection. Figure S15: specification of *Kudiezi* injection. Figure S16: specification of *Dazhuhongjingtian* injection. Figure S17: specification of *Shuxuetong* injection. Figure S18: specification of *Xuesaitong* injection. Figure S19: specification of *Guanxinning* injection. Figure S20: specification of *Shengmai* injection. Figure S21: specification of *Shenmai* injection. Figure S22: specification of *Xiangdan* injection. Figure S23: specification of *Gualoupi* injection. Figure S24: specification of *Xueshuantong* injection. Figure S25: specification of *Safflower* injection. Figure S26: specification of *Danshen* injection. Figure S27: specification of *Dengzhanhuasu* injection. Figure S28: specification of *Yiqifumai *injection. Table S1: table of the risk of bias summary. Table S2: the detailed information of included TCMI.
