# Supplementary material for: The Application of Traditional Chinese Medicine Injection on Patients with Acute Coronary Syndrome during the Perioperative Period of Percutaneous Coronary Intervention: A Systematic Review and Meta-Analysis of Randomized Controlled Trials
Source: Evid Based Complement Alternat Med. 2020 May 18;2020:3834128. doi: 10.1155/2020/3834128 (PMC7254090; doi:10.1155/2020/3834128)
Supplement: Supplementary Materials — Figure S1: risk of bias graph. Figure S2: risk of bias summary. Figure S3: subgroup analysis of hs-CRP based on the classification of area. Figure S4: subgroup analysis of hs-CRP based on the classification of levels of hospital. Figure S5: subgroup analysis of hs-CRP based on the classification of sample size. Figure S6: subgroup analysis of IL-6 based on the classification of area. Figure S7: subgroup analysis of IL-6 based on the classification of levels of hospital. Figure S8: subgroup analysis of IL-6 based on the classification of sample size. Figure S9: Egger's and Begg's test for clinical efficiency. Figure S10: Egger's and Begg's test for hs-CRP. Figure S11: Egger's and Begg's test for MI. Figure S12: Egger's and Begg's test for stenocardia. Figure S13: specification of Danhong injection. Figure S14: specification of Safflower yellow injection. Figure S15: specification of Kudiezi injection. Figure S16: specification of Dazhuhongjingtian injection. Figure S17: specification of Shuxuetong injection. Figure S18: specification of Xuesaitong injection. Figure S19: specification of Guanxinning injection. Figure S20: specification of Shengmai injection. Figure S21: specification of Shenmai injection. Figure S22: specification of Xiangdan injection. Figure S23: specification of Gualoupi injection. Figure S24: specification of Xueshuantong injection. Figure S25: specification of Safflower injection. Figure S26: specification of Danshen injection. Figure S27: specification of Dengzhanhuasu injection. Figure S28: specification of Yiqifumai injection. Table S1: table of the risk of bias summary. Table S2: the detailed information of included TCMI. [file 3834128.f1.pdf]

Supplementary Materials

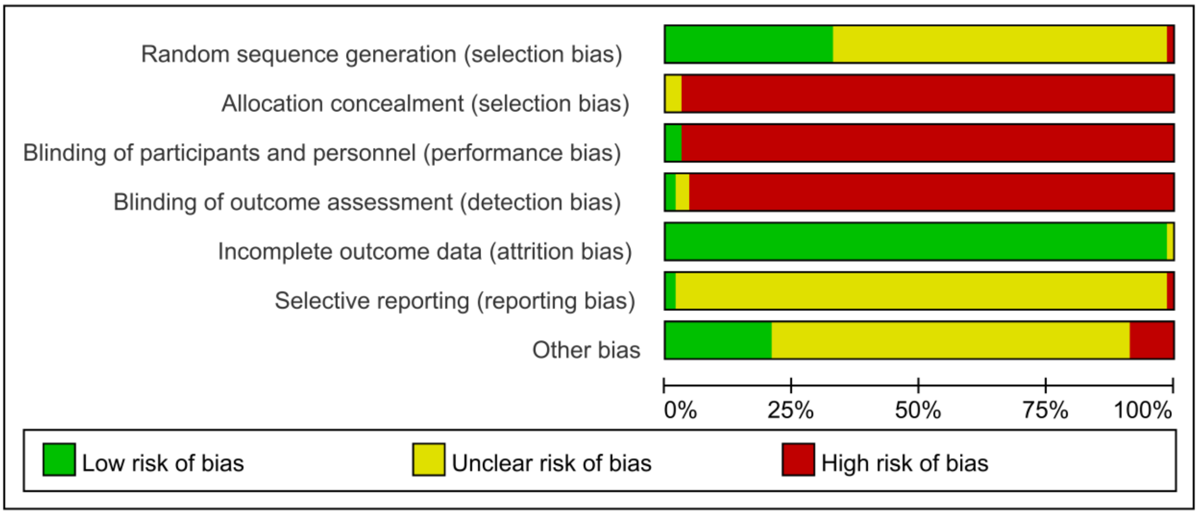

Figure S1: Risk of bias graph.

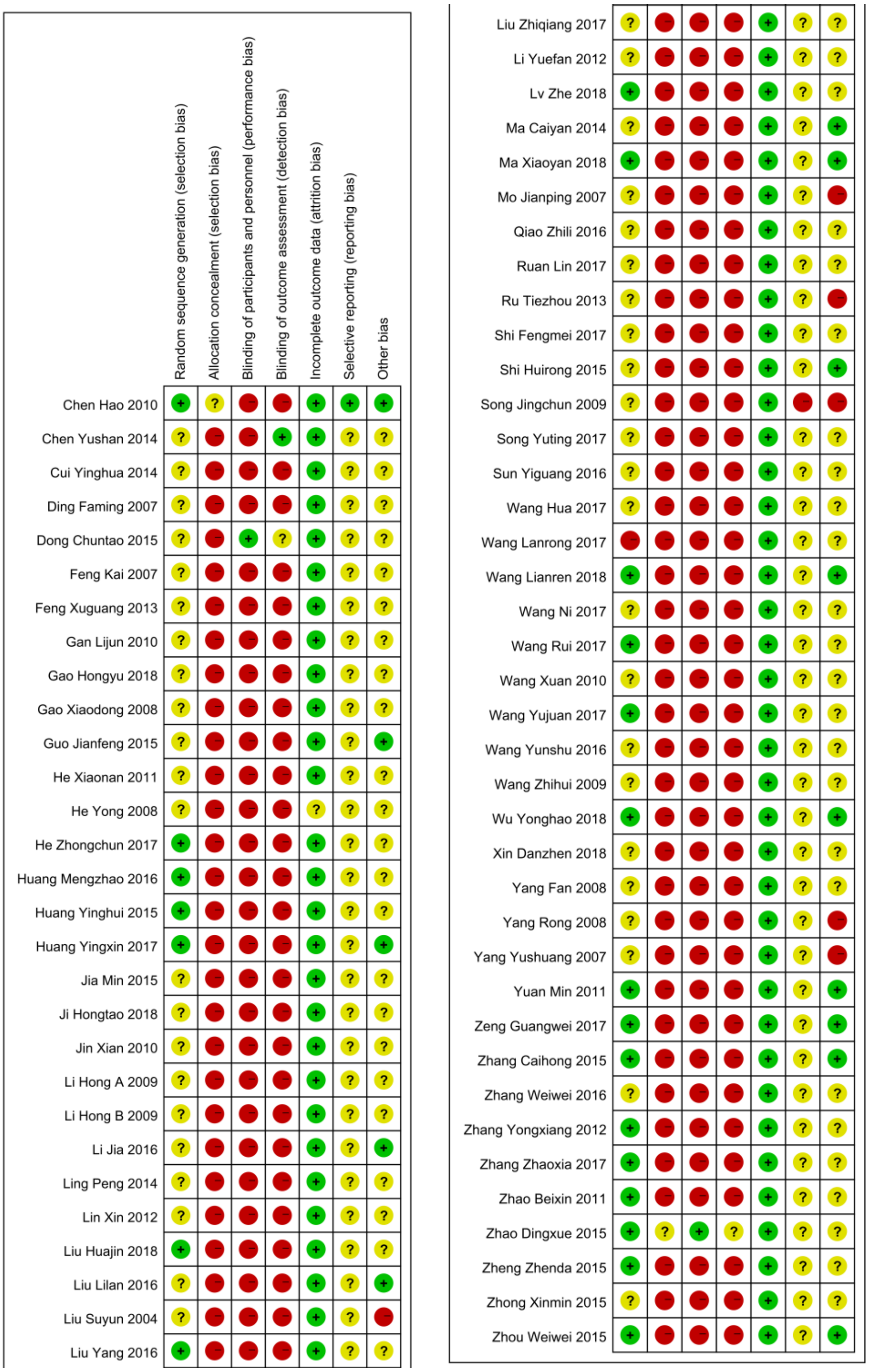

Figure S2: Risk of bias summary.

Table S1: Table of the risk of bias summary

Study	Random sequence generation	Allocation concealment	Blinding of participants and personnel	Blinding of outcome assessment	Incomplete outcome data	Selective reporting	Other bias	Risk of bias
1. Chen Hao 2010	Y	U	N	N	Y	Y	Y	Low
2. Chen Yushan 2 2014	U	N	N	Y	Y	U	U	Moderate
3. Cui Yinghua 2014	U	N	N	N	Y	U	U	High
4. Ding Faming 2007	U	N	N	N	Y	U	U	High
5. Dong Chuntao 2015	U	N	Y	U	Y	U	U	Moderate
6. Feng Kai 2007	U	N	N	N	Y	U	U	High
7. Feng Xuguang 2013	U	N	N	N	Y	U	U	High
8. Gan Lijun 2010	U	N	N	N	Y	U	U	High
9. Gao Hongyu 2018	U	N	N	N	Y	U	U	High
10. Gao Xiaodong 2008	U	N	N	N	Y	U	U	High
11. Guo Jianfeng 2015	U	N	N	N	Y	U	Y	Moderate
12. He Xiaonan 2011	U	N	N	N	Y	U	U	High
13. He Yong 2008	U	N	N	N	U	U	U	High
14. He Zhongchun 2017	Y	N	N	N	Y	U	U	Moderate
15. Huang Mengzhao 2016	Y	N	N	N	Y	U	U	Moderate
16. Huang Yinghui 2015	Y	N	N	N	Y	U	U	Moderate
17. Huang Yinxin 2017	Y	N	N	N	Y	U	Y	Moderate
18. Jia Min 2015	U	N	N	N	Y	U	U	High
19. Ji Hongtao 2018	U	N	N	N	Y	U	U	High
20. Jin Xian 2010	U	N	N	N	Y	U	U	High
21. Li Hong A 2009	U	N	N	N	Y	U	U	High
22. Li Hong B 2009	U	N	N	N	Y	U	U	High
23. Li Jia 2016	U	N	N	N	Y	U	Y	High
24. Ling Peng 2014	U	N	N	N	Y	U	U	High
25. Lin Xin 2012	U	N	N	N	Y	U	U	High
26. Liu Huajin 2018	Y	N	N	N	Y	U	U	Moderate
27. Liu Lilan 2016	U	N	N	N	Y	U	Y	High
28. Liu Suyun 2004	U	N	N	N	Y	U	N	High
29. Liu Yang 2016	Y	N	N	N	Y	U	U	Moderate
30. Liu Zhiqing 2017	U	N	N	N	Y	U	U	High
31. Li Yuefan 2012	U	N	N	N	Y	U	U	High
32. Lv Zhe 2018	Y	N	N	N	Y	U	U	Moderate
33. Ma Caiyan 2014	U	N	N	N	Y	U	Y	High
34. Ma Xiaoyan 2018	Y	N	N	N	Y	U	Y	Moderate
35. Mo Jianping 2007	U	N	N	N	Y	U	N	High
36. Qiao Zhili 2016	U	N	N	N	Y	U	U	High
37. Ruan Lin 2017	U	N	N	N	Y	U	U	High
38. Re Tiejun 2013	U	N	N	N	Y	U	N	High
39. Shi Fengmei 2017	U	N	N	N	Y	U	U	High
40. Shi Huirong 2015	U	N	N	N	Y	U	Y	Moderate

41. Song Junchun 2009	U	N	N	N	Y	N	N	High
42. Song Yuting 2017	U	N	N	N	Y	U	U	High
43. Sun Yiguang 2016	U	N	N	N	Y	U	U	High
44. Wang Hua 2017	U	N	N	N	Y	U	U	High
45. Wang Lanrong 2017	N	N	N	N	Y	U	U	High
46. Wang Lianren 2018	Y	N	N	N	Y	U	Y	Moderate
47. Wang Ni 2017	U	N	N	N	Y	U	U	High
48. Wang Rui 2017	Y	N	N	N	Y	U	U	Moderate
49. Wang Xuan 2010	U	N	N	N	Y	U	U	High
50. Wang Yujuan 2017	Y	N	N	N	Y	U	U	Moderate
51. Wang Yunshu 2016	U	N	N	N	Y	U	U	High
52. Wang Zhihui 2009	U	N	N	N	Y	U	U	High
53. Wu Yonghao 2018	Y	N	N	N	Y	U	Y	Moderate
54. Xin Danzhen 2018	U	N	N	N	Y	U	U	High
55. Yang Fan 2008	U	N	N	N	Y	U	U	High
56. Yang Rong 2008	U	N	N	N	Y	U	N	High
57. Yang Yushuang 2007	U	N	N	N	Y	U	N	High
58. Yuan Min 2011	Y	N	N	N	Y	U	Y	Moderate
59. Zeng Guangwei 2017	Y	N	N	N	Y	U	Y	Moderate
60. Zhang Caihong 2015	Y	N	N	N	Y	U	Y	Moderate
61. Zhang Weiwei 2016	U	N	N	N	Y	U	U	High
62. Zhang Yongxiang 2012	Y	N	N	N	Y	U	U	Moderate
63. Zhang Zhaoxia 2017	Y	N	N	N	Y	U	U	Moderate
64. Zhao Beixin 2011	Y	N	N	N	Y	U	U	Moderate
65. Zhao Dingxue 2015	Y	U	Y	U	Y	U	U	Moderate
66. Zheng Zheda 2015	Y	N	N	N	Y	U	U	Moderate
67. Zhong Xinmin 2015	U	N	N	N	Y	U	U	High
68. Zhou Weiwei 2015	Y	N	N	N	Y	U	Y	Moderate

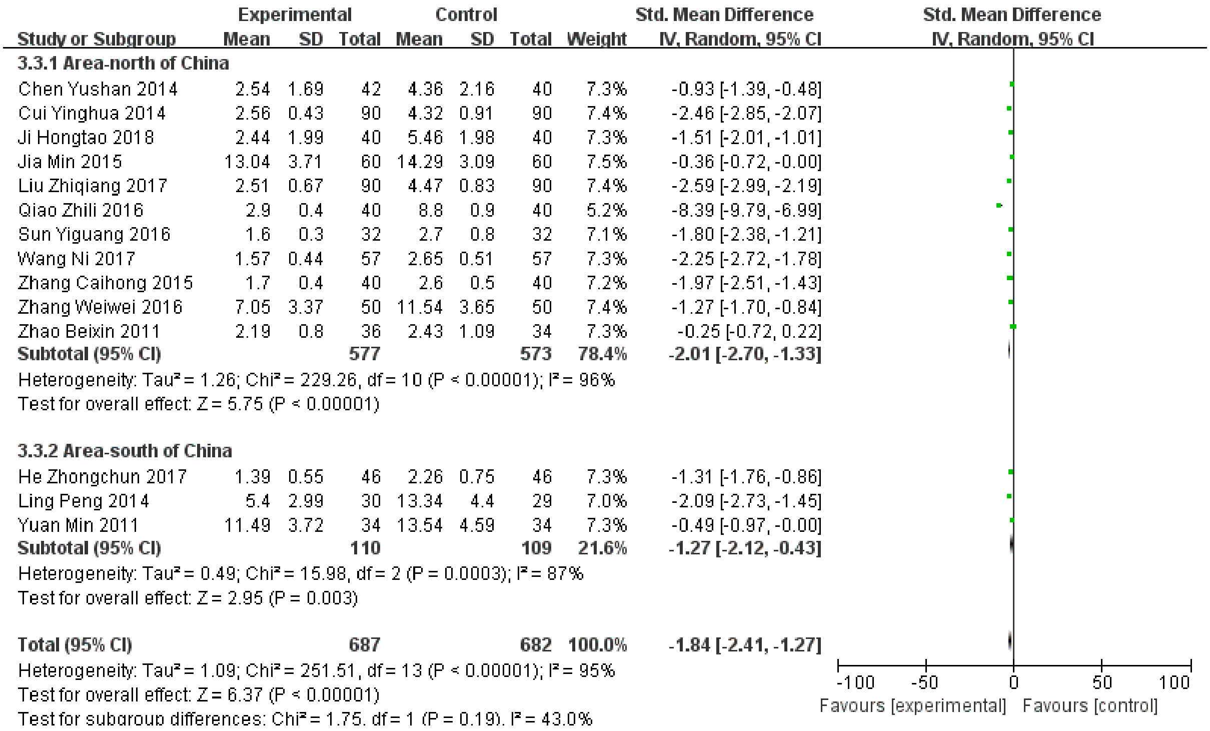

Figure S3: Subgroup analysis of hs-CRP based on the classification of area (north or south of China).

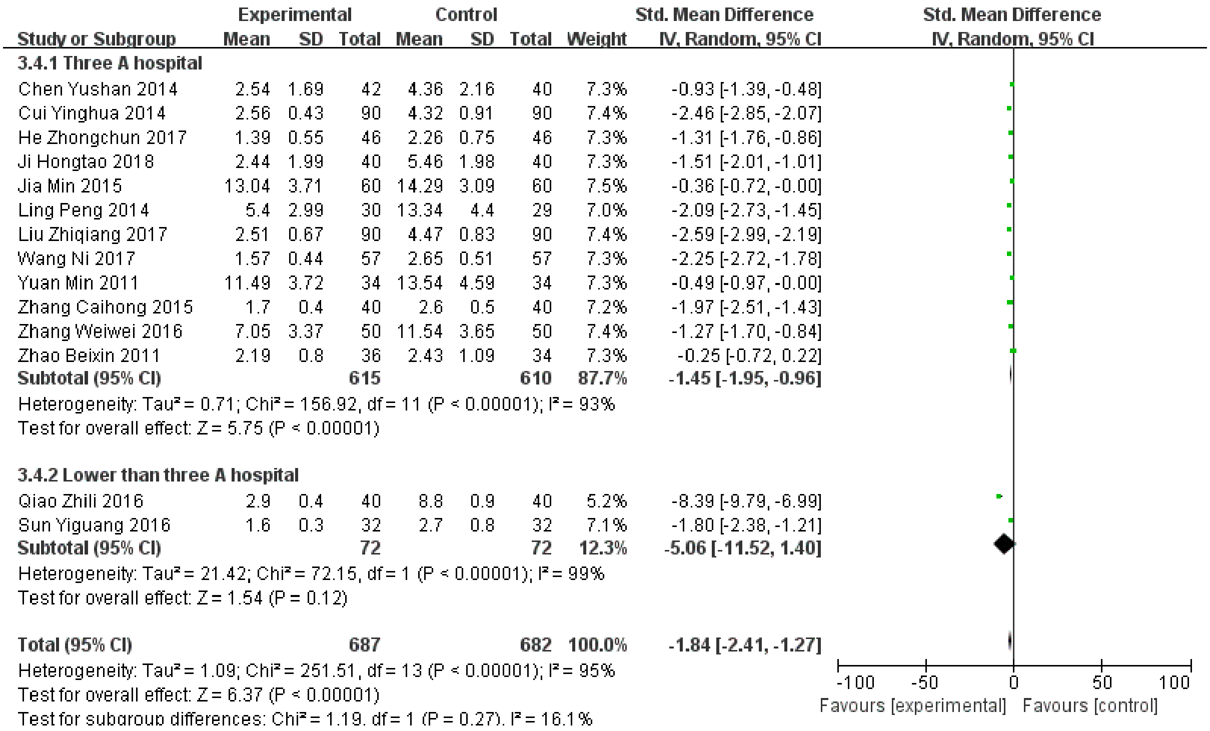

Figure S4: Subgroup analysis of hs-CRP based on the classification of levels of hospital (three A hospital or not).

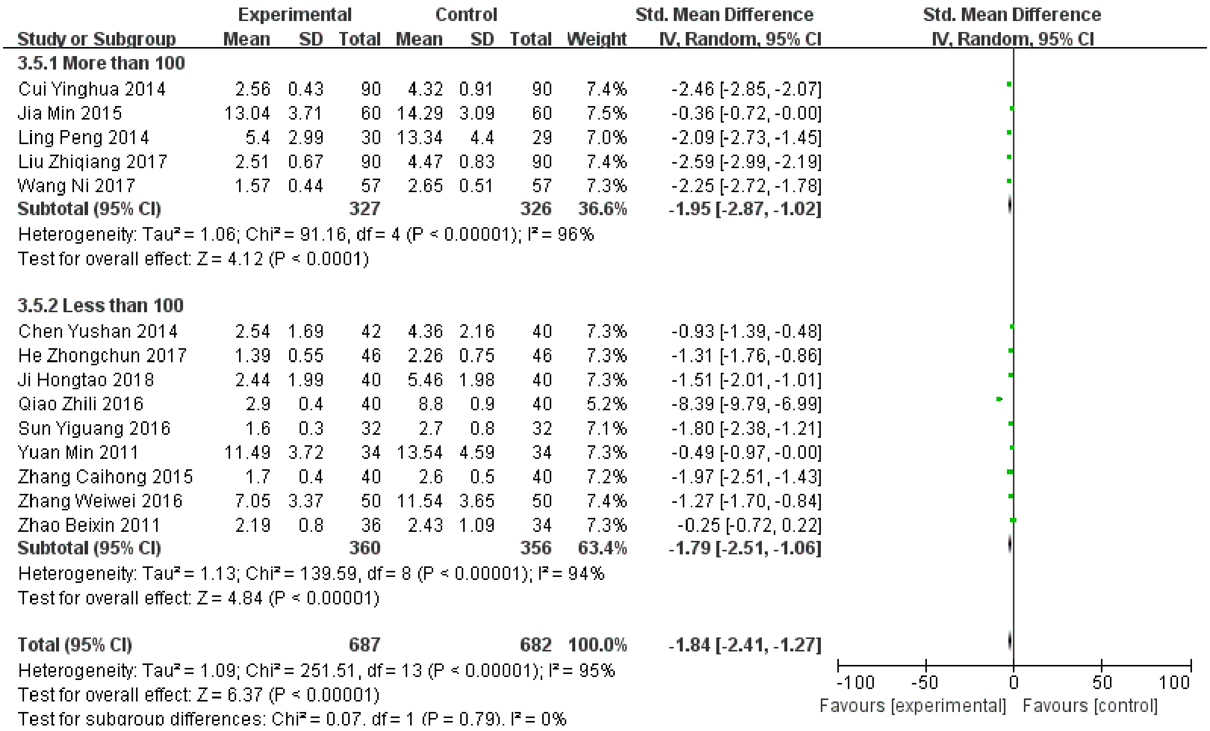

Figure S5: Subgroup analysis of hs-CRP based on the classification of sample size (more than 100 or not).

.

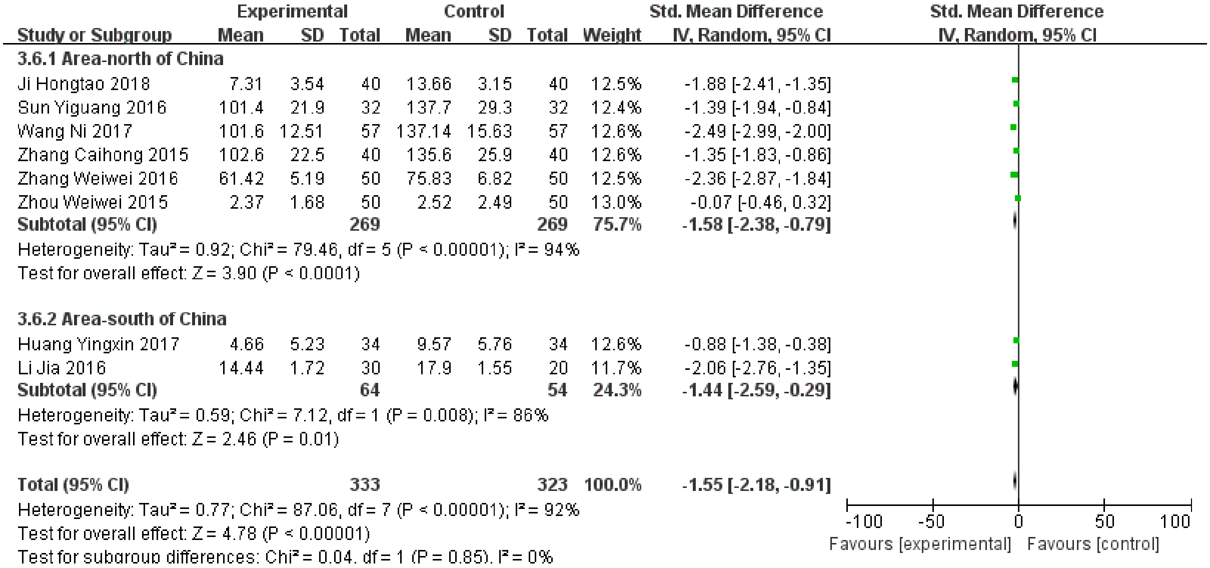

Figure S6: Subgroup analysis of IL-6 based on the classification of area (north or south of China).

Study or Subgroup	Experimental Mean	Experimental SD	Experimental Total	Control Mean	Control SD	Control Total	Weight	Std. Mean Difference IV, Random, 95% CI
**3.7.1 Three A hospital**								
Wang Ni 2017	101.6	12.51	57	137.14	15.63	57	12.6%	-2.49 [-2.99, -2.00]
Zhang Caihong 2015	102.6	22.5	40	135.6	25.9	40	12.6%	-1.35 [-1.83, -0.86]
Zhang Weiwei 2016	61.42	5.19	50	75.83	6.82	50	12.5%	-2.36 [-2.87, -1.84]
**Subtotal (95% CI)**			**147**			**147**	**37.8%**	**-2.06 [-2.78, -1.35]**
Heterogeneity: Tau² = 0.34; Chi² = 12.47, df = 2 (P = 0.002); I² = 84%								
Test for overall effect: Z = 5.63 (P < 0.00001)								
**3.7.2 Lower than three A hospital**								
Huang Yingxin 2017	4.66	5.23	34	9.57	5.76	34	12.6%	-0.88 [-1.38, -0.38]
Ji Hongtao 2018	7.31	3.54	40	13.66	3.15	40	12.5%	-1.88 [-2.41, -1.35]
Li Jia 2016	14.44	1.72	30	17.9	1.55	20	11.7%	-2.06 [-2.76, -1.35]
Sun Yiguang 2016	101.4	21.9	32	137.7	29.3	32	12.4%	-1.39 [-1.94, -0.84]
Zhou Weiwei 2015	2.37	1.68	50	2.52	2.49	50	13.0%	-0.07 [-0.46, 0.32]
**Subtotal (95% CI)**			**186**			**176**	**62.2%**	**-1.23 [-1.99, -0.47]**
Heterogeneity: Tau² = 0.68; Chi² = 42.89, df = 4 (P < 0.00001); I² = 91%								
Test for overall effect: Z = 3.17 (P = 0.002)								
**Total (95% CI)**			**333**			**323**	**100.0%**	**-1.55 [-2.18, -0.91]**
Heterogeneity: Tau² = 0.77; Chi² = 87.06, df = 7 (P < 0.00001); I² = 92%								
Test for overall effect: Z = 4.78 (P < 0.00001)								
Test for subgroup differences: Chi² = 2.44, df = 1 (P = 0.12), I² = 58.9%								

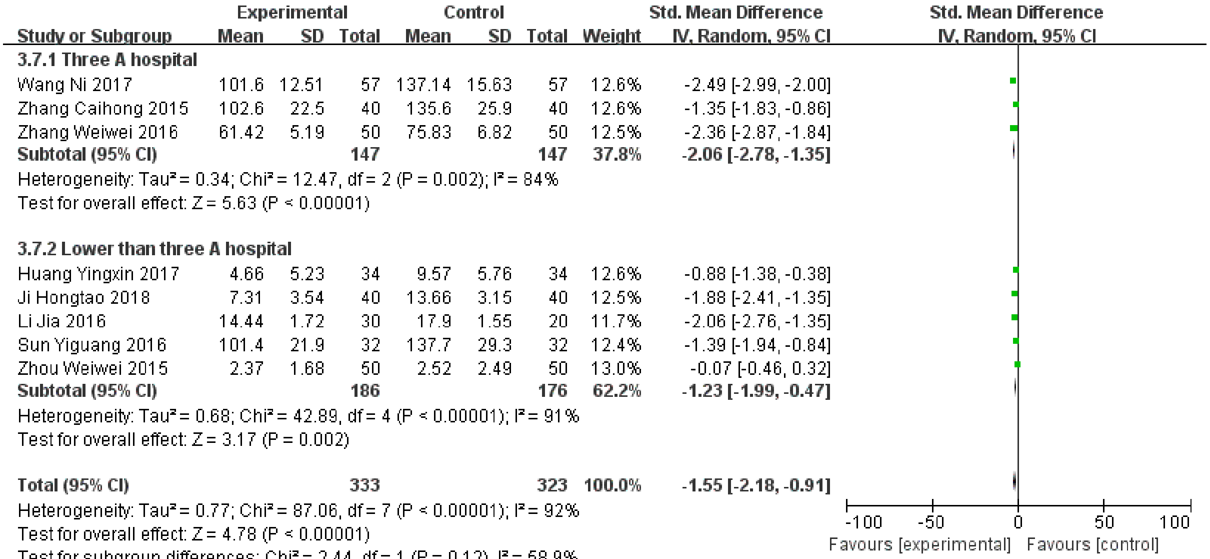

Figure S7: Subgroup analysis of IL-6 based on the classification of levels of hospital (three A hospital or not).

Study or Subgroup	Experimental Mean	Experimental SD	Experimental Total	Control Mean	Control SD	Control Total	Weight	Std. Mean Difference IV, Random, 95% CI
**3.8.1 More than 100**								
Wang Ni 2017	101.6	12.51	57	137.14	15.63	57	12.6%	-2.49 [-2.99, -2.00]
**Subtotal (95% CI)**			**57**			**57**	**12.6%**	**-2.49 [-2.99, -2.00]**
Heterogeneity: Not applicable								
Test for overall effect: Z = 9.91 (P < 0.00001)								
**3.8.2 Less than 100**								
Huang Yingxin 2017	4.66	5.23	34	9.57	5.76	34	12.6%	-0.88 [-1.38, -0.38]
Ji Hongtao 2018	7.31	3.54	40	13.66	3.15	40	12.5%	-1.88 [-2.41, -1.35]
Li Jia 2016	14.44	1.72	30	17.9	1.55	20	11.7%	-2.06 [-2.76, -1.35]
Sun Yiguang 2016	101.4	21.9	32	137.7	29.3	32	12.4%	-1.39 [-1.94, -0.84]
Zhang Caihong 2015	102.6	22.5	40	135.6	25.9	40	12.6%	-1.35 [-1.83, -0.86]
Zhang Weiwei 2016	61.42	5.19	50	75.83	6.82	50	12.5%	-2.36 [-2.87, -1.84]
Zhou Weiwei 2015	2.37	1.68	50	2.52	2.49	50	13.0%	-0.07 [-0.46, 0.32]
**Subtotal (95% CI)**			**276**			**266**	**87.4%**	**-1.41 [-2.05, -0.77]**
Heterogeneity: Tau² = 0.67; Chi² = 65.52, df = 6 (P < 0.00001); I² = 91%								
Test for overall effect: Z = 4.32 (P < 0.0001)								
**Total (95% CI)**			**333**			**323**	**100.0%**	**-1.55 [-2.18, -0.91]**
Heterogeneity: Tau² = 0.77; Chi² = 87.06, df = 7 (P < 0.00001); I² = 92%								
Test for overall effect: Z = 4.78 (P < 0.00001)								
Test for subgroup differences: Chi² = 6.92, df = 1 (P = 0.009), I² = 85.5%								

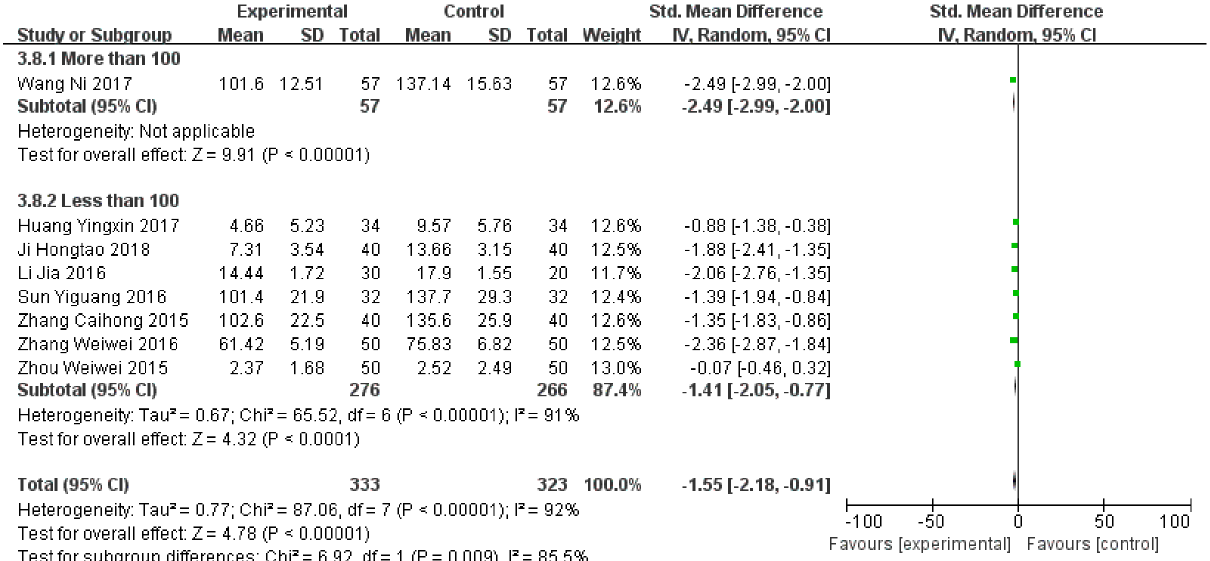

Figure S8: Subgroup analysis of IL-6 based on the classification of sample size (more than 100 or not).

```
Egger's test for small-study effects:
Regress standard normal deviate of intervention
effect estimate against its standard error

Number of studies =  15                          Root MSE      =   .4154
------------------------------------------------------------------------------
     Std_Eff |      Coef.   Std. Err.      t    P>|t|     [95% Conf. Interval]
-------------+----------------------------------------------------------------
       slope |    .748662   .3526998     2.12   0.054    -.0132995    1.510624
        bias |   .0269119   .5558865     0.05   0.962    -1.174008    1.227832
------------------------------------------------------------------------------

Test of H0: no small-study effects          P = 0.962


Begg's Test

  adj. Kendall's Score (P-Q) =        -6
          Std. Dev. of Score =     20.21 (corrected for ties)
           Number of Studies =        15
                          z  =     -0.30
                    Pr > |z| =     0.767
                          z  =      0.25 (continuity corrected)
                    Pr > |z| =     0.805 (continuity corrected)
```

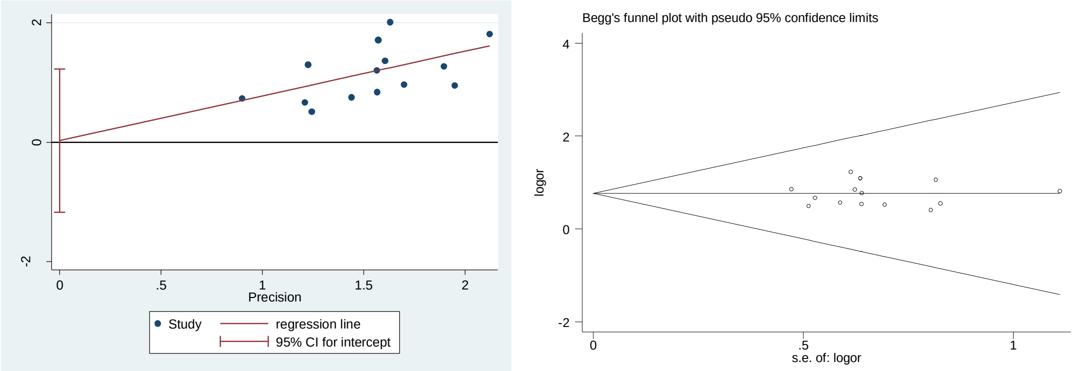

Figure S9: *Egger's* and *Begg's* test for clinical efficiency.

```
Egger's test for small-study effects:
Regress standard normal deviate of intervention
effect estimate against its standard error

Number of studies =  14                          Root MSE      =    8.84
------------------------------------------------------------------------------
     Std_Eff |      Coef.   Std. Err.      t    P>|t|     [95% Conf. Interval]
-------------+----------------------------------------------------------------
       slope |   -1.14463   .6636727    -1.72   0.110    -2.590649    .3013887
        bias |  -3.767647   4.217848    -0.89   0.389    -12.95755    5.422255
------------------------------------------------------------------------------

Test of H0: no small-study effects          P = 0.389

Begg's Test

  adj. Kendall's Score (P-Q) =       -35
          Std. Dev. of Score =     18.27
           Number of Studies =        14
                          z  =     -1.92
                   Pr > |z|  =     0.055
                          z  =      1.86 (continuity corrected)
                   Pr > |z|  =     0.063 (continuity corrected)
```

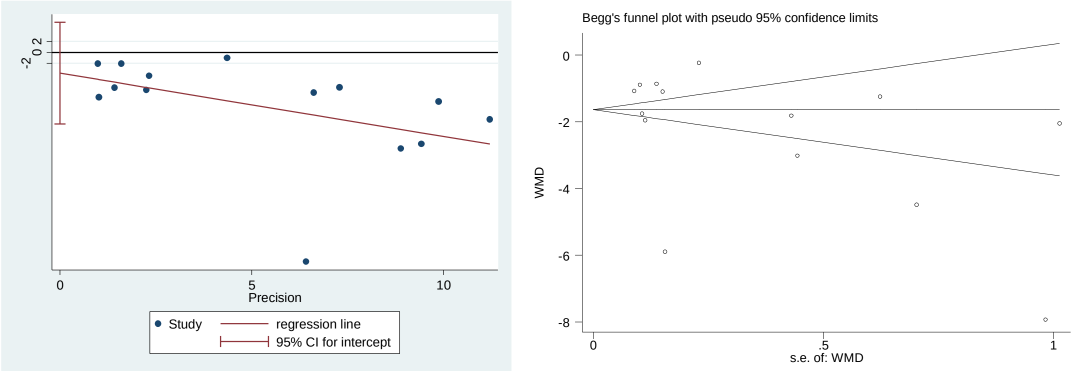

Figure S10: *Egger's* and *Begg's* test for hs-CRP.

```
Egger's test for small-study effects:
Regress standard normal deviate of intervention
effect estimate against its standard error

Number of studies =  8                                    Root MSE      =   15.37
------------------------------------------------------------------------------
     Std_Eff |     Coef.   Std. Err.      t    P>|t|     [95% Conf. Interval]
-------------+----------------------------------------------------------------
       slope |  97.30773   26.82251     3.63   0.011     31.67542    162.9401
        bias | -145.3209   25.34468    -5.73   0.001    -207.3371   -83.30474
------------------------------------------------------------------------------

Test of H0: no small-study effects          P = 0.001

Begg's Test

  adj. Kendall's Score (P-Q) =      -22
          Std. Dev. of Score =     8.08
           Number of Studies =        8
                          z  =    -2.72
                    Pr > |z| =    0.006
                          z  =     2.60 (continuity corrected)
                    Pr > |z| =    0.009 (continuity corrected)
```

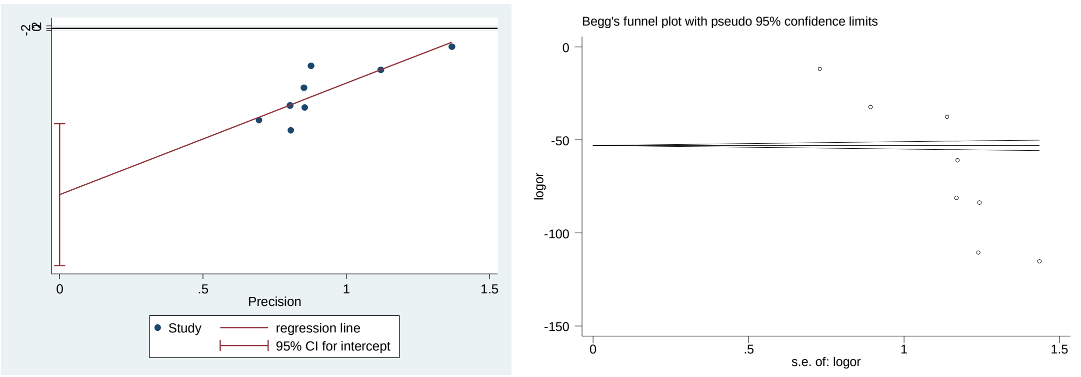

Figure S11: *Egger's* and *Begg's* test for MI.

```
Egger's test for small-study effects:
Regress standard normal deviate of intervention
effect estimate against its standard error

Number of studies =  15                        Root MSE      =   20.22

      Std_Eff |      Coef.   Std. Err.      t    P>|t|     [95% Conf. Interval]
--------------+-----------------------------------------------------------------
        slope |   51.07891   18.64388     2.74   0.017     10.80125    91.35658
         bias |  -92.14951   22.58512    -4.08   0.001    -140.9417   -43.35733

Test of H0: no small-study effects          P = 0.001

Begg's Test

  adj. Kendall's Score (P-Q) =      -47
          Std. Dev. of Score =    20.21
           Number of Studies =       15
                          z  =    -2.33
                    Pr > |z| =    0.020
                          z  =     2.28 (continuity corrected)
                    Pr > |z| =    0.023 (continuity corrected)
```

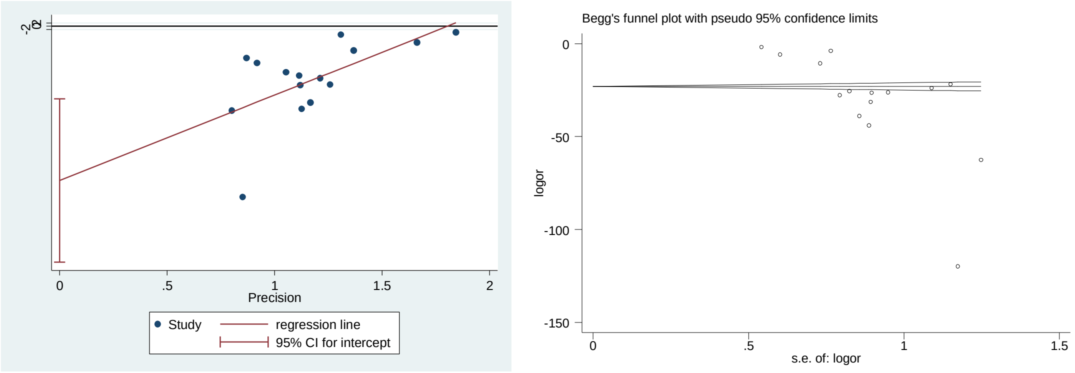

Figure S12: *Egger's* and *Begg's* test for stenocardia.

Table S2. The detailed information of included TCMI.

**Name of TCMI**	**Constituents of TCMI**	**Latin names of constituents for Chinese medicine**	**Ratios of constituents**	**Specifications**	**Clinical use of the TCMI**	**Chinese national medicine permission number of TCMI**
**Injection of Dazhuhongjingtian**	Dazhuhongjingtian	*Rhodiolae Crenulatae Radixet Rhizoma*	—	5ml	Coronary heart disease, MI, Stenocardia	Z20060361
**Injection of Shuxuetong**	Shuizhi, Dilong	*Hirudo, Pheretima*	—	2ml	Stroke, Stenocardia	Z20010100
**Injection of Shenmai**	Hongshen, Maidong, Polysorbate 80, Sodium chloride	*Ginseng Radix et Rhizoma Rubra, Ophiopogonis Radix*	—	2ml<br>5ml<br>10ml<br>20ml<br>50ml<br>100ml	Cardiogenic shock, Coronary heart disease, Pulmonary heart disease	Z33020023(2ml)<br>Z33020022(5ml)<br>Z33020021(10ml)<br>Z33020020(20ml)<br>Z33020019(50ml)<br>Z33020018(100ml)
**Injection of Danshen**	Danshen, Polysorbate 80	*Salviae Miltiorrhizae radix Et Rhizoma*	—	10ml	Coronary heart disease, MI, Stenocardia	Z13020777
**Injection of Danhong**	Danshen, Honghua, Water for injection	*Salviae Miltiorrhizae radix Et Rhizoma, Carthami Flos*	—	10ml	Coronary heart disease, Stenocardia, MI,	Z20026866

					Stroke, Pulmonary heart disease	
**Injection of Dengzhanhuasu**	Dengzhanhuasu, Edetate disodium, Glycerinum, Sodium bicarbonate	*Breviscapine*	—	5ml: 20mg	Stroke sequela, Coronary heart disease	Z13020778
**Injection of Gualoupi**	Gualoupi, Water for injection	*Trichosabthis Pericarpium*		4ml	Coronary heart disease, Stenocardia	Z20027540
**Injection of Guanxinning**	Danshen, Chuanxiong,	*Salviae Miltiorrhizae radix Et Rhizoma, Chuanxiong Rhizoma*	—	2ml 10ml	Coronary heart disease, Stenocardia	Z33020051(2ml) Z33021101(10ml)
**Injection of Kudiezi**	Kudiezi	*Ixeris Sonchifolia (Bunge) Hance*	—	20ml	Coronary heart disease, Stenocardia	Z20025449
**Injection of Shengmai**	Hongshen, Maidong, Wuweizi, Polysorbate 80	*Ginseng Radix et Rhizoma Rubra, Ophiopogonis Radix, Schisandrae Chinensis Fructus*	—	10ml	MI, Cardiogenic shock, Septic shock	Z51021882
**Injection of Xiangdan**	Danshen, Jiangxiang, Polysorbate	*Salviae Miltiorrhizae radix Et*	—	2ml 10ml	Coronary heart disease,	Z33020055 (2ml) Z33020054

	80	*Rhizoma, Dalbergiae Odoriferae Lignum*			Stenocardia, MI	(2ml)
**Injection of Xuesaitong**	Sanqi Zongzaogan	*Notoginseng Total Saponins*	—	10ml: 0.25g	Atherosclerosis, Stroke, Cerebral embolism	Z23020786
**Injection of Xueshuantong**	Sanqi Zongzaogan, Sodium chloride, Sodium citrate	*Notoginseng Total Saponins*	—	2ml: 70mg	Coronary heart disease, Stenocardia, Cardiogenic shock,	Z44020285
**Injection of Yiqifumai**	Hongshen, Maidong, Wuweizi, Mannitol, N-methylglucamine	*Ginseng Radix et Rhizoma Rubra, Ophiopogonis Radix, Schisandrae Chinensis Fructus*	—	0.65g	Coronary heart disease, Stenocardia	Z20060463

Note: All information of TCMI was supported by the drug package inserts and Chinese pharmacopoeia.

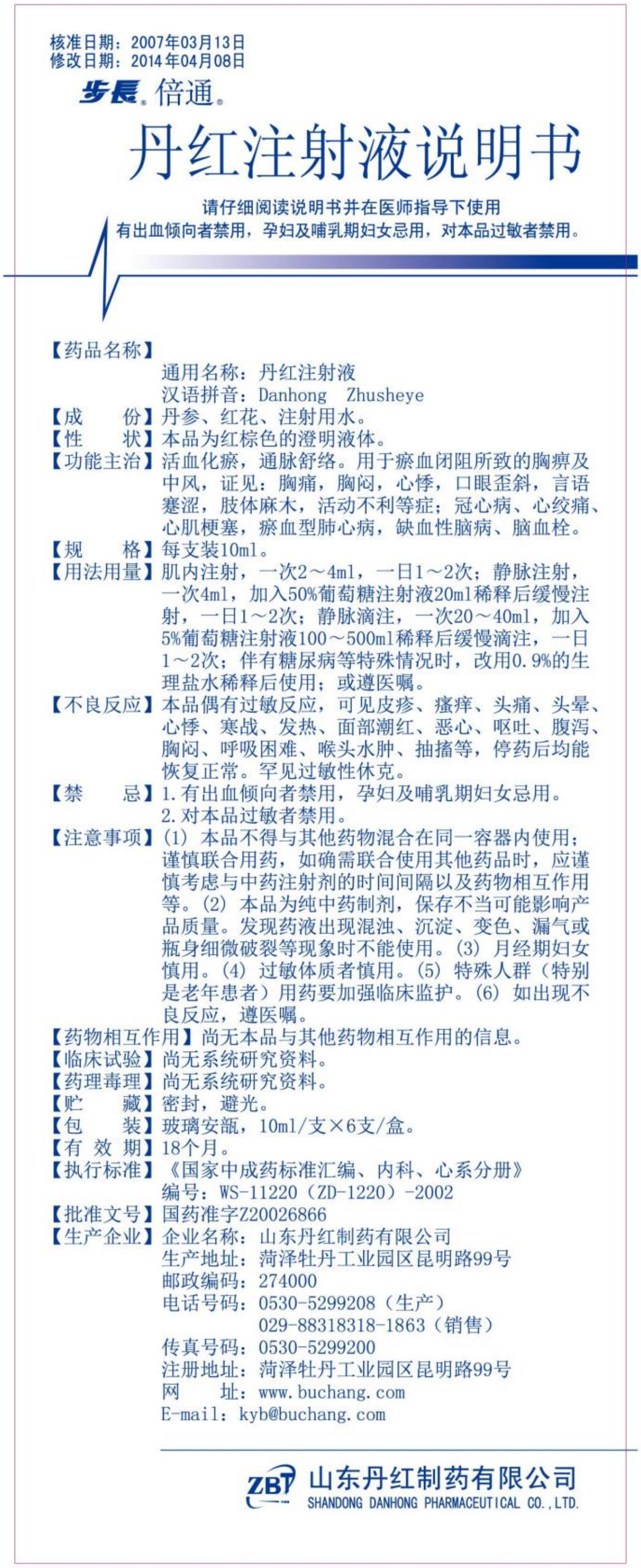

核准日期：2007年03月13日
修改日期：2014年04月08日

步長。倍通。

丹红注射液说明书

请仔细阅读说明书并在医师指导下使用
有出血倾向者禁用，孕妇及哺乳期妇女忌用，对本品过敏者禁用。

【药品名称】
通用名称：丹红注射液
汉语拼音：Danhong Zhusheye
【成　　份】丹参、红花、注射用水。
【性　　状】本品为红棕色的澄明液体。
【功能主治】活血化瘀，通脉舒络。用于瘀血闭阻所致的胸痹及中风，证见：胸痛，胸闷，心悸，口眼歪斜，言语蹇涩，肢体麻木，活动不利等症；冠心病、心绞痛、心肌梗塞，瘀血型肺心病，缺血性脑病、脑血栓。
【规　　格】每支装10ml。
【用法用量】肌内注射，一次2～4ml，一日1～2次；静脉注射，一次4ml，加入50%葡萄糖注射液20ml稀释后缓慢注射，一日1～2次；静脉滴注，一次20～40ml，加入5%葡萄糖注射液100～500ml稀释后缓慢滴注，一日1～2次；伴有糖尿病等特殊情况时，改用0.9%的生理盐水稀释后使用；或遵医嘱。
【不良反应】本品偶有过敏反应，可见皮疹、瘙痒、头痛、头晕、心悸、寒战、发热、面部潮红、恶心、呕吐、腹泻、胸闷、呼吸困难、喉头水肿、抽搐等，停药后均能恢复正常。罕见过敏性休克。
【禁　　忌】1.有出血倾向者禁用，孕妇及哺乳期妇女忌用。
2.对本品过敏者禁用。
【注意事项】(1) 本品不得与其他药物混合在同一容器内使用；谨慎联合用药，如确需联合使用其他药品时，应谨慎考虑与中药注射剂的时间间隔以及药物相互作用等。(2) 本品为纯中药制剂，保存不当可能影响产品质量。发现药液出现混浊、沉淀、变色、漏气或瓶身细微破裂等现象时不能使用。(3) 月经期妇女慎用。(4) 过敏体质者慎用。(5) 特殊人群（特别是老年患者）用药要加强临床监护。(6) 如出现不良反应，遵医嘱。
【药物相互作用】尚无本品与其他药物相互作用的信息。
【临床试验】尚无系统研究资料。
【药理毒理】尚无系统研究资料。
【贮　　藏】密封，避光。
【包　　装】玻璃安瓿，10ml/支×6支/盒。
【有 效 期】18个月。
【执行标准】《国家中成药标准汇编、内科、心系分册》
编号：WS-11220（ZD-1220）-2002
【批准文号】国药准字Z20026866
【生产企业】企业名称：山东丹红制药有限公司
生产地址：菏泽牡丹工业园区昆明路99号
邮政编码：274000
电话号码：0530-5299208（生产）
029-88318318-1863（销售）
传真号码：0530-5299200
注册地址：菏泽牡丹工业园区昆明路99号
网　　址：www.buchang.com
E-mail：kyb@buchang.com

ZBT 山东丹红制药有限公司
SHANDONG DANHONG PHARMACEUTICAL CO.,LTD.

Figure S13: Specification of *Danhong* injection.

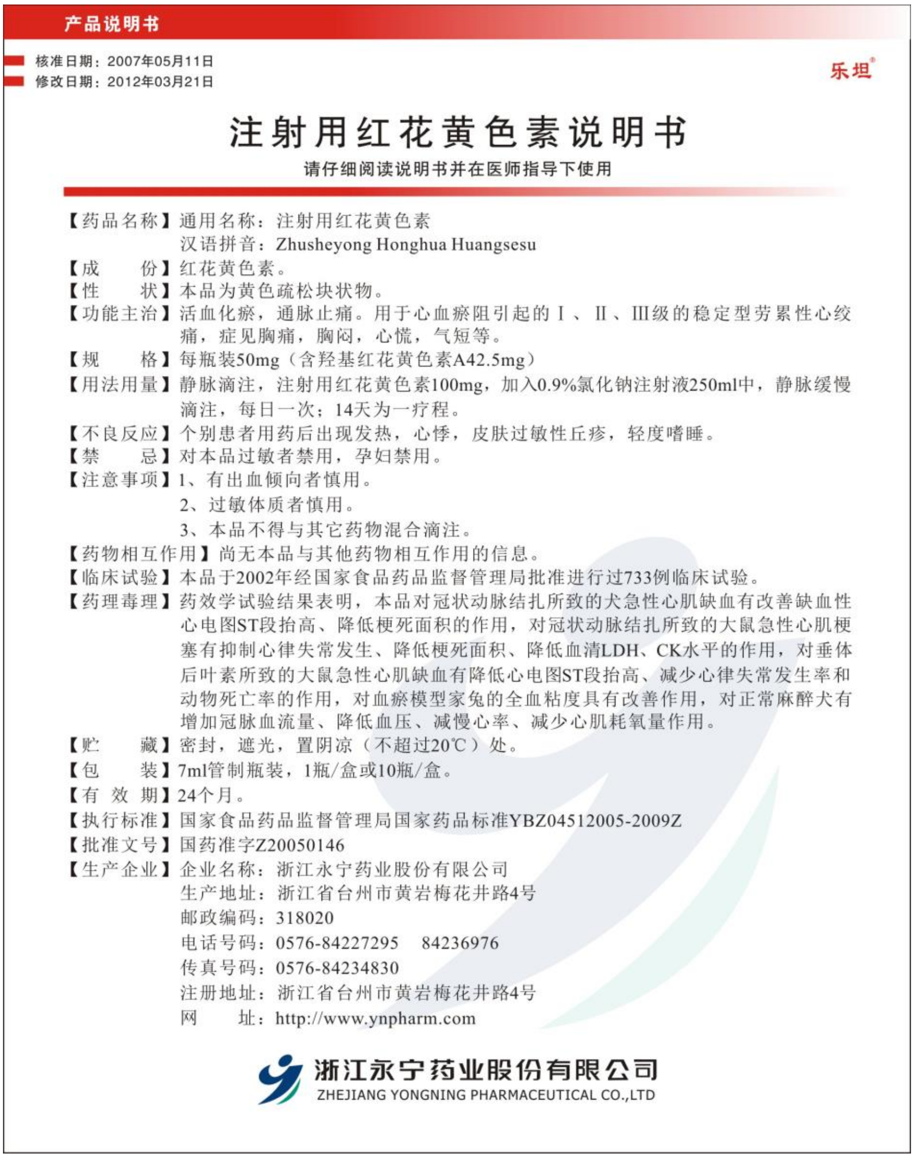

产品说明书

核准日期：2007年05月11日
修改日期：2012年03月21日

乐坦®

注射用红花黄色素说明书

请仔细阅读说明书并在医师指导下使用

【药品名称】通用名称：注射用红花黄色素
汉语拼音：Zhusheyong Honghua Huangsesu

【成　　份】红花黄色素。

【性　　状】本品为黄色疏松块状物。

【功能主治】活血化瘀，通脉止痛。用于心血瘀阻引起的Ⅰ、Ⅱ、Ⅲ级的稳定型劳累性心绞痛，症见胸痛，胸闷，心慌，气短等。

【规　　格】每瓶装50mg（含羟基红花黄色素A42.5mg）

【用法用量】静脉滴注，注射用红花黄色素100mg，加入0.9%氯化钠注射液250ml中，静脉缓慢滴注，每日一次；14天为一疗程。

【不良反应】个别患者用药后出现发热，心悸，皮肤过敏性丘疹，轻度嗜睡。

【禁　　忌】对本品过敏者禁用，孕妇禁用。

【注意事项】1、有出血倾向者慎用。
2、过敏体质者慎用。
3、本品不得与其它药物混合滴注。

【药物相互作用】尚无本品与其他药物相互作用的信息。

【临床试验】本品于2002年经国家食品药品监督管理局批准进行过733例临床试验。

【药理毒理】药效学试验结果表明，本品对冠状动脉结扎所致的犬急性心肌缺血有改善缺血性心电图ST段抬高、降低梗死面积的作用，对冠状动脉结扎所致的大鼠急性心肌梗塞有抑制心律失常发生、降低梗死面积、降低血清LDH、CK水平的作用，对垂体后叶素所致的大鼠急性心肌缺血有降低心电图ST段抬高、减少心律失常发生率和动物死亡率的作用，对血瘀模型家兔的全血粘度具有改善作用，对正常麻醉犬有增加冠脉血流量、降低血压、减慢心率、减少心肌耗氧量作用。

【贮　　藏】密封，遮光，置阴凉（不超过20℃）处。

【包　　装】7ml管制瓶装，1瓶/盒或10瓶/盒。

【有 效 期】24个月。

【执行标准】国家食品药品监督管理局国家药品标准YBZ04512005-2009Z

【批准文号】国药准字Z20050146

【生产企业】企业名称：浙江永宁药业股份有限公司
生产地址：浙江省台州市黄岩梅花井路4号
邮政编码：318020
电话号码：0576-84227295　84236976
传真号码：0576-84234830
注册地址：浙江省台州市黄岩梅花井路4号
网　　址：http://www.ynpharm.com

浙江永宁药业股份有限公司
ZHEJIANG YONGNING PHARMACEUTICAL CO.,LTD

Figure S14: Specification of *Safflower yellow* injection.

核准日期：2006 年 12 月 22 日
修订日期：2008 年 12 月 23 日
2013 年 01 月 24 日
2015 年 11 月 01 日

悦安欣®

苦碟子注射液说明书

请仔细阅读说明书并在医师指导下使用

警示语

本品应在临床监护下使用；
本品不良反应包括过敏性休克，应在有抢救条件的医疗机构使用，使用者应接受过过敏性休克抢救培训，用药后出现过敏反应或其他严重不良反应须立即停药并及时救治；
使用前如发现浑浊或沉淀不得使用；
本品不宜与其它药物混合使用；
对本品过敏者或过敏体质者禁用。

【药品名称】通用名称：苦碟子注射液
汉语拼音：Kudiezi Zhusheye

【成　　份】抱茎苦荬菜。

【性　　状】本品为浅黄棕色至黄棕色的澄明液体。

【功能主治】活血止痛、清热祛瘀。用于瘀血闭阻的胸痹，证见：胸闷、心痛、口苦，舌暗红或存瘀斑等。适用于冠心病、心绞痛见上述病状者。亦可用于脑梗塞者。

【规　　格】每支装 20ml

【用法用量】静脉滴注。一次 10–40 ml，一日 1 次；用 5% 葡萄糖或 0.9% 氯化钠注射液稀释至 250–500 ml 后应用。14 天为一疗程；或遵医嘱。

【不良反应】1. 过敏反应：皮肤潮红、皮疹、瘙痒、呼吸困难、憋气、心悸、紫绀、血压下降、过敏性休克等。罕见严重过敏反应，表现为呼吸困难、抽搐、过敏性休克等，极罕见曾使用过本品的患者再次使用时或在连续使用过程中出现迟发性严重过敏反应。
2. 全身性损害：畏寒、寒战、发热、乏力、多汗、疼痛、水肿等。
3. 呼吸系统：呼吸急促、憋气、咳嗽等。
4. 心血管系统：心悸、胸闷、心律失常、心区不适等。
5. 胃肠道系统：恶心、呕吐、腹痛、腹泻等。
6. 皮肤及其附件：风团样皮疹、丘疹、红斑疹、局部皮肤反应等。
7. 精神及神经系统：头晕、头痛、头胀、眩晕、抽搐、烦躁等。
8. 用药部位：注射部位的皮疹、疼痛、麻木等，有静脉炎的病例报告。
9. 其他：颤抖等。

【禁　　忌】1. 对本品或含有抱茎苦荬菜制剂过敏或有严重不良反应病史者禁用，过敏体质者禁用。
2. 严重肝肾损害、心衰及其他严重器质性病患者禁用。
3. 近期出血或有出血倾向者禁用。

【注意事项】1. 本品应在临床监护下使用，用药期间密切观察患者病情。
2. 本品不良反应包括过敏性休克，应在有抢救条件的医疗机构使用，使用者应接受过过敏性休克抢救培训，用药后出现过敏反应或其他严重不良反应须立即停药并及时救治。
3. 严格按照药品说明书规定的功能主治使用，禁止超功能主治用药。
4. 严格掌握用法用量。按照药品说明书推荐剂量及疗程使用药品。不超剂量、超浓度、过快滴注和长期连续用药。
5. 用药前应仔细询问患者情况、用药史和过敏史。肝肾功能异常患者、低血压患者、老人、哺乳期妇女、初次使用中药注射剂的患者应慎重使用，如确需使用请遵医嘱，并加强监测。
6. 静脉滴注时滴速以每分钟 40–60 滴为宜。
7. 高龄患者日使用量应不超过 20 ml，滴速以每分钟不超过 40 滴为宜。
8. 本品为中药注射剂，保存不当可能会影响药品质量；用药前和配制后及使用过程中应认真检查本品及滴注液，发现药液出现浑浊、沉淀、变色、结晶等药物性状改变以及瓶身有漏气、裂纹等现象时，均不得使用。
9. 本品应单独使用，禁忌与其他药品混合配伍使用。谨慎联合用药，如确需联合使用其他药品时，应谨慎考虑与本品的间隔时间以及药物相互作用等。
10. 目前尚无儿童应用本品的系统研究资料，不建议儿童使用。
11. 加强用药监护。用药过程中，应密切观察用药反应，特别是开始 30 分钟。发现异常，立即停药，采用积极救治措施，救治患者。

【药物相互作用】尚无本品与其他药物相互作用的信息。

【贮　　藏】密闭（10–30℃）。

【包　　装】玻璃安瓿包装，每盒 5 支。

【有 效 期】18 个月。

【执行标准】中成药地方标准上升国家药品标准部分（内科心系分册）WS–10354（ZD–0354）–2002

【批准文号】国药准字 Z20025449

【生产企业】企业名称：沈阳双鼎制药有限公司
生产地址：沈阳高新区浑南产业区高科路 15 号
邮政编码：110179
电话号码：024–23788988
传真号码：024–23788288
注册地址：沈阳市浑南新区高科路 15 号
网　　址：www. 双鼎制药 .com www.shuangding.com

Figure S15: Specification of *Kudiezi* injection.

核准日期：2006年07月18日
修改日期：2015年07月10日

大株红景天注射液说明书

请仔细阅读说明书并在医师指导下使用。
本品不良反应包括皮疹、瘙痒、寒战、发热、恶心等，偶见严重过敏反应，表现为过敏性休克等。
用药后出现过敏反应或其他严重不良反应须立即停药并及时救治。
老年人应遵照医嘱谨慎用药。
妊娠期妇女禁用。

【药品名称】
通用名称：大株红景天注射液
汉语拼音：Dazhu Hongjingtian Zhusheye
【成　　份】大株红景天
【性　　状】本品为淡黄色至棕黄色的澄明液体。
【功能主治】活血化瘀。用于治疗冠心病稳定型劳累性心绞痛，中医辨证为心血瘀阻证，症见：胸部刺痛，绞痛，固定不移，痛引肩背及臂内侧，胸闷，心悸不宁，唇舌紫暗，脉细涩。
【规　　格】每支装5ml。
【用法用量】静脉滴注。一次10ml．加入250ml的5%葡萄糖注射液中，一日1次。10天为一疗程。
【不良反应】
皮疹、瘙痒、寒战、发热、恶心等，偶见严重过敏反应，表现为过敏性休克等。
【禁　　忌】
1. 医护人员应在用药前仔细询问患者的过敏史，对使用该药品或含有大株红景天制剂曾发生过不良反应的患者、过敏体质的患者（包括对其他药品易产生过敏反应的患者）禁用。
2. 妊娠期妇女禁用。
【注意事项】
1. 本品不良反应包括过敏性休克，用药后出现过敏反应或其他严重不良反应须立即停药并及时救治。
2. 严格按照药品说明书规定的功能主治使用，禁止超功能主治用药。
3. 严格掌握用法用量及疗程，按照药品说明书推荐剂量使用药品，不超剂量使用。
4. 本品保存不当可能影响产品质量，应避免受冻和高温。用药前和配制后应认真检查本品及滴注液，发现药液出现浑浊、沉淀、变色、结晶等药物性状改变以及瓶身有漏气、裂纹等现象时，均不得使用。
5. 严禁混合配伍，谨慎联合用药。本品应单独使用，禁止与其他药品混合配伍使用。如确需联合使用其他药品时，应谨慎考虑与本品的间隔时间及药物相互作用等问题。
6. 老人、哺乳期妇女、肝肾功能异常者、初次使用中药注射剂者应慎重使用，如确需使用，应遵医嘱。特殊人群用药应加强监测。
7. 药品与稀释液配药后，应坚持即配即用，不宜长时间放置。静脉滴注时，必须稀释以后使用。严格控制滴注速度和用药剂量。用药过程中，应缓慢滴注，同时密切观察用药反应，特别是开始30分钟。发现异常，立即停药，采用积极救治措施，救治患者。
【孕妇及哺乳期妇女用药】哺乳期妇女应慎重使用。
【儿童用药】目前尚无儿童应用本品的系统研究资料，不建议儿童使用。
【老人用药】老年人应遵照医嘱谨慎用药。
【药物相互作用】因无与其它药物静脉混合滴注的经验，故不宜与其它药物混合滴注静脉给药。
【药理作用】药效学试验表明，本品可使冠脉结扎所致的急性心肌缺血犬的梗塞范围缩小、血清酶降低、缺血性心外膜电图改善；可使冠脉结扎再灌注心肌损伤大鼠的梗塞范围缩小，脂质过氧化水平降低；可使正常麻醉犬外周血管阻力和冠脉阻力降低，冠脉血流量增加，心肌耗氧量降低；可使花生四烯酸和胶原诱导的家兔血小板聚集率降低；可使大鼠全血粘度和血浆粘度降低。
长期毒性试验表明，大鼠连续5周腹腔注射本品（30，10g/kg），高剂量可使GOT升高，肝脏系数增大。犬连续 5 周静脉注射本品（12，3g/kg），未见明显毒性。
【药代动力学】尚不明确。
【贮　　藏】密封，避光。
【包　　装】低硼硅玻璃安瓿，2支/盒。
【有 效 期】18个月。
【执行标准】国家食品药品监督管理局药品注册批件，药品标准编号：YBZ11852006
【批准文号】国药准字Z20060361
【生产企业】
企业名称：通化玉圣药业有限公司
生产地址：吉林省通化市医药大路3998号
邮政编码：134008
电话号码：0432-63331111（销售）、0435-3945890（质量）
传真号码：0435-3945890
注册地址：吉林省通化市医药大路3998号
网　　址：www.jlthysyy.com

Figure S16: Specification of *Dazhuhongjingtian* injection.

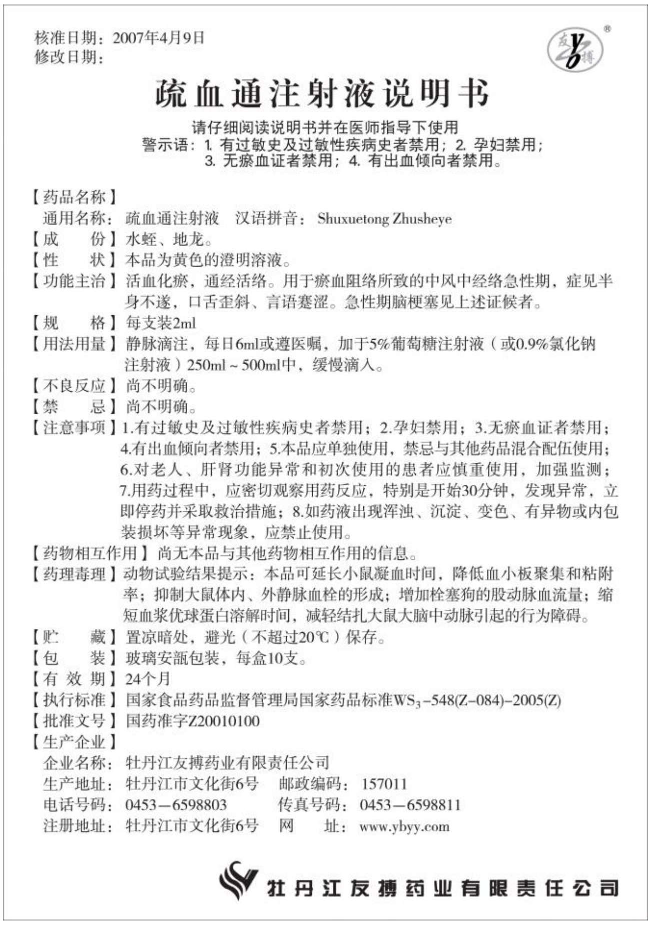

核准日期：2007年4月9日
修改日期：

疏血通注射液说明书

请仔细阅读说明书并在医师指导下使用
警示语：1. 有过敏史及过敏性疾病史者禁用；2. 孕妇禁用；
3. 无瘀血证者禁用；4. 有出血倾向者禁用。

【药品名称】
通用名称：疏血通注射液　汉语拼音：Shuxuetong Zhusheye
【成　　份】水蛭、地龙。
【性　　状】本品为黄色的澄明溶液。
【功能主治】活血化瘀，通经活络。用于瘀血阻络所致的中风中经络急性期，症见半身不遂，口舌歪斜、言语蹇涩。急性期脑梗塞见上述证候者。
【规　　格】每支装2ml
【用法用量】静脉滴注，每日6ml或遵医嘱，加于5%葡萄糖注射液（或0.9%氯化钠注射液）250ml～500ml中，缓慢滴入。
【不良反应】尚不明确。
【禁　　忌】尚不明确。
【注意事项】1.有过敏史及过敏性疾病史者禁用；2.孕妇禁用；3.无瘀血证者禁用；4.有出血倾向者禁用；5.本品应单独使用，禁忌与其他药品混合配伍使用；6.对老人、肝肾功能异常和初次使用的患者应慎重使用，加强监测；7.用药过程中，应密切观察用药反应，特别是开始30分钟，发现异常，立即停药并采取救治措施；8.如药液出现浑浊、沉淀、变色、有异物或内包装损坏等异常现象，应禁止使用。
【药物相互作用】尚无本品与其他药物相互作用的信息。
【药理毒理】动物试验结果提示：本品可延长小鼠凝血时间，降低血小板聚集和粘附率；抑制大鼠体内、外静脉血栓的形成；增加栓塞狗的股动脉血流量；缩短血浆优球蛋白溶解时间，减轻结扎大鼠大脑中动脉引起的行为障碍。
【贮　　藏】置凉暗处，避光（不超过20℃）保存。
【包　　装】玻璃安瓿包装，每盒10支。
【有 效 期】24个月
【执行标准】国家食品药品监督管理局国家药品标准WS_3-548(Z-084)-2005(Z)
【批准文号】国药准字Z20010100
【生产企业】
企业名称：牡丹江友搏药业有限责任公司
生产地址：牡丹江市文化街6号　邮政编码：157011
电话号码：0453－6598803　传真号码：0453－6598811
注册地址：牡丹江市文化街6号　网　址：www.ybyy.com

牡丹江友搏药业有限责任公司

Figure S17: Specification of *Shuxuetong* injection.

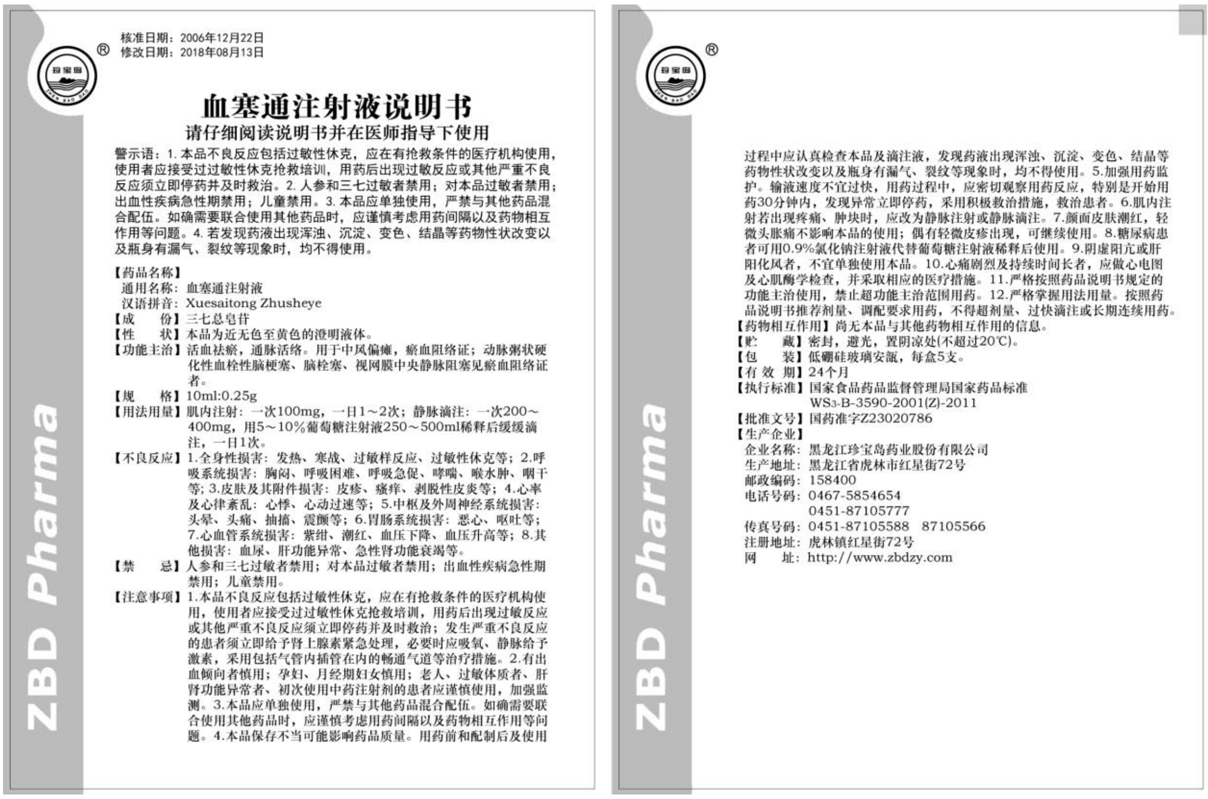

核准日期：2006年12月22日
修改日期：2018年08月13日

血塞通注射液说明书

请仔细阅读说明书并在医师指导下使用

警示语：1. 本品不良反应包括过敏性休克，应在有抢救条件的医疗机构使用，使用者应接受过过敏性休克抢救培训，用药后出现过敏反应或其他严重不良反应须立即停药并及时救治。2. 人参和三七过敏者禁用；对本品过敏者禁用；出血性疾病急性期禁用；儿童禁用。3. 本品应单独使用，严禁与其他药品混合配伍。如确需要联合使用其他药品时，应谨慎考虑用药间隔以及药物相互作用等问题。4. 若发现药液出现浑浊、沉淀、变色、结晶等药物性状改变以及瓶身有漏气、裂纹等现象时，均不得使用。

【药品名称】
通用名称：血塞通注射液
汉语拼音：Xuesaitong Zhusheye
【成　　份】三七总皂苷
【性　　状】本品为近无色至黄色的澄明液体。
【功能主治】活血祛瘀，通脉活络。用于中风偏瘫，瘀血阻络证；动脉粥状硬化性血栓性脑梗塞、脑栓塞、视网膜中央静脉阻塞见瘀血阻络证者。
【规　　格】10ml:0.25g
【用法用量】肌内注射：一次100mg，一日1～2次；静脉滴注：一次200～400mg，用5～10%葡萄糖注射液250～500ml稀释后缓缓滴注，一日1次。
【不良反应】1.全身性损害：发热、寒战、过敏样反应、过敏性休克等；2.呼吸系统损害：胸闷、呼吸困难、呼吸急促、哮喘、喉水肿、咽干等；3.皮肤及其附件损害：皮疹、瘙痒、剥脱性皮炎等；4.心率及心律紊乱：心悸、心动过速等；5.中枢及外周神经系统损害：头晕、头痛、抽搐、震颤等；6.胃肠系统损害：恶心、呕吐等；7.心血管系统损害：紫绀、潮红、血压下降、血压升高等；8.其他损害：血尿、肝功能异常、急性肾功能衰竭等。
【禁　　忌】人参和三七过敏者禁用；对本品过敏者禁用；出血性疾病急性期禁用；儿童禁用。
【注意事项】1.本品不良反应包括过敏性休克，应在有抢救条件的医疗机构使用，使用者应接受过过敏性休克抢救培训，用药后出现过敏反应或其他严重不良反应须立即停药并及时救治；发生严重不良反应的患者须立即给予肾上腺素紧急处理，必要时应吸氧、静脉给予激素，采用包括气管内插管在内的畅通气道等治疗措施。2.有出血倾向者慎用；孕妇、月经期妇女慎用；老人、过敏体质者、肝肾功能异常者、初次使用中药注射剂的患者应谨慎使用，加强监测。3.本品应单独使用，严禁与其他药品混合配伍。如确需要联合使用其他药品时，应谨慎考虑用药间隔以及药物相互作用等问题。4.本品保存不当可能影响药品质量。用药前和配制后及使用过程中应认真检查本品及滴注液，发现药液出现浑浊、沉淀、变色、结晶等药物性状改变以及瓶身有漏气、裂纹等现象时，均不得使用。5.加强用药监护。输液速度不宜过快，用药过程中，应密切观察用药反应，特别是开始用药30分钟内，发现异常立即停药，采用积极救治措施，救治患者。6.肌内注射若出现疼痛、肿块时，应改为静脉注射或静脉滴注。7.颜面皮肤潮红，轻微头胀痛不影响本品的使用；偶有轻微皮疹出现，可继续使用。8.糖尿病患者可用0.9%氯化钠注射液代替葡萄糖注射液稀释后使用。9.阴虚阳亢或肝阳化风者，不宜单独使用本品。10.心痛剧烈及持续时间长者，应做心电图及心肌酶学检查，并采取相应的医疗措施。11.严格按照药品说明书规定的功能主治使用，禁止超功能主治范围用药。12.严格掌握用法用量。按照药品说明书推荐剂量、调配要求用药，不得超剂量、过快滴注或长期连续用药。
【药物相互作用】尚无本品与其他药物相互作用的信息。
【贮　　藏】密封，避光，置阴凉处(不超过20℃)。
【包　　装】低硼硅玻璃安瓿，每盒5支。
【有 效 期】24个月
【执行标准】国家食品药品监督管理局国家药品标准
WS_3-B-3590-2001(Z)-2011
【批准文号】国药准字Z23020786
【生产企业】
企业名称：黑龙江珍宝岛药业股份有限公司
生产地址：黑龙江省虎林市红星街72号
邮政编码：158400
电话号码：0467-5854654
0451-87105777
传真号码：0451-87105588　87105566
注册地址：虎林镇红星街72号
网　　址：http://www.zbdzy.com

Figure S18: Specification of *Xuesaitong* injection.

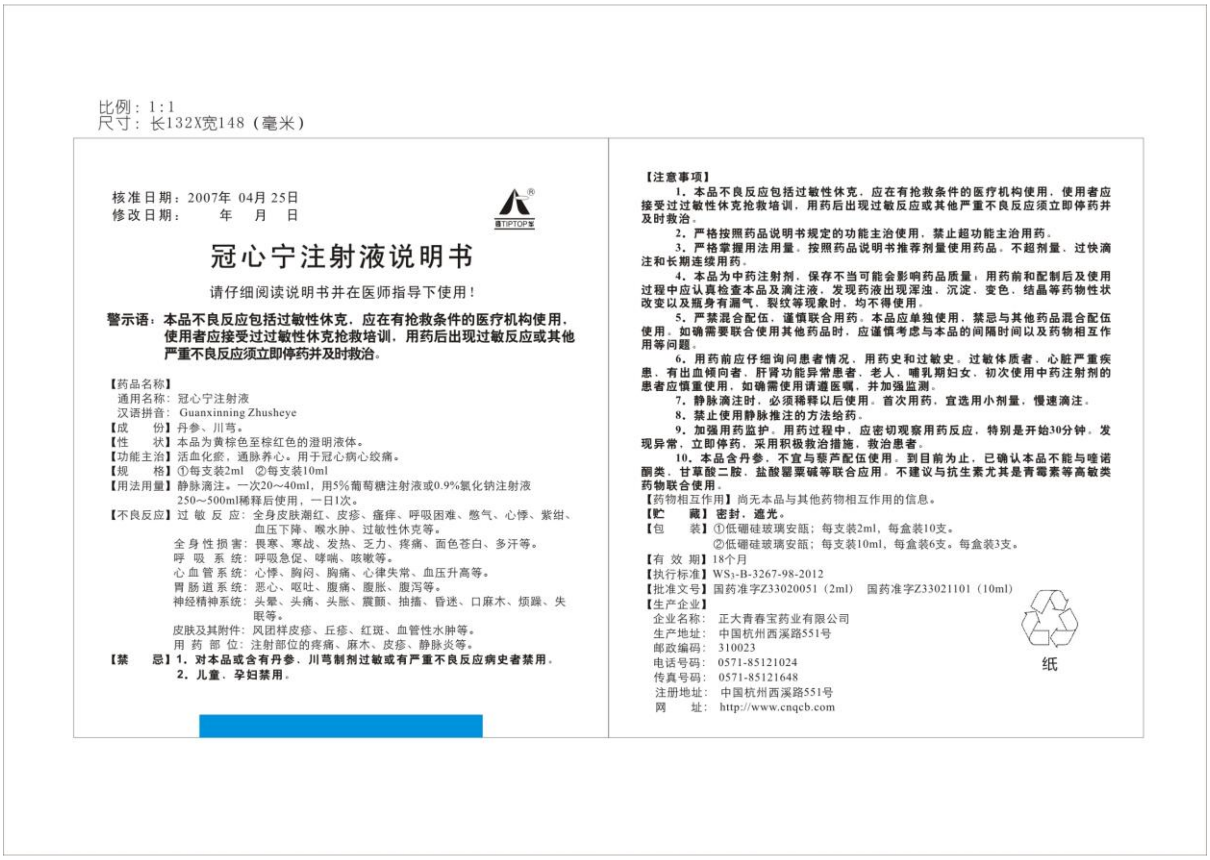
比例：1:1
尺寸：长132X宽148（毫米）

核准日期：2007年 04月 25日
修改日期：　　年　月　日

TIPTOP

冠心宁注射液说明书

请仔细阅读说明书并在医师指导下使用！

警示语：本品不良反应包括过敏性休克，应在有抢救条件的医疗机构使用，使用者应接受过过敏性休克抢救培训，用药后出现过敏反应或其他严重不良反应须立即停药并及时救治。

【药品名称】
通用名称：冠心宁注射液
汉语拼音：Guanxinning Zhusheye
【成　　份】丹参、川芎。
【性　　状】本品为黄棕色至棕红色的澄明液体。
【功能主治】活血化瘀，通脉养心。用于冠心病心绞痛。
【规　　格】①每支装2ml　②每支装10ml
【用法用量】静脉滴注。一次20～40ml，用5%葡萄糖注射液或0.9%氯化钠注射液250～500ml稀释后使用，一日1次。
【不良反应】过敏反应：全身皮肤潮红、皮疹、瘙痒、呼吸困难、憋气、心悸、紫绀、血压下降、喉水肿、过敏性休克等。
全身性损害：畏寒、寒战、发热、乏力、疼痛、面色苍白、多汗等。
呼吸系统：呼吸急促、哮喘、咳嗽等。
心血管系统：心悸、胸闷、胸痛、心律失常、血压升高等。
胃肠道系统：恶心、呕吐、腹痛、腹胀、腹泻等。
神经精神系统：头晕、头痛、头胀、震颤、抽搐、昏迷、口麻木、烦躁、失眠等。
皮肤及其附件：风团样皮疹、丘疹、红斑、血管性水肿等。
用药部位：注射部位的疼痛、麻木、皮疹、静脉炎等。
【禁　　忌】1．对本品或含有丹参、川芎制剂过敏或有严重不良反应病史者禁用。
2．儿童、孕妇禁用。

【注意事项】
1．本品不良反应包括过敏性休克，应在有抢救条件的医疗机构使用，使用者应接受过过敏性休克抢救培训，用药后出现过敏反应或其他严重不良反应须立即停药并及时救治。
2．严格按照药品说明书规定的功能主治使用，禁止超功能主治用药。
3．严格掌握用法用量。按照药品说明书推荐剂量使用药品。不超剂量、过快滴注和长期连续用药。
4．本品为中药注射剂，保存不当可能会影响药品质量；用药前和配制后及使用过程中应认真检查本品及滴注液，发现药液出现浑浊、沉淀、变色、结晶等药物性状改变以及瓶身有漏气、裂纹等现象时，均不得使用。
5．严禁混合配伍，谨慎联合用药。本品应单独使用，禁忌与其他药品混合配伍使用。如确需要联合使用其他药品时，应谨慎考虑与本品的间隔时间以及药物相互作用等问题。
6．用药前应仔细询问患者情况、用药史和过敏史。过敏体质者、心脏严重疾患、有出血倾向者、肝肾功能异常患者、老人、哺乳期妇女、初次使用中药注射剂的患者应慎重使用，如确需使用请遵医嘱，并加强监测。
7．静脉滴注时，必须稀释以后使用。首次用药，宜选用小剂量，慢速滴注。
8．禁止使用静脉推注的方法给药。
9．加强用药监护。用药过程中，应密切观察用药反应，特别是开始30分钟。发现异常，立即停药，采用积极救治措施，救治患者。
10．本品含丹参，不宜与藜芦配伍使用。到目前为止，已确认本品不能与喹诺酮类、甘草酸二胺、盐酸罂粟碱等联合应用。不建议与抗生素尤其是青霉素等高敏类药物联合使用。
【药物相互作用】尚无本品与其他药物相互作用的信息。
【贮　　藏】密封，遮光。
【包　　装】①低硼硅玻璃安瓿；每支装2ml，每盒装10支。
②低硼硅玻璃安瓿；每支装10ml，每盒装6支。每盒装3支。
【有 效 期】18个月
【执行标准】WS_3-B-3267-98-2012
【批准文号】国药准字Z33020051（2ml）　国药准字Z33021101（10ml）
【生产企业】
企业名称：正大青春宝药业有限公司
生产地址：中国杭州西溪路551号
邮政编码：310023
电话号码：0571-85121024
传真号码：0571-85121648
注册地址：中国杭州西溪路551号
网　　址：http://www.cnqcb.com

纸

Figure S19: Specification of *Guanxinning* injection.

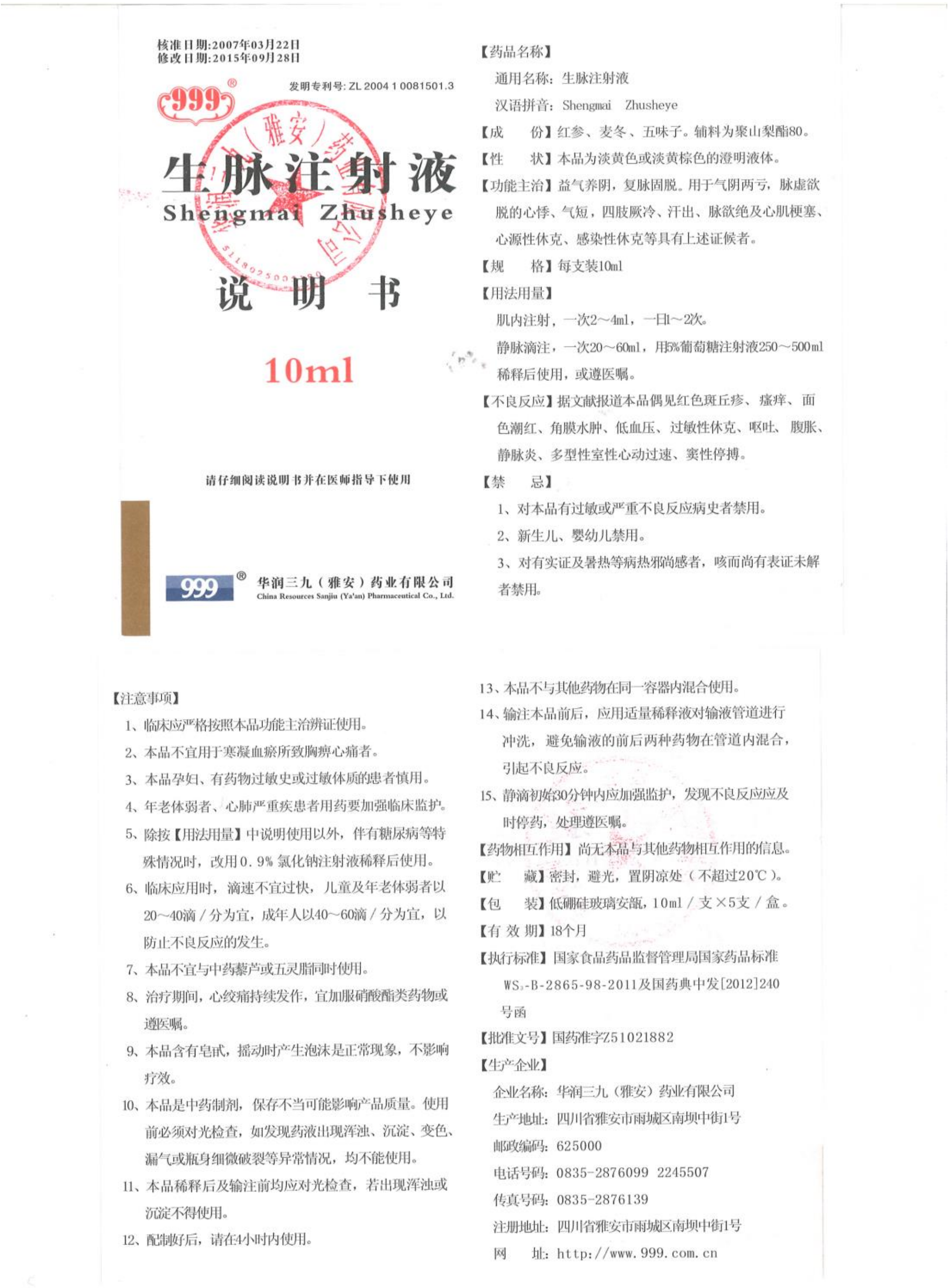
核准日期:2007年03月22日
修改日期:2015年09月28日

发明专利号: ZL 2004 1 0081501.3

999®

生脉注射液
Shengmai Zhusheye
说明书
10ml

请仔细阅读说明书并在医师指导下使用

999® 华润三九（雅安）药业有限公司
China Resources Sanjiu (Ya'an) Pharmaceutical Co., Ltd.

【药品名称】
通用名称：生脉注射液
汉语拼音：Shengmai Zhusheye
【成　　份】红参、麦冬、五味子。辅料为聚山梨酯80。
【性　　状】本品为淡黄色或淡黄棕色的澄明液体。
【功能主治】益气养阴，复脉固脱。用于气阴两亏，脉虚欲脱的心悸、气短，四肢厥冷、汗出、脉欲绝及心肌梗塞、心源性休克、感染性休克等具有上述证候者。
【规　　格】每支装10ml
【用法用量】
肌内注射，一次2～4ml，一日1～2次。
静脉滴注，一次20～60ml，用5%葡萄糖注射液250～500ml稀释后使用，或遵医嘱。
【不良反应】据文献报道本品偶见红色斑丘疹、瘙痒、面色潮红、角膜水肿、低血压、过敏性休克、呕吐、腹胀、静脉炎、多型性室性心动过速、窦性停搏。
【禁　　忌】
1、对本品有过敏或严重不良反应病史者禁用。
2、新生儿、婴幼儿禁用。
3、对有实证及暑热等病热邪尚感者，咳而尚有表证未解者禁用。

【注意事项】
1、临床应严格按照本品功能主治辨证使用。
2、本品不宜用于寒凝血瘀所致胸痹心痛者。
3、本品孕妇、有药物过敏史或过敏体质的患者慎用。
4、年老体弱者、心肺严重疾患者用药要加强临床监护。
5、除按【用法用量】中说明使用以外，伴有糖尿病等特殊情况时，改用0.9%氯化钠注射液稀释后使用。
6、临床应用时，滴速不宜过快，儿童及年老体弱者以20～40滴／分为宜，成年人以40～60滴／分为宜，以防止不良反应的发生。
7、本品不宜与中药藜芦或五灵脂同时使用。
8、治疗期间，心绞痛持续发作，宜加服硝酸酯类药物或遵医嘱。
9、本品含有皂甙，摇动时产生泡沫是正常现象，不影响疗效。
10、本品是中药制剂，保存不当可能影响产品质量。使用前必须对光检查，如发现药液出现浑浊、沉淀、变色、漏气或瓶身细微破裂等异常情况，均不能使用。
11、本品稀释后及输注前均应对光检查，若出现浑浊或沉淀不得使用。
12、配制好后，请在4小时内使用。
13、本品不与其他药物在同一容器内混合使用。
14、输注本品前后，应用适量稀释液对输液管道进行冲洗，避免输液的前后两种药物在管道内混合，引起不良反应。
15、静滴初始30分钟内应加强监护，发现不良反应应及时停药，处理遵医嘱。
【药物相互作用】尚无本品与其他药物相互作用的信息。
【贮　　藏】密封，避光，置阴凉处（不超过20℃）。
【包　　装】低硼硅玻璃安瓿，10ml／支×5支／盒。
【有 效 期】18个月
【执行标准】国家食品药品监督管理局国家药品标准WS_3-B-2865-98-2011及国药典中发[2012]240号函
【批准文号】国药准字Z51021882
【生产企业】
企业名称：华润三九（雅安）药业有限公司
生产地址：四川省雅安市雨城区南坝中街1号
邮政编码：625000
电话号码：0835-2876099 2245507
传真号码：0835-2876139
注册地址：四川省雅安市雨城区南坝中街1号
网　　址：http://www.999.com.cn

Figure S20: Specification of *Shengmai* injection.

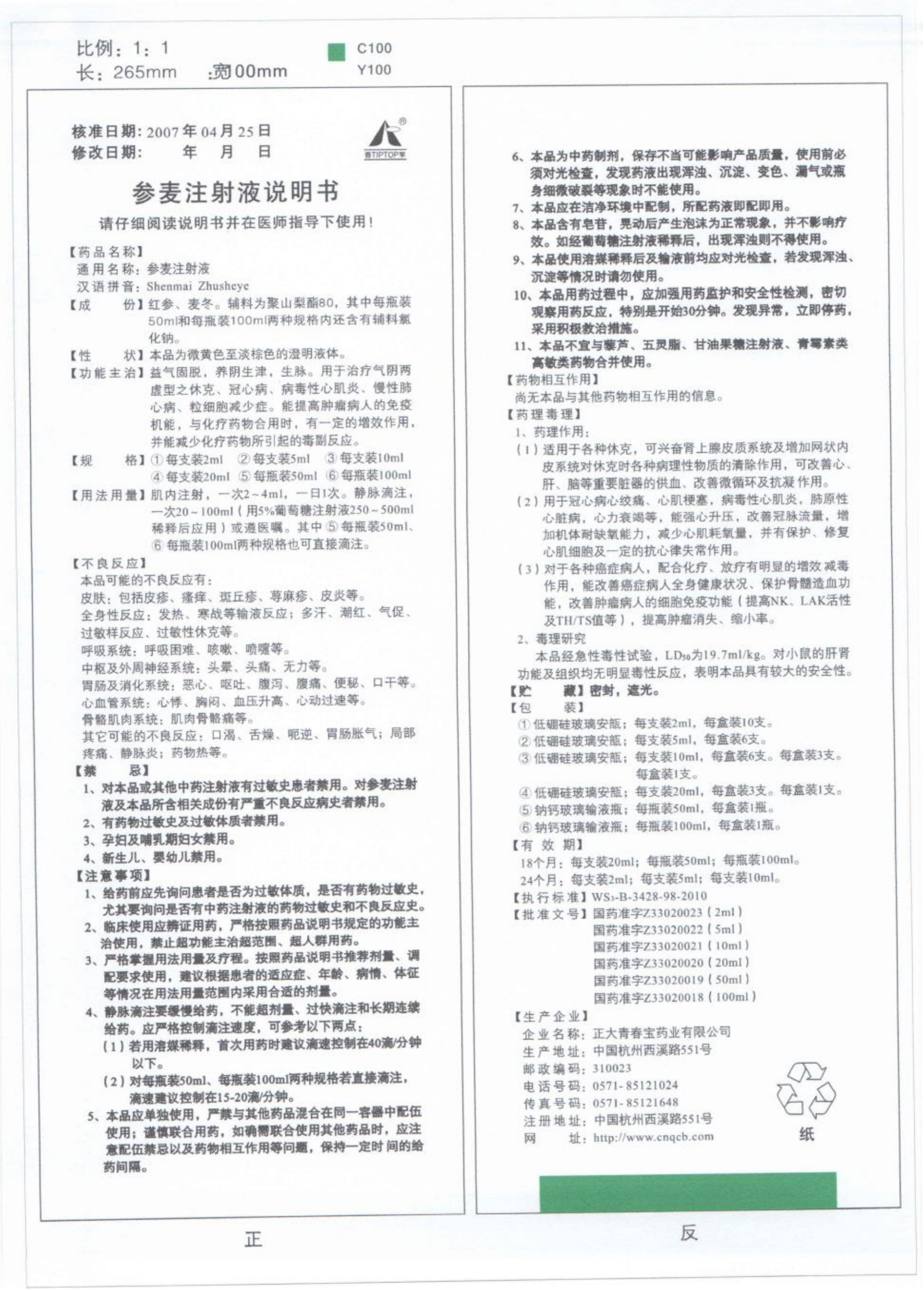

比例：1：1

长：265mm :宽00mm

C100

Y100

核准日期：2007年04月25日

修改日期： 年 月 日

TIPTOP

参麦注射液说明书

请仔细阅读说明书并在医师指导下使用！

【药品名称】

通用名称：参麦注射液

汉语拼音：Shenmai Zhusheye

【成　　份】红参、麦冬。辅料为聚山梨酯80，其中每瓶装50ml和每瓶装100ml两种规格内还含有辅料氯化钠。

【性　　状】本品为微黄色至淡棕色的澄明液体。

【功能主治】益气固脱，养阴生津，生脉。用于治疗气阴两虚型之休克、冠心病、病毒性心肌炎、慢性肺心病、粒细胞减少症。能提高肿瘤病人的免疫机能，与化疗药物合用时，有一定的增效作用，并能减少化疗药物所引起的毒副反应。

【规　　格】① 每支装2ml　② 每支装5ml　③ 每支装10ml　④ 每支装20ml　⑤ 每瓶装50ml　⑥ 每瓶装100ml

【用法用量】肌内注射，一次2～4ml，一日1次。静脉滴注，一次20～100ml（用5%葡萄糖注射液250～500ml稀释后应用）或遵医嘱。其中 ⑤ 每瓶装50ml、⑥ 每瓶装100ml两种规格也可直接滴注。

【不良反应】

本品可能的不良反应有：

皮肤：包括皮疹、瘙痒、斑丘疹、荨麻疹、皮炎等。

全身性反应：发热、寒战等输液反应；多汗、潮红、气促、过敏样反应、过敏性休克等。

呼吸系统：呼吸困难、咳嗽、喷嚏等。

中枢及外周神经系统：头晕、头痛、无力等。

胃肠及消化系统：恶心、呕吐、腹泻、腹痛、便秘、口干等。

心血管系统：心悸、胸闷、血压升高、心动过速等。

骨骼肌肉系统：肌肉骨骼痛等。

其它可能的不良反应：口渴、舌燥、呃逆、胃肠胀气；局部疼痛、静脉炎；药物热等。

【禁　　忌】

1、对本品或其他中药注射液有过敏史患者禁用。对参麦注射液及本品所含相关成份有严重不良反应病史者禁用。

2、有药物过敏史及过敏体质者禁用。

3、孕妇及哺乳期妇女禁用。

4、新生儿、婴幼儿禁用。

【注意事项】

1、给药前应先询问患者是否为过敏体质，是否有药物过敏史，尤其要询问是否有中药注射液的药物过敏史和不良反应史。

2、临床使用应辨证用药，严格按照药品说明书规定的功能主治使用，禁止超功能主治超范围、超人群用药。

3、严格掌握用法用量及疗程。按照药品说明书推荐剂量、调配要求使用，建议根据患者的适应症、年龄、病情、体征等情况在用法用量范围内采用合适的剂量。

4、静脉滴注要缓慢给药，不能超剂量、过快滴注和长期连续给药。应严格控制滴注速度，可参考以下两点：

（1）若用溶媒稀释，首次用药时建议滴速控制在40滴/分钟以下。

（2）对每瓶装50ml、每瓶装100ml两种规格若直接滴注，滴速建议控制在15-20滴/分钟。

5、本品应单独使用，严禁与其他药品混合在同一容器中配伍使用；谨慎联合用药，如确需联合使用其他药品时，应注意配伍禁忌以及药物相互作用等问题，保持一定时 间的给药间隔。

正

6、本品为中药制剂，保存不当可能影响产品质量，使用前必须对光检查，发现药液出现浑浊、沉淀、变色、漏气或瓶身细微破裂等现象时不能使用。

7、本品应在洁净环境中配制，所配药液即配即用。

8、本品含有皂苷，晃动后产生泡沫为正常现象，并不影响疗效。如经葡萄糖注射液稀释后，出现浑浊则不得使用。

9、本品使用溶媒稀释后及输液前均应对光检查，若发现浑浊、沉淀等情况时请勿使用。

10、本品用药过程中，应加强用药监护和安全性检测，密切观察用药反应，特别是开始30分钟。发现异常，立即停药，采用积极救治措施。

11、本品不宜与藜芦、五灵脂、甘油果糖注射液、青霉素类高敏类药物合并使用。

【药物相互作用】

尚无本品与其他药物相互作用的信息。

【药理毒理】

1、药理作用：

（1）适用于各种休克，可兴奋肾上腺皮质系统及增加网状内皮系统对休克时各种病理性物质的清除作用，可改善心、肝、脑等重要脏器的供血、改善微循环及抗凝 作用。

（2）用于冠心病心绞痛、心肌梗塞，病毒性心肌炎，肺原性心脏病，心力衰竭等，能强心升压，改善冠脉流量，增加机体耐缺氧能力，减少心肌耗氧量，并有保护、修复心肌细胞及一定的抗心律失常作用。

（3）对于各种癌症病人，配合化疗、放疗有明显的增效 减毒作用，能改善癌症病人全身健康状况、保护骨髓造血功能，改善肿瘤病人的细胞免疫功能（提高NK、LAK活性及TH/TS值等），提高肿瘤消失、缩小率。

2、毒理研究

本品经急性毒性试验，LD_{50}为19.7ml/kg。对小鼠的肝肾功能及组织均无明显毒性反应，表明本品具有较大的安全性。

【贮　　藏】密封，遮光。

【包　　装】

① 低硼硅玻璃安瓿；每支装2ml，每盒装10支。

② 低硼硅玻璃安瓿；每支装5ml，每盒装6支。

③ 低硼硅玻璃安瓿；每支装10ml，每盒装6支。每盒装3支。每盒装1支。

④ 低硼硅玻璃安瓿；每支装20ml，每盒装3支。每盒装1支。

⑤ 钠钙玻璃输液瓶；每瓶装50ml，每盒装1瓶。

⑥ 钠钙玻璃输液瓶；每瓶装100ml，每盒装1瓶。

【有 效 期】

18个月：每支装20ml；每瓶装50ml；每瓶装100ml。

24个月：每支装2ml；每支装5ml；每支装10ml。

【执行标准】WS_3-B-3428-98-2010

【批准文号】国药准字Z33020023（2ml）

国药准字Z33020022（5ml）

国药准字Z33020021（10ml）

国药准字Z33020020（20ml）

国药准字Z33020019（50ml）

国药准字Z33020018（100ml）

【生产企业】

企业名称：正大青春宝药业有限公司

生产地址：中国杭州西溪路551号

邮政编码：310023

电话号码：0571-85121024

传真号码：0571-85121648

注册地址：中国杭州西溪路551号

网　　址：http://www.cnqcb.com

纸

反

Figure S21: Specification of *Shenmai* injection.

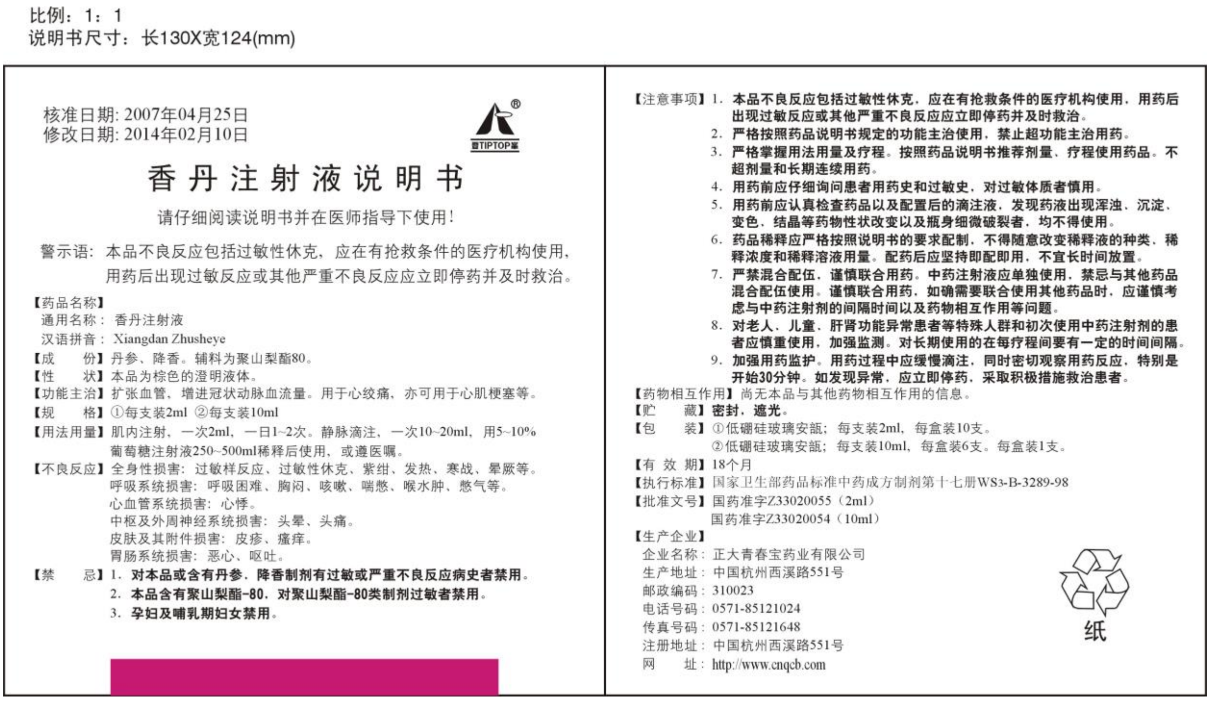

比例：1：1
说明书尺寸：长130X宽124(mm)

核准日期: 2007年04月25日
修改日期: 2014年02月10日

TIPTOP

香丹注射液说明书

请仔细阅读说明书并在医师指导下使用！

警示语：本品不良反应包括过敏性休克，应在有抢救条件的医疗机构使用，用药后出现过敏反应或其他严重不良反应应立即停药并及时救治。

【药品名称】
通用名称：香丹注射液
汉语拼音：Xiangdan Zhusheye
【成　　份】丹参、降香。辅料为聚山梨酯80。
【性　　状】本品为棕色的澄明液体。
【功能主治】扩张血管，增进冠状动脉血流量。用于心绞痛，亦可用于心肌梗塞等。
【规　　格】①每支装2ml ②每支装10ml
【用法用量】肌内注射，一次2ml，一日1~2次。静脉滴注，一次10~20ml，用5~10%葡萄糖注射液250~500ml稀释后使用，或遵医嘱。
【不良反应】全身性损害：过敏样反应、过敏性休克、紫绀、发热、寒战、晕厥等。
呼吸系统损害：呼吸困难、胸闷、咳嗽、喘憋、喉水肿、憋气等。
心血管系统损害：心悸。
中枢及外周神经系统损害：头晕、头痛。
皮肤及其附件损害：皮疹、瘙痒。
胃肠系统损害：恶心、呕吐。
【禁　　忌】1. **对本品或含有丹参，降香制剂有过敏或严重不良反应病史者禁用。**
2. **本品含有聚山梨酯-80，对聚山梨酯-80类制剂过敏者禁用。**
3. **孕妇及哺乳期妇女禁用。**

【注意事项】1. **本品不良反应包括过敏性休克，应在有抢救条件的医疗机构使用，用药后出现过敏反应或其他严重不良反应应立即停药并及时救治。**
2. **严格按照药品说明书规定的功能主治使用，禁止超功能主治用药。**
3. **严格掌握用法用量及疗程。按照药品说明书推荐剂量、疗程使用药品。不超剂量和长期连续用药。**
4. **用药前应仔细询问患者用药史和过敏史，对过敏体质者慎用。**
5. **用药前应认真检查药品以及配置后的滴注液，发现药液出现浑浊、沉淀、变色、结晶等药物性状改变以及瓶身细微破裂者，均不得使用。**
6. **药品稀释应严格按照说明书的要求配制，不得随意改变稀释液的种类、稀释浓度和稀释溶液用量。配药后应坚持即配即用，不宜长时间放置。**
7. **严禁混合配伍，谨慎联合用药。中药注射液应单独使用，禁忌与其他药品混合配伍使用。谨慎联合用药，如确需要联合使用其他药品时，应谨慎考虑与中药注射剂的间隔时间以及药物相互作用等问题。**
8. **对老人、儿童、肝肾功能异常患者等特殊人群和初次使用中药注射剂的患者应慎重使用，加强监测。对长期使用的在每疗程间要有一定的时间间隔。**
9. **加强用药监护。用药过程中应缓慢滴注，同时密切观察用药反应，特别是开始30分钟。如发现异常，应立即停药，采取积极措施救治患者。**
【药物相互作用】尚无本品与其他药物相互作用的信息。
【贮　　藏】**密封，遮光。**
【包　　装】①低硼硅玻璃安瓿；每支装2ml，每盒装10支。
②低硼硅玻璃安瓿；每支装10ml，每盒装6支。每盒装1支。
【有 效 期】18个月
【执行标准】国家卫生部药品标准中药成方制剂第十七册WS3-B-3289-98
【批准文号】国药准字Z33020055（2ml）
国药准字Z33020054（10ml）
【生产企业】
企业名称：正大青春宝药业有限公司
生产地址：中国杭州西溪路551号
邮政编码：310023
电话号码：0571-85121024
传真号码：0571-85121648
注册地址：中国杭州西溪路551号
网　　址：http://www.cnqcb.com

纸

Figure S22: Specification of *Xiangdan* injection.

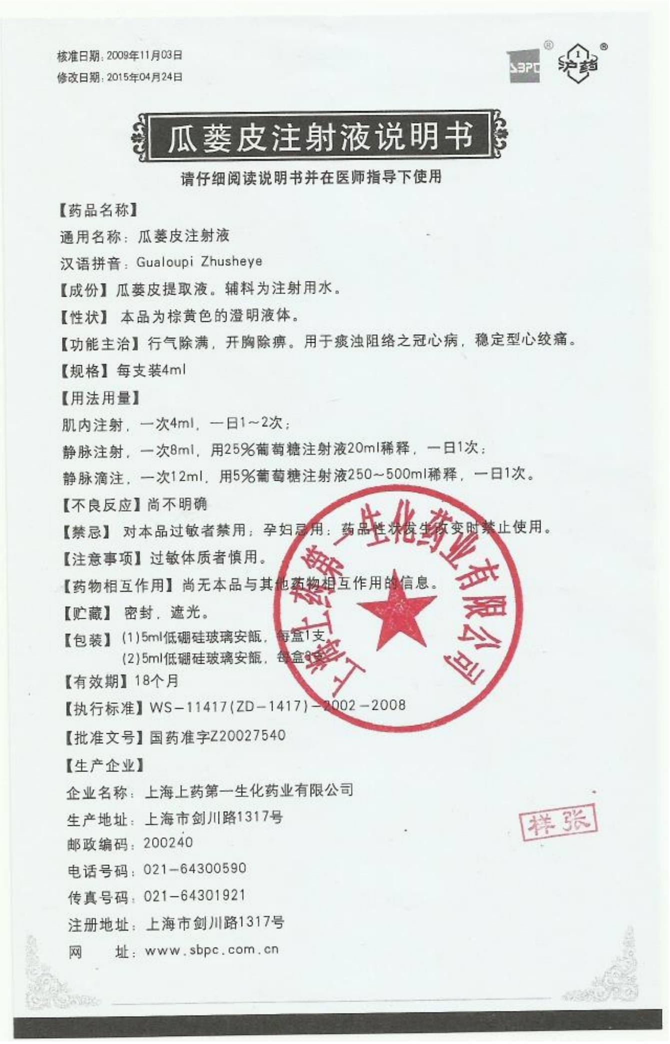

核准日期：2009年11月03日
修改日期：2015年04月24日

瓜蒌皮注射液说明书

请仔细阅读说明书并在医师指导下使用

【药品名称】
通用名称：瓜蒌皮注射液
汉语拼音：Gualoupi Zhusheye
【成份】瓜蒌皮提取液。辅料为注射用水。
【性状】本品为棕黄色的澄明液体。
【功能主治】行气除满，开胸除痹。用于痰浊阻络之冠心病，稳定型心绞痛。
【规格】每支装4ml
【用法用量】
肌内注射，一次4ml，一日1～2次；
静脉注射，一次8ml，用25%葡萄糖注射液20ml稀释，一日1次；
静脉滴注，一次12ml，用5%葡萄糖注射液250～500ml稀释，一日1次。
【不良反应】尚不明确
【禁忌】对本品过敏者禁用；孕妇忌用；药品性状发生改变时禁止使用。
【注意事项】过敏体质者慎用。
【药物相互作用】尚无本品与其他药物相互作用的信息。
【贮藏】密封，遮光。
【包装】(1)5ml低硼硅玻璃安瓿，每盒1支
(2)5ml低硼硅玻璃安瓿，每盒[illegible]
【有效期】18个月
【执行标准】WS-11417(ZD-1417)-2002-2008
【批准文号】国药准字Z20027540
【生产企业】
企业名称：上海上药第一生化药业有限公司
生产地址：上海市剑川路1317号
邮政编码：200240
电话号码：021-64300590
传真号码：021-64301921
注册地址：上海市剑川路1317号
网　　址：www.sbpc.com.cn

样张

Figure S23: Specification of *Gualoupi* injection

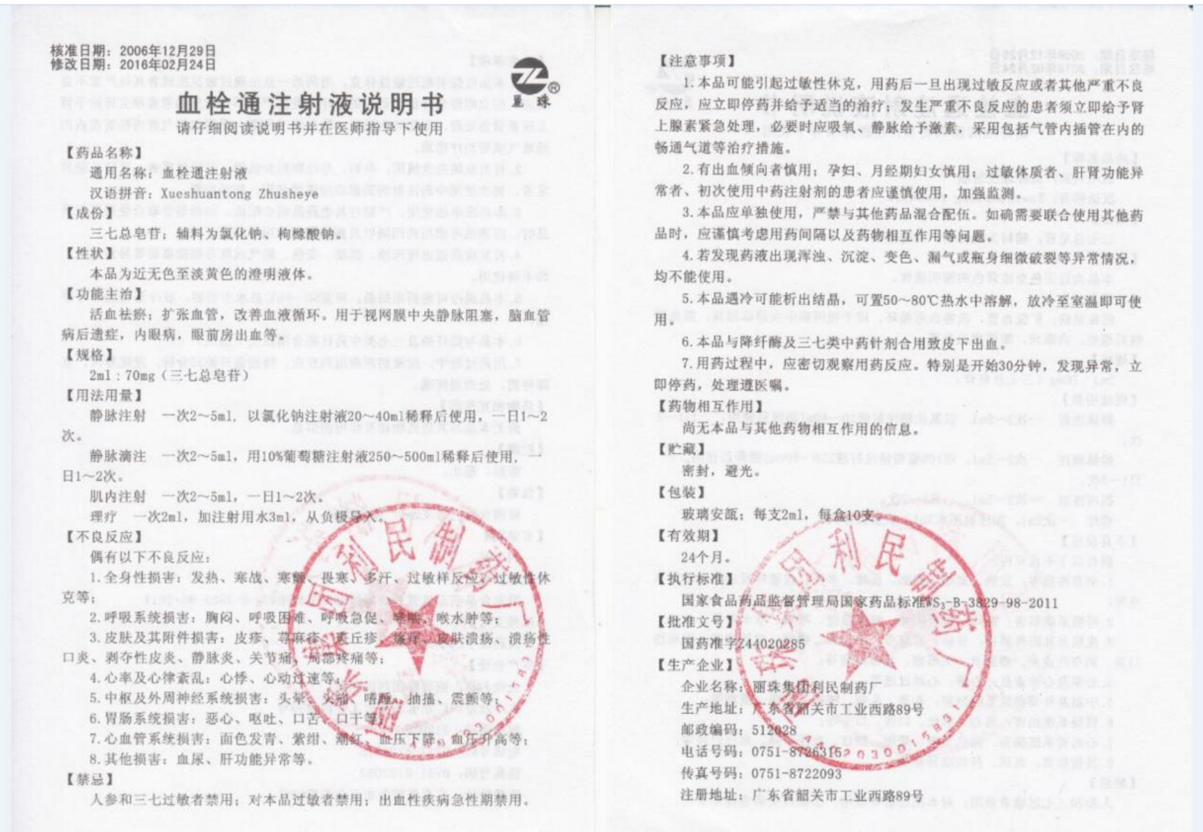

核准日期：2006年12月29日
修改日期：2016年02月24日

血栓通注射液说明书

请仔细阅读说明书并在医师指导下使用

【药品名称】

通用名称：血栓通注射液

汉语拼音：Xueshuantong Zhusheye

【成份】

三七总皂苷；辅料为氯化钠、枸橼酸钠。

【性状】

本品为近无色至淡黄色的澄明液体。

【功能主治】

活血祛瘀：扩张血管，改善血液循环。用于视网膜中央静脉阻塞，脑血管病后遗症，内眼病，眼前房出血等。

【规格】

2ml：70mg（三七总皂苷）

【用法用量】

静脉注射　一次2～5ml，以氯化钠注射液20～40ml稀释后使用，一日1～2次。

静脉滴注　一次2～5ml，用10%葡萄糖注射液250～500ml稀释后使用，一日1～2次。

肌内注射　一次2～5ml，一日1～2次。

理疗　一次2ml，加注射用水3ml，从负极导入。

【不良反应】

偶有以下不良反应：

1. 全身性损害：发热、寒战、寒颤、畏寒、多汗、过敏样反应、过敏性休克等；

2. 呼吸系统损害：胸闷、呼吸困难、呼吸急促、哮喘、喉水肿等；

3. 皮肤及其附件损害：皮疹、荨麻疹、斑丘疹、瘙痒、皮肤溃疡、溃疡性口炎、剥夺性皮炎、静脉炎、关节痛、局部疼痛等；

4. 心率及心律紊乱：心悸、心动过速等；

5. 中枢及外周神经系统损害：头晕、头痛、嗜睡、抽搐、震颤等；

6. 胃肠系统损害：恶心、呕吐、口苦、口干等；

7. 心血管系统损害：面色发青、紫绀、潮红、血压下降、血压升高等；

8. 其他损害：血尿、肝功能异常等。

【禁忌】

人参和三七过敏者禁用；对本品过敏者禁用；出血性疾病急性期禁用。

【注意事项】

1. 本品可能引起过敏性休克，用药后一旦出现过敏反应或者其他严重不良反应，应立即停药并给予适当的治疗；发生严重不良反应的患者须立即给予肾上腺素紧急处理，必要时应吸氧、静脉给予激素，采用包括气管内插管在内的畅通气道等治疗措施。

2. 有出血倾向者慎用；孕妇、月经期妇女慎用；过敏体质者、肝肾功能异常者、初次使用中药注射剂的患者应谨慎使用，加强监测。

3. 本品应单独使用，严禁与其他药品混合配伍。如确需要联合使用其他药品时，应谨慎考虑用药间隔以及药物相互作用等问题。

4. 若发现药液出现浑浊、沉淀、变色、漏气或瓶身细微破裂等异常情况，均不能使用。

5. 本品遇冷可能析出结晶，可置50～80℃热水中溶解，放冷至室温即可使用。

6. 本品与降纤酶及三七类中药针剂合用致皮下出血。

7. 用药过程中，应密切观察用药反应。特别是开始30分钟，发现异常，立即停药，处理遵医嘱。

【药物相互作用】

尚无本品与其他药物相互作用的信息。

【贮藏】

密封，避光。

【包装】

玻璃安瓿：每支2ml，每盒10支。

【有效期】

24个月。

【执行标准】

国家食品药品监督管理局国家药品标准WS_3-B-3829-98-2011

【批准文号】

国药准字Z44020285

【生产企业】

企业名称：丽珠集团利民制药厂

生产地址：广东省韶关市工业西路89号

邮政编码：512028

电话号码：0751-8726315

传真号码：0751-8722093

注册地址：广东省韶关市工业西路89号

Figure S24: Specification of *Xueshuantong* injection

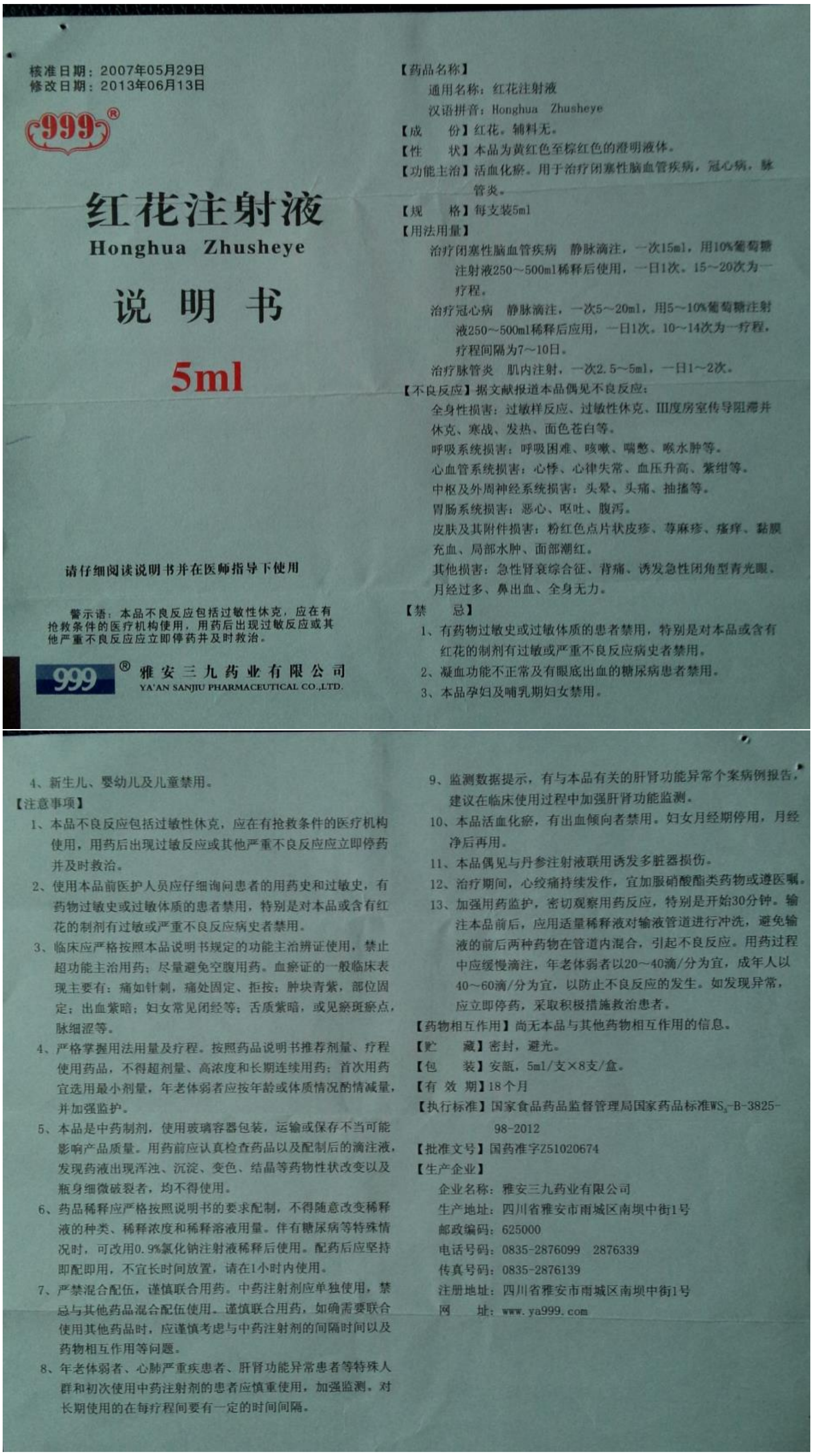

核准日期：2007年05月29日
修改日期：2013年06月13日

999®

红花注射液
Honghua Zhusheye
说 明 书
5ml

请仔细阅读说明书并在医师指导下使用

警示语：本品不良反应包括过敏性休克，应在有抢救条件的医疗机构使用，用药后出现过敏反应或其他严重不良反应应立即停药并及时救治。

999® 雅安三九药业有限公司
YA'AN SANJIU PHARMACEUTICAL CO.,LTD.

【药品名称】
通用名称：红花注射液
汉语拼音：Honghua Zhusheye
【成　　份】红花。辅料无。
【性　　状】本品为黄红色至棕红色的澄明液体。
【功能主治】活血化瘀。用于治疗闭塞性脑血管疾病，冠心病，脉管炎。
【规　　格】每支装5ml
【用法用量】
治疗闭塞性脑血管疾病　静脉滴注，一次15ml，用10%葡萄糖注射液250～500ml稀释后使用，一日1次。15～20次为一疗程。
治疗冠心病　静脉滴注，一次5～20ml，用5～10%葡萄糖注射液250～500ml稀释后应用，一日1次。10～14次为一疗程，疗程间隔为7～10日。
治疗脉管炎　肌内注射，一次2.5～5ml，一日1～2次。
【不良反应】据文献报道本品偶见不良反应：
全身性损害：过敏样反应、过敏性休克、III度房室传导阻滞并休克、寒战、发热、面色苍白等。
呼吸系统损害：呼吸困难、咳嗽、喘憋、喉水肿等。
心血管系统损害：心悸、心律失常、血压升高、紫绀等。
中枢及外周神经系统损害：头晕、头痛、抽搐等。
胃肠系统损害：恶心、呕吐、腹泻。
皮肤及其附件损害：粉红色点片状皮疹、荨麻疹、瘙痒、黏膜充血、局部水肿、面部潮红。
其他损害：急性肾衰综合征、背痛、诱发急性闭角型青光眼、月经过多、鼻出血、全身无力。
【禁　　忌】
1、有药物过敏史或过敏体质的患者禁用，特别是对本品或含有红花的制剂有过敏或严重不良反应病史者禁用。
2、凝血功能不正常及有眼底出血的糖尿病患者禁用。
3、本品孕妇及哺乳期妇女禁用。
4、新生儿、婴幼儿及儿童禁用。
【注意事项】
1、本品不良反应包括过敏性休克，应在有抢救条件的医疗机构使用，用药后出现过敏反应或其他严重不良反应应立即停药并及时救治。
2、使用本品前医护人员应仔细询问患者的用药史和过敏史，有药物过敏史或过敏体质的患者禁用，特别是对本品或含有红花的制剂有过敏或严重不良反应病史者禁用。
3、临床应严格按照本品说明书规定的功能主治辨证使用，禁止超功能主治用药；尽量避免空腹用药。血瘀证的一般临床表现主要有：痛如针刺，痛处固定、拒按；肿块青紫，部位固定；出血紫暗；妇女常见闭经等；舌质紫暗，或见瘀斑瘀点，脉细涩等。
4、严格掌握用法用量及疗程。按照药品说明书推荐剂量、疗程使用药品，不得超剂量、高浓度和长期连续用药；首次用药宜选用最小剂量，年老体弱者应按年龄或体质情况酌情减量，并加强监护。
5、本品是中药制剂，使用玻璃容器包装，运输或保存不当可能影响产品质量。用药前应认真检查药品以及配制后的滴注液，发现药液出现浑浊、沉淀、变色、结晶等药物性状改变以及瓶身细微破裂者，均不得使用。
6、药品稀释应严格按照说明书的要求配制，不得随意改变稀释液的种类、稀释浓度和稀释溶液用量。伴有糖尿病等特殊情况时，可改用0.9%氯化钠注射液稀释后使用。配药后应坚持即配即用，不宜长时间放置，请在1小时内使用。
7、严禁混合配伍，谨慎联合用药。中药注射剂应单独使用，禁忌与其他药品混合配伍使用。谨慎联合用药，如确需要联合使用其他药品时，应谨慎考虑与中药注射剂的间隔时间以及药物相互作用等问题。
8、年老体弱者、心肺严重疾患者、肝肾功能异常患者等特殊人群和初次使用中药注射剂的患者应慎重使用，加强监测。对长期使用的在每疗程间要有一定的时间间隔。
9、监测数据提示，有与本品有关的肝肾功能异常个案病例报告，建议在临床使用过程中加强肝肾功能监测。
10、本品活血化瘀，有出血倾向者禁用。妇女月经期停用，月经净后再用。
11、本品偶见与丹参注射液联用诱发多脏器损伤。
12、治疗期间，心绞痛持续发作，宜加服硝酸酯类药物或遵医嘱。
13、加强用药监护，密切观察用药反应，特别是开始30分钟。输注本品前后，应用适量稀释液对输液管道进行冲洗，避免输液的前后两种药物在管道内混合，引起不良反应。用药过程中应缓慢滴注，年老体弱者以20～40滴/分为宜，成年人以40～60滴/分为宜，以防止不良反应的发生。如发现异常，应立即停药，采取积极措施救治患者。
【药物相互作用】尚无本品与其他药物相互作用的信息。
【贮　　藏】密封，避光。
【包　　装】安瓿，5ml/支×8支/盒。
【有 效 期】18个月
【执行标准】国家食品药品监督管理局国家药品标准WS_3-B-3825-98-2012
【批准文号】国药准字Z51020674
【生产企业】
企业名称：雅安三九药业有限公司
生产地址：四川省雅安市雨城区南坝中街1号
邮政编码：625000
电话号码：0835-2876099　2876339
传真号码：0835-2876139
注册地址：四川省雅安市雨城区南坝中街1号
网　　址：www.ya999.com

Figure S25: Specification of *Safflower* injection.

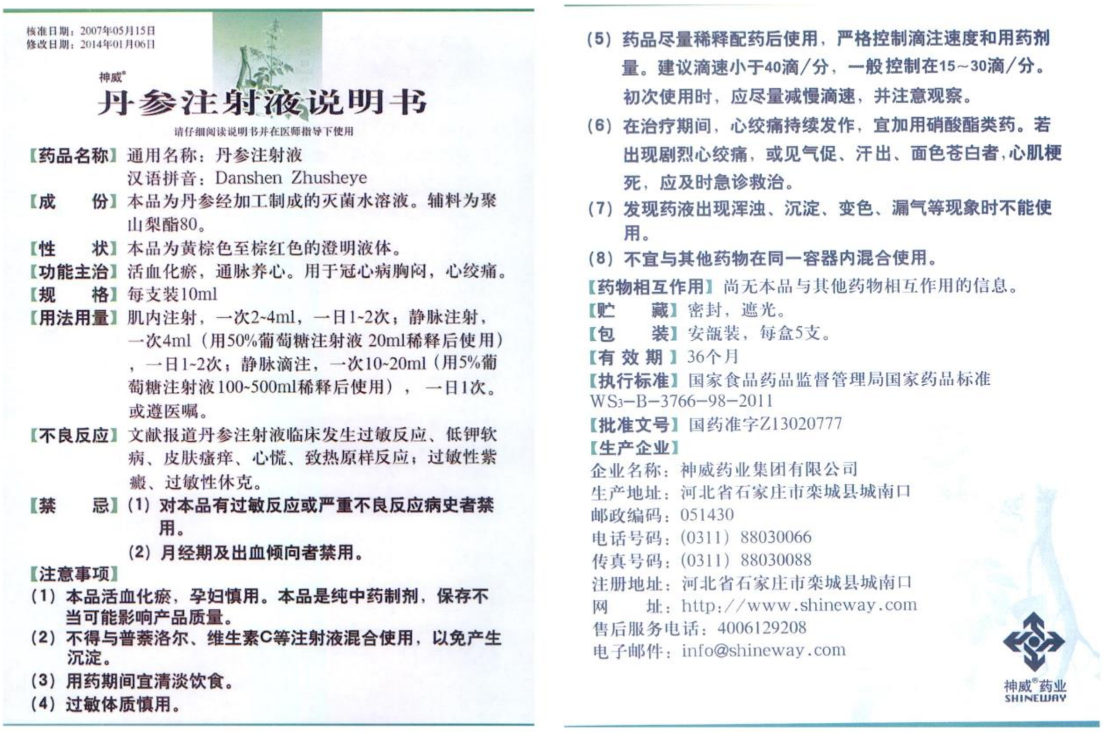

核准日期：2007年05月15日
修改日期：2014年01月06日

神威®

丹参注射液说明书

请仔细阅读说明书并在医师指导下使用

【药品名称】通用名称：丹参注射液
汉语拼音：Danshen Zhusheye
【成　　份】本品为丹参经加工制成的灭菌水溶液。辅料为聚山梨酯80。
【性　　状】本品为黄棕色至棕红色的澄明液体。
【功能主治】活血化瘀，通脉养心。用于冠心病胸闷，心绞痛。
【规　　格】每支装10ml
【用法用量】肌内注射，一次2~4ml，一日1~2次；静脉注射，一次4ml（用50%葡萄糖注射液 20ml稀释后使用），一日1~2次；静脉滴注，一次10~20ml（用5%葡萄糖注射液100~500ml稀释后使用），一日1次。或遵医嘱。
【不良反应】文献报道丹参注射液临床发生过敏反应、低钾软病、皮肤瘙痒、心慌、致热原样反应；过敏性紫癜、过敏性休克。
【禁　　忌】（1）对本品有过敏反应或严重不良反应病史者禁用。
（2）月经期及出血倾向者禁用。
【注意事项】
（1）本品活血化瘀，孕妇慎用。本品是纯中药制剂，保存不当可能影响产品质量。
（2）不得与普萘洛尔、维生素C等注射液混合使用，以免产生沉淀。
（3）用药期间宜清淡饮食。
（4）过敏体质慎用。
（5）药品尽量稀释配药后使用，严格控制滴注速度和用药剂量。建议滴速小于40滴/分，一般控制在15~30滴/分。初次使用时，应尽量减慢滴速，并注意观察。
（6）在治疗期间，心绞痛持续发作，宜加用硝酸酯类药。若出现剧烈心绞痛，或见气促、汗出、面色苍白者，心肌梗死，应及时急诊救治。
（7）发现药液出现浑浊、沉淀、变色、漏气等现象时不能使用。
（8）不宜与其他药物在同一容器内混合使用。
【药物相互作用】尚无本品与其他药物相互作用的信息。
【贮　　藏】密封，遮光。
【包　　装】安瓿装，每盒5支。
【有 效 期】36个月
【执行标准】国家食品药品监督管理局国家药品标准 WS_3-B-3766-98-2011
【批准文号】国药准字Z13020777
【生产企业】
企业名称：神威药业集团有限公司
生产地址：河北省石家庄市栾城县城南口
邮政编码：051430
电话号码：(0311) 88030066
传真号码：(0311) 88030088
注册地址：河北省石家庄市栾城县城南口
网　　址：http://www.shineway.com
售后服务电话：4006129208
电子邮件：info@shineway.com

Figure S26: Specification of *Danshen* injection.

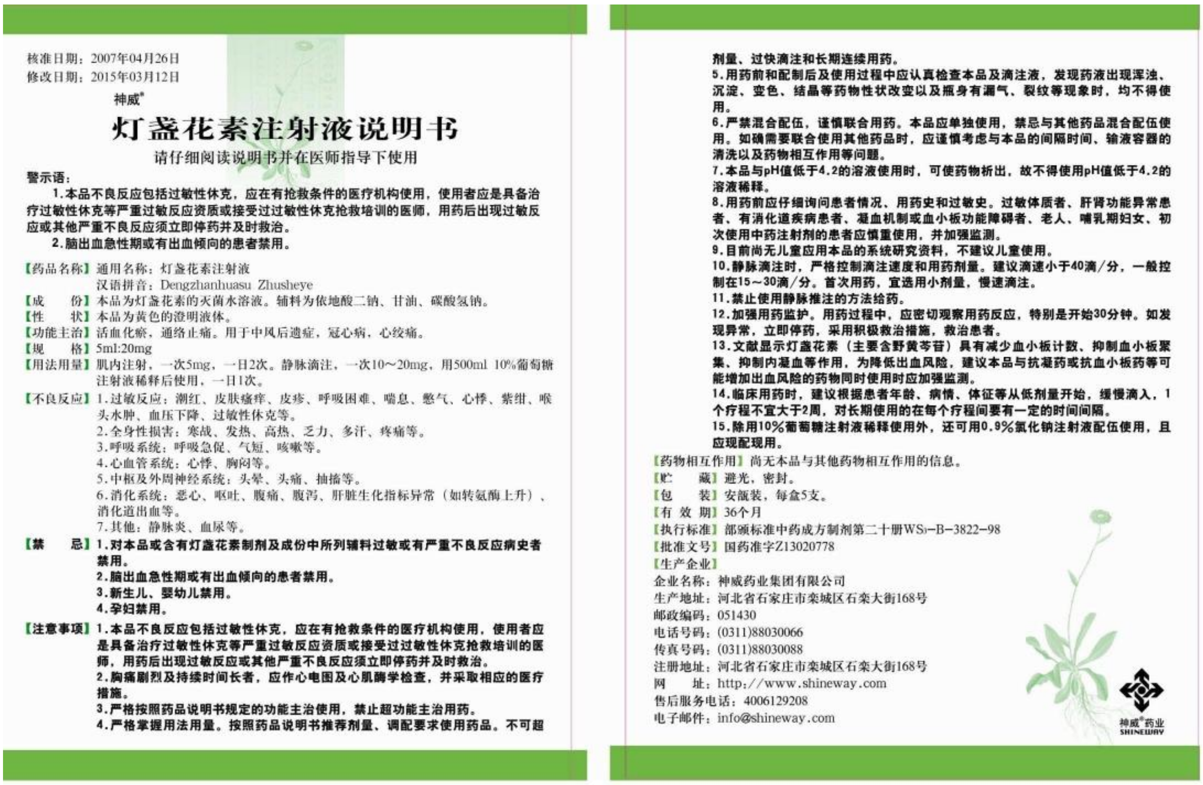

核准日期：2007年04月26日
修改日期：2015年03月12日

神威®

灯盏花素注射液说明书

请仔细阅读说明书并在医师指导下使用

警示语：
1.本品不良反应包括过敏性休克，应在有抢救条件的医疗机构使用，使用者应是具备治疗过敏性休克等严重过敏反应资质或接受过过敏性休克抢救培训的医师，用药后出现过敏反应或其他严重不良反应须立即停药并及时救治。
2.脑出血急性期或有出血倾向的患者禁用。

【药品名称】通用名称：灯盏花素注射液
汉语拼音：Dengzhanhuasu Zhusheye
【成　　份】本品为灯盏花素的灭菌水溶液。辅料为依地酸二钠、甘油、碳酸氢钠。
【性　　状】本品为黄色的澄明液体。
【功能主治】活血化瘀，通络止痛。用于中风后遗症，冠心病，心绞痛。
【规　　格】5ml:20mg
【用法用量】肌内注射，一次5mg，一日2次。静脉滴注，一次10～20mg，用500ml 10%葡萄糖注射液稀释后使用，一日1次。
【不良反应】1.过敏反应：潮红、皮肤瘙痒、皮疹、呼吸困难、喘息、憋气、心悸、紫绀、喉头水肿、血压下降、过敏性休克等。
2.全身性损害：寒战、发热、高热、乏力、多汗、疼痛等。
3.呼吸系统：呼吸急促、气短、咳嗽等。
4.心血管系统：心悸、胸闷等。
5.中枢及外周神经系统：头晕、头痛、抽搐等。
6.消化系统：恶心、呕吐、腹痛、腹泻、肝脏生化指标异常（如转氨酶上升）、消化道出血等。
7.其他：静脉炎、血尿等。
【禁　　忌】1.对本品或含有灯盏花素制剂及成份中所列辅料过敏或有严重不良反应病史者禁用。
2.脑出血急性期或有出血倾向的患者禁用。
3.新生儿、婴幼儿禁用。
4.孕妇禁用。
【注意事项】1.本品不良反应包括过敏性休克，应在有抢救条件的医疗机构使用，使用者应是具备治疗过敏性休克等严重过敏反应资质或接受过过敏性休克抢救培训的医师，用药后出现过敏反应或其他严重不良反应须立即停药并及时救治。
2.胸痛剧烈及持续时间长者，应作心电图及心肌酶学检查，并采取相应的医疗措施。
3.严格按照药品说明书规定的功能主治使用，禁止超功能主治用药。
4.严格掌握用法用量。按照药品说明书推荐剂量、调配要求使用药品。不可超剂量、过快滴注和长期连续用药。
5.用药前和配制后及使用过程中应认真检查本品及滴注液，发现药液出现浑浊、沉淀、变色、结晶等药物性状改变以及瓶身有漏气、裂纹等现象时，均不得使用。
6.严禁混合配伍，谨慎联合用药。本品应单独使用，禁忌与其他药品混合配伍使用。如确需要联合使用其他药品时，应谨慎考虑与本品的间隔时间、输液容器的清洗以及药物相互作用等问题。
7.本品与pH值低于4.2的溶液使用时，可使药物析出，故不得使用pH值低于4.2的溶液稀释。
8.用药前应仔细询问患者情况、用药史和过敏史。过敏体质者、肝肾功能异常患者、有消化道疾病患者、凝血机制或血小板功能障碍者、老人、哺乳期妇女、初次使用中药注射剂的患者应慎重使用，并加强监测。
9.目前尚无儿童应用本品的系统研究资料，不建议儿童使用。
10.静脉滴注时，严格控制滴注速度和用药剂量。建议滴速小于40滴/分，一般控制在15～30滴/分。首次用药，宜选用小剂量，慢速滴注。
11.禁止使用静脉推注的方法给药。
12.加强用药监护。用药过程中，应密切观察用药反应，特别是开始30分钟。如发现异常，立即停药，采用积极救治措施，救治患者。
13.文献显示灯盏花素（主要含野黄芩苷）具有减少血小板计数、抑制血小板聚集、抑制内凝血等作用，为降低出血风险，建议本品与抗凝药或抗血小板药等可能增加出血风险的药物同时使用时应加强监测。
14.临床用药时，建议根据患者年龄、病情、体征等从低剂量开始，缓慢滴入，1个疗程不宜大于2周，对长期使用的在每个疗程间要有一定的时间间隔。
15.除用10%葡萄糖注射液稀释使用外，还可用0.9%氯化钠注射液配伍使用，且应现配现用。
【药物相互作用】尚无本品与其他药物相互作用的信息。
【贮　　藏】避光，密封。
【包　　装】安瓿装，每盒5支。
【有 效 期】36个月
【执行标准】部颁标准中药成方制剂第二十册 WS_3-B-3822-98
【批准文号】国药准字Z13020778
【生产企业】
企业名称：神威药业集团有限公司
生产地址：河北省石家庄市栾城区石栾大街168号
邮政编码：051430
电话号码：(0311)88030066
传真号码：(0311)88030088
注册地址：河北省石家庄市栾城区石栾大街168号
网　　址：http://www.shineway.com
售后服务电话：4006129208
电子邮件：info@shineway.com

Figure S27: Specification of *Dengzhanhuasu* injection.

核准日期：2014年10月09日
修改日期：2014年10月09日

注射用益气复脉(冻干)说明书

请仔细阅读说明书并在医师指导下使用

样品

【药品名称】
通用名称：注射用益气复脉(冻干)
汉语拼音：Zhusheyong Yiqifumai

【成　　份】红参、麦冬、五味子，辅料为葡甲胺，甘露醇。

【性　　状】本品为浅黄色的疏松块状物；有引湿性。取1瓶内容物，加水2～3ml溶解后，为棕红色澄明液体。

【功能主治】益气复脉，养阴生津。用于冠心病劳累型心绞痛气阴两虚证，症见胸痹心痛，心悸气短、倦怠懒言、头晕目眩、面色少华、舌淡、少苔或剥苔，脉细弱或结代；冠心病所致慢性左心功能不全II、III级气阴两虚证，症见心悸、气短甚则气急喘促，胸闷隐痛，时作时止，倦怠乏力，面色苍白，动则汗出，舌淡、少苔或剥苔，脉细弱或结代。

【规　　格】每瓶装0.65g(相当于含红参0.5g、五味子0.75g和麦冬1.5g)

【用法用量】静脉滴注。每日1次，每次8瓶，用250ml～500ml 5%葡萄糖注射液或生理盐水稀释后静脉滴注。每分钟约40滴。疗程2周。

【不良反应】
1、临床研究期间偶见女性患者，用药过程中月经量明显增多。
2、临床研究期间偶见输液过程中出现针刺部位胀，减慢静滴速度后好转。
3、使用本品罕见皮疹、寒战、发热。若出现以上情况，请即刻停药并对症处理。

【禁　　忌】过敏体质者禁用。

【注意事项】
1、该药在输液过程中，液体应经过过滤器；若发现有气泡，应减慢滴速。
2、使用【用法用量】项下指定的溶剂充分溶解。使用稀释液稀释后，若发生浑浊、沉淀时请勿使用。
3、本品不得与其他药物混合注射使用。联合使用其他药品时，应谨慎考虑与本品的时间间隔以及药物相互作用等。

【孕妇及哺乳期妇女用药】尚无系统临床研究资料，应慎用。

【儿童用药】尚无系统临床研究资料，应慎用。

【药理毒理】药理作用：药效学实验表明，本品可使戊巴比妥钠所致的急性心力衰竭犬的左室内压升高，左室舒张末压降低，使dp/dt_{max}增加、$R\text{-}dp/dt_{max}$缩短，使心输出量和血压升高。本品可使失血性休克犬降低的左室内压和左室内压变化最大速率升高，使增加的左室舒张末压和中心静脉压降低。本品可使大肠杆菌内毒素所致的中毒性休克家兔血压降低的动物数减少，存活时间延长。本品可使冠脉结扎犬的缺血性心电图改善。本品还可使正常麻醉犬的心率减慢，血压、左室作功、心输出量及冠脉流量增加，使心肌耗氧量降低。一般药理学研究显示，本品可使正常麻醉大鼠的血压降低，心率减慢，心电图QT间期延长。

【贮　　藏】密封，遮光。

【包　　装】低硼硅玻璃管制注射剂瓶包装，8瓶/盒。

【有 效 期】30个月。

【执行标准】国家食品药品监督管理局标准YBZ07062006-2009Z-2011

【批准文号】国药准字Z20060463

【生产企业】

天津天士力之骄药业有限公司
TIANJIN TASLY PRIDE PHARMACEUTICAL CO.,LTD.
生产地址：天津市北辰科技园区
邮政编码：300410
电话号码：**8008189818 4006189818** 022-26736290
传真号码：022-26736275
注册地址：天津市北辰科技园区
网　　址：http://www.tasly.com

Figure S28: Specification of *Yiqifumai* injection.
